# 2-DE proteomics analysis of drought treated seedlings of *Quercus ilex* supports a root active strategy for metabolic adaptation in response to water shortage

**DOI:** 10.3389/fpls.2015.00627

**Published:** 2015-08-14

**Authors:** Lyudmila P. Simova-Stoilova, Maria C. Romero-Rodríguez, Rosa Sánchez-Lucas, Rafael M. Navarro-Cerrillo, J. Alberto Medina-Aunon, Jesús V. Jorrín-Novo

**Affiliations:** ^1^Agricultural and Plant Biochemistry and Proteomics Research Group, Department of Biochemistry and Molecular Biology, University of CordobaCordoba, Spain; ^2^Department of Forestry Engineering, School of Agricultural and Forestry Engineering, University of Coìrdoba, Agrifood Campus of International ExcellenceCoìrdoba, Spain; ^3^Computational Proteomics, Proteomics Facility, Centro Nacional de Biotecnología – CSICMadrid, Spain

**Keywords:** holm oak, roots, drought, recovery, proteomics

## Abstract

Holm oak is a dominant tree in the western Mediterranean region. Despite being well adapted to dry hot climate, drought is the main cause of mortality post-transplanting in reforestation programs. An active response to drought is critical for tree establishment and survival. Applying a gel-based proteomic approach, dynamic changes in root proteins of drought treated *Quercus ilex* subsp. Ballota [Desf.] Samp. seedlings were followed. Water stress was applied on 20 day-old holm oak plantlets by water limitation for a period of 10 and 20 days, each followed by 10 days of recovery. Stress was monitored by changes in water status, plant growth, and electrolyte leakage. Contrary to leaves, holm oak roots responded readily to water shortage at physiological level by growth inhibition, changes in water status and membrane stability. Root proteins were extracted using trichloroacetate/acetone/phenol protocol and separated by two-dimensional electrophoresis. Coomassie colloidal stained gel images were analyzed and spot intensity data subjected to multivariate statistical analysis. Selected consistent spots in three biological replicas, presenting significant changes under stress, were subjected to MALDI-TOF mass spectrometry (peptide mass fingerprinting and MS/MS). For protein identification, combined search was performed with MASCOT search engine over NCBInr Viridiplantae and Uniprot databases. Data are available via ProteomeXchange with identifier PXD002484. Identified proteins were classified into functional groups: metabolism, protein biosynthesis and proteolysis, defense against biotic stress, cellular protection against abiotic stress, intracellular transport. Several enzymes of the carbohydrate metabolism decreased in abundance in roots under drought stress while some related to ATP synthesis and secondary metabolism increased. Results point at active metabolic adjustment and mobilization of the defense system in roots to actively counteract drought stress.

## Introduction

Forest trees are of enormous ecological and economic value in global and local scale. They will be among the species most harmfully affected by the predicted climate changes with frequent temperature and precipitation extremes; however for a number of reasons our knowledge of the biochemical mechanisms to counteract inevitable environmental stresses is still very limited, especially concerning trees (Plomion et al., 2006; Abril et al., 2011). *Quercus ilex* is a dominant tree in western Mediterranean region, one of the main plant species of the so-called savannah-type woodland ecosystems (dehesas) which cover more than 4 million ha in western Mediterranean and northern African countries (David et al., 2007; Corcobado et al., 2013). Holm oak has double importance both from ecological and economic points of view, the latter coming from the fact that its acorns are major constituents in the diet of free range domestic animals; the rich nutrient composition and tannins of acorns give the original and specific taste of local meat products (David et al., 2007). According to climate model simulations, the Mediterranean region is expected to be a hot-spot which will be particularly affected by long term drought and warming episodes (Giorgi and Lionello, 2008).

Holm oak is very well adapted to dry hot climate, due to morphological particularities like a deep and well-structured root system with relatively large surface area and rapid development, which allows efficient capture of water from deepest soil layers, and small evergreen sclerophylous leaves with minimal transpiration and economical water use efficiency (David et al., 2007; Tsakaldimi et al., 2009). More biomass in this species is allocated in roots, forming larger below-ground starch and lipid reserves (Sanz-Pérez et al., 2009). Physiologically, holm oak is considered to be a typical water-spender species which maintains low leaf water potential. Stomatal closure upon water stress does not inhibit carbon assimilation in holm oak contrary to plants with water-saving strategy like pines (Baquedano and Castillo, 2006). It is predicted that the natural habitats of *Quercus ilex* will not be greatly affected or will even be expanded in the context of future climate changes (David et al., 2007; Bussotti et al., 2014). The overall good adaptation potential and huge economic importance of holm oak make it principal and valuable species for Mediterranean reforestation programs, which fostered research on *Quercus ilex* variability and stress response, especially at the proteome level (Jorge et al., 2006; Echevarría-Zomeño et al., 2009; Valero Galván et al., 2011, 2013). Main limitation in reforestation practices is the very low field survival rate of planted seedlings. Early seedling establishment is an extremely vulnerable phase of plant development and drought is considered to be the main cause of mortality post-transplanting (Navarro Cerrillo et al., 2005; Tsakaldimi et al., 2009). Besides, prolonged drought weakens tree defense systems against fungal pathogens. The forest decline is attributed to fungal attacks (e.g., *Hypoxylon mediterraneum* (De. Not.) Mill., *Biscogniauxia mediterranea* (De Not.) (Kuntze), or *Phytophthora cinnamomi* Rands.) after severe drought combined with high temperatures (Corcobado et al., 2013; Sghaier-Hammami et al., 2013). An active response to drought is critical for tree establishment and survival, however, our knowledge concerning the biochemical mechanisms of trees to counteract drought stress is still very limited, especially at the protein level (Abril et al., 2011).

Drought is one of the most deleterious abiotic stresses with respect to plant survival (Wang et al., 2003). Plants counteract drought using a combination of survival strategies like drought escape (through adjustment in the developmental program and ending reproductive cycle before severe drought development), drought avoidance (maintaining the internal water balance under drought conditions mainly through morphological and physiological adjustments), and drought tolerance (coping with water limitation mainly at cellular and biochemical level), which are subordinated to the natural climatic variations (Dolferus, 2014). In the genus *Quercus* for example, the rapid spring growth and allocation of high quantity of carbon reserves into roots could be regarded as elements of drought avoidance strategy—to develop robust root system and gain access to water in deeper soil layers (Sanz-Pérez et al., 2009). Survival under prolonged drought is more linked to tolerance strategies at the cellular level. Among them should be mentioned: synthesis of compatible solutes, enhanced protection against oxidative damage, protection of membranes, and proteins from denaturation through dehydrins and chaperones, degradation of unnecessary proteins and reusing the building blocks, and others (Wang et al., 2003; Dolferus, 2014). Roots are the plant part directly and primarily exposed to soil drought, so roots play a primordial role in water stress sensing and response. Besides the general anchoring and supportive function, root as a tissue provide water and mineral nutrients to all plant parts, and produce hormones for stress signaling (Ghosh and Xu, 2014). Root functions are indeed impeded upon severe water stress. Root tissue is very actively engaged in the adaptation to drought; for example water scarcity could stimulate root growth contrary to the aboveground plant part where growth is usually inhibited (Yamaguchi and Sharp, 2010). Contrasting metabolic changes have been reported in roots compared to shoots under drought stress—shoots have been metabolically inactivated and have had lower concentrations of sugars, amino acids, nucleosides, whereas roots have been metabolically activated, and more primary metabolites have been allocated to/synthesized in roots under water stress (Gargallo-Garriga et al., 2014). Increasing number of studies is accumulating now concerning root drought response at physiological, molecular biology, biochemical and proteomic levels (reviewed in Ghosh and Xu, 2014). As proteins are the main and direct executors of cellular functions, more pronounced impact of proteomic studies is recently observed in stress research on roots (Sengupta et al., 2011; Swigonska and Weidner, 2013; Oh and Komatsu, 2015). Proteomics along with metabolomics could be very useful tools in the so-called next-generation phenotyping methods of screening for stress tolerance (Dolferus, 2014). Proteomic studies on *Quercus* face the common problem with orphan species with non-sequenced genome which could be solved by homology-driven cross-species identifications based on high similarity to proteins from other plant species (Valero Galván et al., 2011). Preliminary studies of drought response in 1-year old plantlets of *Quercus ilex* using a proteomics approach have been performed in our group, analysing the changes in the leaf protein profile (Jorge et al., 2006; Echevarría-Zomeño et al., 2009).

The aim of the present study was to follow the dynamic changes in root proteins of drought treated *Quercus ilex* seedlings at very early developmental stage, applying a gel-based proteomic approach, and to relate proteome changes to stress severity and to recovery from stress, thus, to build a picture on the functional meaning of the observed protein changes.

## Material and methods

### Plant material and stress treatment

Holm oak (*Quercus ilex* subsp. Ballota [Desf.] Samp.) acorns were harvested on place in December 2011 from a region near Cerro Muriano (20 km apart from Cordoba). Mature fruits were dropped down directly from the trees, minimizing contact with soil. Acorns were immediately transported to the laboratory in tightly closed plastic bags, washed, surface-sterilized for 10 min in 10% sodium hypochlorite solution, extensively washed again, and inspected for worm damage. Healthy acorns were blotted dry, put in clean plastic bags and stored at 4–8°C. Spontaneously germinated acorns after 3 months of storage in cold were used in this study. Plants were grown in individual containers (330 ml volume) in perlite at growth chamber conditions: 12/12 h photoperiod, fixed light (360 μE.m^−2.^s^−1^), 24/20°C day/night temperature and 70% air humidity, at optimal water supply (120 ml tap water per 30 g dry perlite). Water stress was applied on 20 days-old holm oak seedlings with developed 9 ± 3 leaves on randomly selected sets of 12 plants, by carefully placing plants into new containers filled with perlite which was wetted with limited water quantity (40 ml water per 30 g dry perlite). Water limitation treatment was for a period of 10 and 20 days, followed by a 10 days recovery phase from stress by restoring the optimal water supply. Sufficient or limited water quantity was maintained by gravimetric measurements and daily compensation for the loss of water due to evapotranspiration. Sampling was performed at the appropriate time points—roots from drought treated plants for 10 (D10) and 20 (D20) days, and after 10 days recovery from 10 or 20-days water limitation period (R10, R20), with the respective age controls (C0—at the treatment beginning; C10—for D10, C20—for R10 and D20, and C30—for R20). Water status and electrolyte leakage were monitored on fresh plant material. For proteomic analyses, roots were quickly but thoroughly rinsed with distilled water, blotted dry, quick-frozen in liquid nitrogen, ground to fine powder and stored at −80°C.

### Stress estimation parameters—plant growth, water status, electrolyte leakage

Stress was monitored by changes in water status, growth, and electrolyte leakage. These parameters were assessed as previously described (Simova-Stoilova et al., 2008). Time course changes were followed in the water content of roots and leaves along with observations for any visible changes in plants. Biomass reduction and recovery growth were registered gravimetrically. Root and shoot fresh weight was taken at sampling from all the sets of plants (*n* = 12). Water content was measured in 3 individual plants per treatment, calculated according to the formula (FW-DW)/FW where FW is fresh weight, DW—dry weight of the same sample by drying it at 70°C to constant weight for 48 h, and was expressed in percentage. Water deficit in roots and leaves was estimated as (TW-FW)/TW where TW is the turgid weight after floating the tissues for 4 h at room temperature in deionized water, and expressed in percentage. Electrolyte leakage was estimated by measuring the conductivity of the effusate solution from intact tissue, kept for 4 h at room temperature in deionized water, relative to conductivity of the effusate from the same tissue after boiling it for 10 min and cooling down.

### Protein extraction and 2-DE separation

Root samples after 10 and 20 days of drought (D10 and D20) with the respective age controls (C10, C20) and 10-days recovery after 10 days of drought (R10) were analyzed by gel-based proteomics. Proteins were extracted from 3 biological replicas (each replica from 3 individual plants) according to the protocol of Wang et al. (2006) using TCA/acetone-phenol-methanol. Protein content in samples was estimated by the method of Bradford (1976) with bovine serum albumin as a standard. Samples were isoelectrofocused in the range of pI 5–8 using a Protean IEF Cell system (Bio-Rad, Hercules, CA, USA), 17 cm IPG strips, at 400 μg protein load per strip, active rehydration for 16 h at 50 V, rapid voltage ramp to 10,000 V, 50,000 Volt-hours in total, 500 V maintaining focused state. The second dimension was run at 12% SDS (PROTEAN® Plus Dodeca Cell, Bio-Rad, Hercules, CA, USA) and gels were double stained with colloidal Coomassie. Broad range molecular weight standards (Bio-Rad, Hercules, CA, USA) run by side in the same gel were used for estimation of MW.

### Image analysis and selection of spots of interest

Images of the gels were captured with a GS-800 densitometer (Bio-Rad, Hercules, CA, USA) and analyzed applying PDQuest software (Bio-Rad, Hercules, CA, USA). Ten-fold over background criterion was used to assess presence/absence of a spot. Data on normalized spot volumes were exported and subjected to multivariate statistical analysis including sample clustering, ANOVA and Principal component analysis using a free online-based software (NIA arrays analysis tools, http://lgsun.grc.nia.nih.gov/ANOVA/index.html). Prior to statistical analysis the normalized values were log transformed to reduce dependence between abundance and standard deviation. Data were statistically analyzed by ANOVA using the following settings: error model max (average, actual), 0.01 proportions of highest variance values to be removed before variance averaging, 10° of freedom for the Bayesian error model, 0.05 FDR threshold, and zero permutations. The principal component analysis (PCA) settings were: covariance matrix type, 4 principal components, 1.5–fold change threshold for clusters, and 0.5 correlation threshold for clusters. PCA results were represented as a biplot, with consistent proteins in those experimental situations located in the same area of the graph. Selected variable spots of interest (90 in total), well defined and presenting statistically significant changes (drought and/or recovery compared to the respective age controls), with at least 1.5-fold difference in abundance ratio, were manually cut for subsequent MS analysis.

### Mass spectrometry analysis, protein identification, and functional annotation

The MALDI-TOF/TOF Mass Spectrometry and Protein identification analysis was carried out in the UCO-SCAI proteomics facility, a member of Carlos III Networked Proteomics Platform, ProteoRed-ISCIII. The excised spots of interest were digested with porcine trypsin (sequencing grade) and loaded onto a MALDI plate, by using a ProPrep II station (Digilab Genomic Solutions Inc., Cambridgeshire, UK). The gel specimens were destained twice over 30 min at 37°C with 200 mM ammonium bicarbonate/40% acetonitrile. Gel pieces were then subjected to three consecutive dehydratation/rehydratation cycles with pure acetonitrile and 25 mM ammonium bicarbonate in 50% acetonitrile, respectively, and finally dehydrated for 5 min with pure acetonitrile and dried out over 4 h at room temperature. Then, 20 μl trypsin, at a concentration of 12.5 ng/μl in 25 mM ammonium bicarbonate was added to the dry gel pieces and the digestion proceeded at 37°C for 12 h. Peptides were extracted from gel plugs by adding 1 μl of 10% (v/v) trifluoracetic acid (TFA) and incubating for 15 min. Then, extracted peptides were desalted and concentrated by using μC-18 ZipTip columns (Millipore, Billerica, MA, USA) and were directly loaded onto the MALDI plate using α-cyano hydroxycinnamic acid as a matrix. Mass analysis of peptides (MS) of each sample was performed with a MALDI-TOF/TOF 4800 Proteomics Analyzer (Applied Biosystems, Foster City, CA, USA) mass spectrometer in the m/z range 800–4000, with an accelerating voltage of 20 kV. Spectra were internally calibrated with peptides from trypsin autolysis (M^+^H^+^ = 842.509, M^+^H^+^ = 2211.104). The most abundant peptide ions were then subjected to fragmentation analysis (MS/MS), providing information that can be used to determine the peptide sequence. Proteins were assigned identification by peptide mass fingerprinting and confirmed by MS/MS analysis. Mascot 2.0 search engine (Matrix Science Ltd., London, UK; http://www.matrixscience.com) was used for protein identification running on GPS ExplorerTM software v3.5 (Applied Biosystems, Foster City, CA, USA) over non-redundant NCBI protein and Uniprot databases. The following parameters were allowed: taxonomy restrictions to Viridiplantae, one missed cleavage, 100 ppm mass tolerance in MS and 0.5 Da for MS/MS data, cysteine carbamidomethylation as a fixed modification, and methionine oxidation as a variable modification. The confidence in the peptide mass fingerprinting matches (*p* < 0.05) was based on the MOWSE score, and confirmed by the accurate overlapping of the matched peptides with the major peaks of the mass spectrum. Proteins with statistically significant (*p* < 0.05) hits were positively assigned identification after considering Mr and pI values. Annotation of their biological function was consistent with Bevan et al. (1998). To predict the most probable intracellular localization of proteins, results from different software were compared—WolfPSORT (Horton et al., 2007, http://www.genscript.com/psort/wolf_psort.html), TargetP (Emanuelsson et al., 2000, http://www.cbs.dtu.dk/services/TargetP/), PredSL (http://aias.biol.uoa.gr/PredSL/input.html, Petsalaki et al., 2006), MultiLoc (http://abi.inf.uni-tuebingen.de/Services/MultiLoc/, Höglund et al., 2006). To construct a heat map the identified protein intensity, values were log10 transformed (base-10 logarithm of 1 + mean intensity values) and mean-centered to rescale them. Hierarchical clustering of samples and proteins were performed using MeV4.8 (http://www.tm4.org/mev.html) with agglomeration method set to average and the distances were calculated based on Pearson's correlation.

### Data submission

Starting from each individual search (Mascot.dat file), the identified spots were translated to PRIDE XML using the PRIDE Converter 2.0 software (Côté et al., 2012). A total of 90 PRIDE XML files together with the corresponding raw MS files, mzXML peak lists and the Mascot search results (.dat file) were submitted to the ProteomeXchange repository (Vizcaíno et al., 2014) following the ProteomeXchange submission guidelines. Data are available with identifier PXD002484.

## Results

### Holm oak morphological and physiological response to drought

**Figure 1:**
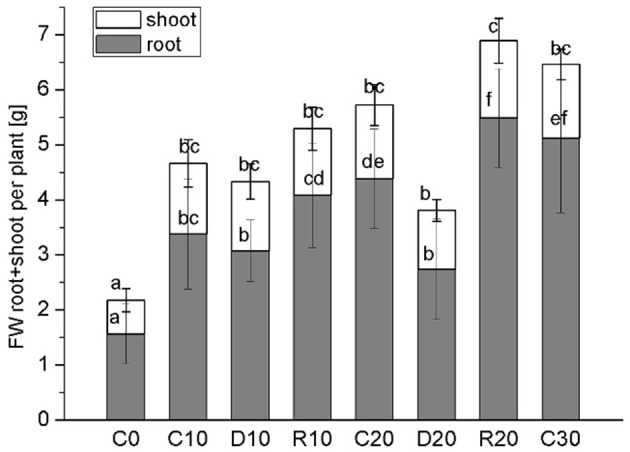
**Plant growth parameters**. Dark columns‘ part—root FW, white columns’ part—shoot FW. Mean values (*n* = 12) are given. Vertical bars represent standard deviations. C-control plants, C0, C10, C20, C30—the respective age controls (of days of treatment). D—plants subjected to water limitation treatment for 10 days (D10) or 20 days (D20). R—recovery by resuming optimal water supply for 10 days after 10 days (R10) or 20 days (R20) of water stress. Different letters above columns denote statistically significant differences.

**Figure 2:**
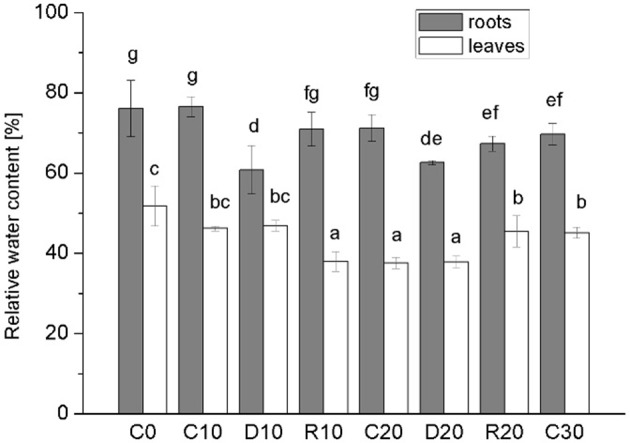
**Relative water content**. Separate columns for roots (dark) and leaves (white). Mean values (*n* = 3) are given. Vertical bars—standard deviations. C-control plants, C0, C10, C20, C30—the respective age controls (of days of treatment). D—plants subjected to water limitation treatment for 10 days (D10) or 20 days (D20). R—recovery by resuming optimal water supply for 10 days after 10 days (R10) or 20 days (R20) of water stress. Different letters above columns denote statistically significant differences.

In this study water stress was imposed on 20 days-old holm oak plantlets with well-developed root system consisting of small white tip part and larger brown lignified part, and well developed 9 ± 3 leaves. At this time the tissue linking cotyledon and plantlet was practically desiccated, and even lost somewhere, thus, cotyledons were not considered as influencing the plantlet. Water limitation was maintained for a period of 10 and 20 days, each followed by 10 days recovery. Appropriate age controls were used for comparison of stress treatments and recovery—C20 was for R10 and D20, C30 was for R20. The experimental design is presented schematically in Supplementary Material Figure S1. Leaf number was not significantly increased during the whole experimental period both in control and in treated plants. Stressed plants had longer and thinner roots with diminished white root part, which was more evident after 20 days of treatment and was not restored in recovery from the longer stress. Photos of control, stressed and recovered plants are presented in Supplementary Material Figure S2. Stress development was monitored by changes in plant growth parameters (Figure 1, dark columns ‘part—root FW, white columns’ part—shoot FW), in water status [relative water content Figure 2—separate columns for roots (dark) and leaves (white columns), and water deficit—Table 1 left part], and in electrolyte leakage (Table 1 right part) as an indicator of membrane damage. Biomass was significantly reduced only after 20 days of stress, more in roots (by 37.5%) than in shoots (by 20.9%). Water content in control plants was in the limits: root white part—87–78% (slightly diminishing with plant age), leaves—38–45%. Drought stress induced change in relative water content only in roots (diminution by about 10–15%), not in leaves. In roots, this parameter did not decrease further with stress prolongation and was completely restored in recovery. Water deficit increased only in roots after drought treatment, along with increase in membrane instability. A 2-fold rising in relative electrolyte leakage (EL) was registered in roots under 10 days of drought stress. In leaves EL remained very low, possibly linked to establishment of xeromorphic leaf structure. Based on these data, an active strategy for metabolic adaptation to drought is expected to be found in root tissue at the proteome level.

**Table 1:** **Water deficit (left part), and electrolyte leakage (right part)**.

**Parameter**	**Water deficit %**		**Relative electrolyte leakage %**	
**Tissue**	**Roots**	**Leaves**	**Root tips**	**Leaves**
C0	10.1 ± 3.1	10.9 ± 1.3	16.4 ± 9.5	9.9 ± 2.9
C10	8.6 ± 4.2	10.7 ± 1.1	20.9 ± 6.7	6.8 ± 1.7
D10	29.3 ± 6.9	11.5 ± 1.7	41.9 ± 4.9	7.0 ± 0.9
Remark	3 times increase	n.s. change	2 times increase	n.s. change

*Mean ± standard deviations from three independent replicates are shown; n.s.-non-significant changes. C0-control at the beginning of the treatment, D10—water limitation treatment for 10 days, C10—the respective age control*.

### Quercus ilex root proteome changes after drought and recovery

**Figure 3:**
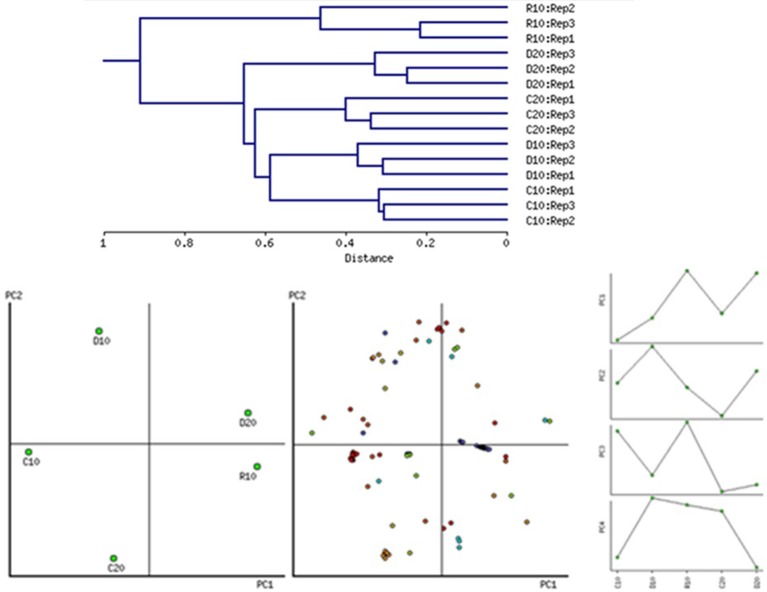
**Sample clustering on the whole dataset of 2-DE spot intensities according to tissue and treatment, principal component analysis biplot (PC1/PC2) and tendencies of changes for the first four PCs**. PC1 clearly separates variants depending on age while PC2 –on the treatment. The first four PCs contribute to 83.89% of the variation.

The protein extraction with modified TCA/acetone/phenol protocol resulted in protein yield of about 370–740 μg protein per g FW of root tissue (Table 2). Protein extraction was also made of samples recovered from D20, but in R20 the quantity of extracted protein dropped substantially. Five variants of samples were analyzed applying gel based proteomics—C10, D10, R10, C20, D20, in biological triplicates. Due to the insufficient protein yield, R20 was not studied. The 2-DE gel images of each of the variants (pI 5–8, and 12% SDS PAGE), 400 μg protein load, Coomassie colloidal staining) are shown in Figure S3, Supplementary. Images were analyzed with PDQuest software. Approximately 359 ± 9 consistent protein spots were clearly resolved on the gels. Concerning variability in abundance on the basis of spot volume ratio (treated to age control variants), relatively more spots were found to be decreased in abundance than increased, and more variability was found in recovery compared to drought treatment (Table 2). Sample clustering and PC analysis data (Figure 3) clearly separated the five sample variants – C10, D10, R10, C20, and D20.

**Table 2:** *****Quercus ilex*** root proteome changes after drought treatment and recovery**.

**Variants**	**Protein yield (μg.g^1^FW)**	**Total number of spots**	**Consistent spots**	**Number of Variable spots >2-fold/>1.5-fold change**	**Spots up under drought >2-fold change/>1.5-fold change**	**Spots down under drought >2-fold change/>1.5-fold change**
C10	370	384	364			
D10	580	364	353	75/156	34/75	41/81
R10	582	447	361	104/184	32/66	72/118
C20	633	391	369			
D20	740	358	347	80/148	30/58	50/90

*Variable spots (>2-fold/>1.5-fold change) are related to the respective age controls. C-control plants, C10, C20—the respective age controls (of days of treatment). D—plants subjected to water limitation treatment for 10 days (D10) or 20 days (D20). R–recovery by resuming optimal water supply for 10 days after 10 days of drought (R10), the age control in this case is C20*.

### Identification of differently abundant spots and patterns of protein changes

**Figure 4:**
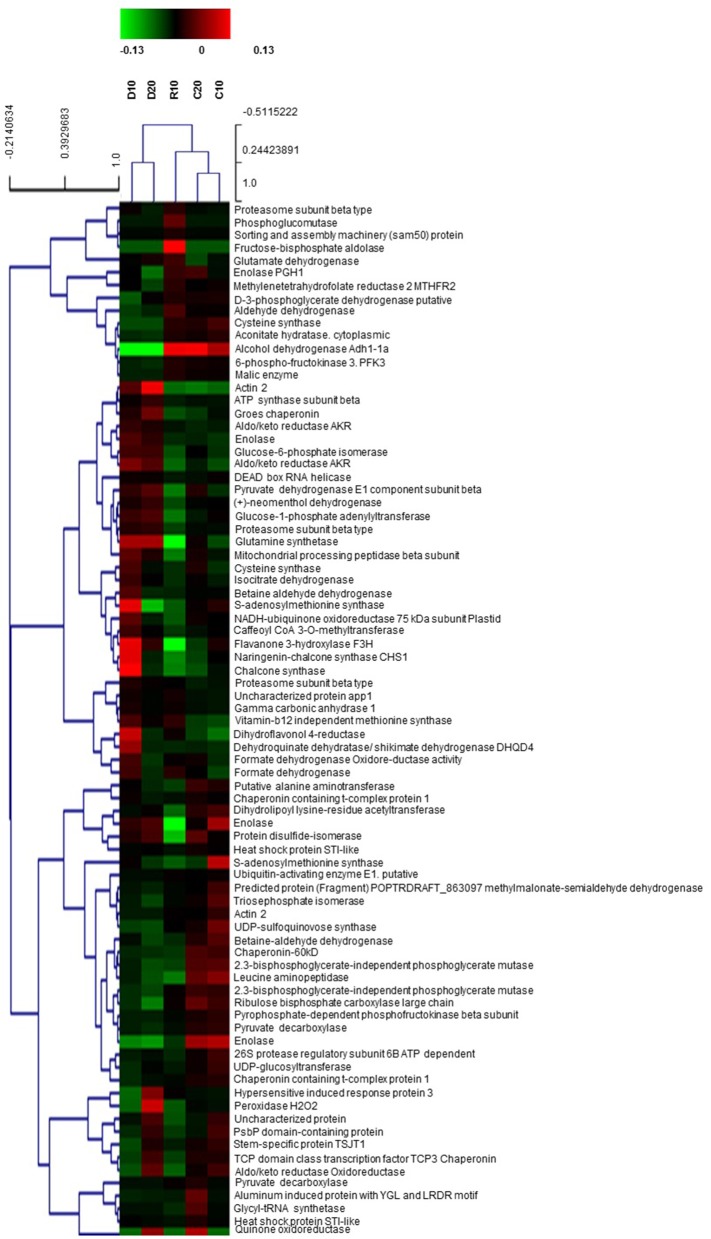
**Heat map built on the basis of normalized spot abundancy ratios—treatment to the respective age control**. D—plants subjected to water limitation treatment for 10 days (D10) or 20 days (D20). R—recovery by resuming optimal water supply for 10 days after 10 days of drought (R10); C10, C20—control plants (the respective age controls of days of treatment).

**Figure 5:**
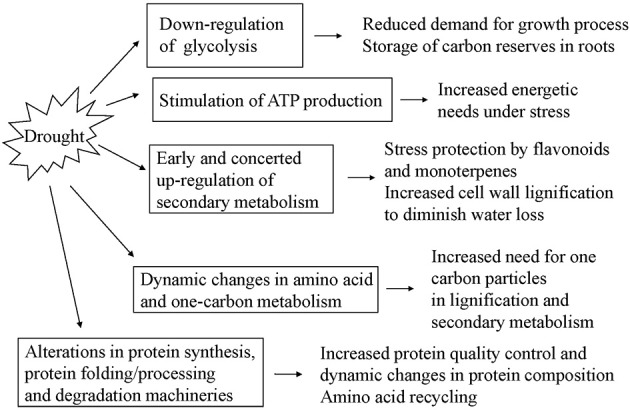
**Summary diagram of molecular mechanisms involved in *Quercus ilex* root response to water limitation**.

Selected well defined spots, presenting significant changes under stress (at least 1.5-fold change compared to controls) and consistent in the three biological replicas, were subjected to MALDI-TOF PMF and MS/MS. For protein identification, combined search was performed with MASCOT search engine over NCBInr Viridiplantae and Uniprot databases. Detailed tables with identified protein species and their most probable subcellular location are presented in the Supplementary Material (Supplementary Tables S1, S2). There were five cases of low score unreliable identification and three cases of 2–3 different proteins identified in the same spot, which were excluded from these tables. In spite of the fact that *Quercus ilex* is an orphan species and identification was mainly based on homology, the majority of reliable hits for one given spot were for the same protein in different plant species. A few of the proteins were detected in more than one spot, like actin 2, enolase (EC 4.2.1.11), betaine aldehyde dehydrogenase (EC 1.2.1.8), cysteine synthase (EC 2.5.1.47), pyruvate decarboxylase (EC 4.1.1.1), but with some exceptions they presented similar trends of changes. Differences in protein abundance are clearly distinguished in the heat map built on the basis of normalized spot abundancy ratios of treatment to the respective age control (Figure 4). Different patterns of changes were found when looking at the identification results; however, the majority of spot abundance changes presented the same trend at drought stress irrespective treatment duration—D10 or D20. The following types of dynamic changes were observed—changes in abundance under stress unrelated to how long was the treatment (43 protein spots—24 up and 19 down), increase or decrease in abundance only at D10 (14 protein spots—8 up and 6 down in abundance), decrease only at D20 (2 protein spots), spot abundance changes in different directions comparing D10 and D20 (4 protein spots), prominent abundance changes in recovery, mainly diminution (8 spots), and changes beyond detection level, so-called qualitative changes (11 protein spots). Among the identified proteins with earlier and reversible increase in abundance under drought followed by recovery were found many enzymes related to secondary metabolism such as: caffeoyl CoA 3-O-methyl transferase (EC 2.1.1.104), chalcone synthase (2 spots, EC 2.3.1.74), shikimate dehydrogenase (EC 1.1.1.25), quinone oxidoreductase (EC 1.6.5.5), as well as ubiquitin activating enzyme E1 (EC 6.3.2.19), DEAD box RNA helicase (EC 3.6.4.13), betaine aldehyde dehydrogenase (EC 1.2.1.8). Down-accumulated in D10 were some inducible and hormone responsive proteins, glycyl-tRNA synthetase (EC 6.1.1.14), D-3-phosphoglycerate dehydrogenase (L-serine biosynthesis, (EC 1.1.1.95). There were not up-accumulated proteins typical only for prolonged drought treatment but 2 spots presented decrease in abundancy only at D20—methylmalonate-semialdehyde dehydrogenase (EC 1.2.1.27), and methylene tetrahydrofolate reductase (EC 1.5.1.20), a cytoplasmic enzyme member of one-carbon metabolism. A few proteins had opposite trends of changes comparing D10 and D20—peroxidase (EC 1.11.1.7)—D10 decrease, D20 increase, S-adenosylmethionine synthase (one-carbon metabolism, 2 spots, (EC 2.5.1.6)—D10 increase, D20 decrease, and the mitochondrial NADH-ubiquinone oxidoreductase (EC 1.6.5.3)—D10 increase, D20 decrease. The following proteins presented prominent changes in abundance after recovery, mainly diminution: Pyruvate dehydrogenase E1 (EC 1.2.4.1) subunit beta, component 3 of pyruvate dehydrogenase complex, enolase (EC 4.2.1.11), protein disulfide-isomerase (EC 5.3.4.1), STI like HSP (2 spots). The only protein spot with increase in recovery was proteasome subunit beta (EC 3.4.25.1). The most interesting qualitative differences were in one cysteine synthase isoform (EC 2.5.1.47), which spot was below detection under drought; however, there was another spot identified also as cysteine synthase with increased abundance under drought. Identified proteins were classified into functional groups according to Bevan et al. (1998): primary and secondary metabolism, protein biosynthesis and proteolysis, defense against biotic stress, cellular protection against abiotic stress, intracellular transport. In Table 3 are summarized the main functional groups and subgroups and the tendencies of changes in individual proteins within groups. It is seen that several enzymes of the carbohydrate metabolism were decreased in abundance in roots under drought stress while some related to ATP synthesis and secondary metabolism were increased. Results point at active metabolic adjustment and mobilization of the defense system in roots to actively counteract stress. A summary diagram of molecular mechanisms involved in *Quercus ilex* root response to water limitation is presented in Figure 5.

**Table 3:** **Main functional groups and subgroups of identified proteins and the tendencies of changes in individual proteins within groups**.

**Protein name/Species of closest homology**	**SSP**	**Accession No**.	**Mr/pI**		**Mascot scores**		**Spot volume ratio changes**			
			**Experimental**	**Theoretical**	**Protein score/C.I**	**Total ion score/ C.I**.	**D10/C10**	**R10/C20**	**D20/C20**	**C20/C10**
**GLYCOLYSIS AND GLYOXYLATE BYPASS**										
**Glucose-6-phosphate isomerase**										
*Pop.trichocarpa*	1401	tr|B9GV29|B9GV2	45.56/5.8	68.1/5.55	367/100	275/100	1.301	0.835	1.415^*^	0.914
**Phosphofructokinase**										
*Citrus paradisi*	4707	tr|Q9ZST3|Q9ZST	65.7/6.71	62.23/6.1	163/100	129/100	0.672^*^	0.722	0.584^*^	1.137
**Phosphoglycerate mutase**										
*Ricinus communis*	1702	sp|P35493|PMGI_	70.51/5.9	61.01/5.5	249/100	205/100	0.532^*^	0.356^*^	0.312^*^	1.086
	2701	tr|B9S1V6|B9S1V	69.86/6.1		306/100	255/100	0.549^*^	0.813	0.464^*^	0.967
**Enolase**										
*Jatropha curcas*	1504	tr|E6NU46|E6NU4	55.7/5.92	52.69/6.3	384/100	319/100	1.799	1.089	1.719^*^	0.911
*Spinacia oleracea*	1507	tr|Q9LEE0|Q9LEE	55.25/6.0	48.37/5.5	187/100	134/100	1.121	0.207^*^	0.851	1.463
*Alnus glutinosa*	3503	sp|Q43321|ENO_A	56.84/6.4	47.79/5.4	460/100	396/100	0.746	1.362	0.477^*^	0.718
*Ricinus communis*	5602	tr|B9R9N6|B9R9N	58.52/6.8	48.15/5.7	461/100	400/100	0.243^*^	0.409^*^	0.181^*^	1.099
**Glucose-1-phosphate adenylyl transferase**										
*Glycine max*	1407	tr|I1JJP2|I1JJP	51.01/6.03	56.71/6.3	574/100	445/100	1.565	0.393^*^	1.413	1.235
**6-phospho-fructokinase 3 PFK3**										
*Arabidopsis thaliana*	7605	sp|Q94AA4|K6PF3	61.41/7.4	54.09/6.61	317/100	268/100	0.609	1.174	0.578^*^	0.964
**Phosphoglucomutase**										
*Populus tremula*	3707	sp|Q9ZSQ4|PGMC_	74.14/6.4	63.37/5.5	240/100	145/100		R+		
**Triosephosphate isomerase**										
*Solanum tuberosum*	5106	tr|Q3HRV9|Q3HRV	20.82/6.8	27.92/5.9	378/100	321/100	0.564	0.379^*^	0	2.005^*^
**Malic enzyme**										
*Oryza brachyantha*	7701	tr|J3L3Y9|J3L3Y	76.28/7.1	71.65/8.8	123/100	116/100	0	1.564	0	0.99
**Fructose-bisphosphate aldolase, cytoplasmic isozyme 2**										
*Pisum sativum*	7306	sp|P46257|ALF2_	43.39/7.43	38.64/6.77	104/100	83/100		R+		
**Aconitate hydratase, cytoplasmic**										
*Cucurbita maxima*	5806	sp|P49608|ACOC_	98.54/6.9	98.57/5.7	166/100	118/100	0.441^*^	0.948	0.305^*^	1.222
**TRICARBOXYLIC ACID CYCLE**										
**Isocitrate dehydrogenase**										
*Ricinus communis*	2303	tr|B9SRZ2|B9SRZ	41.8/6.11	40.63/7.0	327/100	258/100	1.587^*^	0.74	1.447	0.712
**Pyruvate dehydrogenase E1 beta**										
*Pisum sativum*	1201	sp|P52904|ODPB_	38.03/5.8	38.99/5.9	251/100	234/100	1.085	0.681	1.522^*^	0.811
**Dihydrolipoyl lysine-residue acetyltransferase**										
*Arabidopsis thaliana*	1502	sp|Q5M729|OPD23	54.32/5.8	58.89/7.9	350/100	324/100	0.743	0.182^*^	0.701	1.227
**AMINO ACID METABOLISM**										
**Glutamine synthetase**										
*Camellia sinensis*	3302	tr|Q762D2|Q762D	40.11/6.3	39.41/5.5	206/100	188/100	1.301	0.751	1.476^*^	0.899
**Cysteine synthase**										
*Ricinus communis*	2102	tr|B9RET4|B9RET	31.66/6.2	34.44/ 5.5	409/100	369/100	1.255	1.048	1.288	0.671^*^
**Alanine aminotransferase**										
*Oryza sativasubsp. japonica*	0606	tr|Q7X7S9|Q7X7S	56.56/5.5	53.77/5.2	227/100	199/100	0.687^*^	0.332^*^	0.489^*^	0.885
**3-phosphoglycerate dehydrogenase**										
*Ricinus communis*	4702	tr|B9RYA3|B9RYA	64.92/6.5	63.35/7.6	210/100	162/100	0.511^*^	1.053	0.94	1.031
**Glutamate dehydrogenase**										
*Populus trichocarpa*	7402	tr|B9IPQ2|B9IPQ	45.2/7.15	44.72/6.84	338/100	281/100	1.729^*^	1.555^*^	1.308	1.587^*^
**ONE CARBON METABOLISM**										
**S-adenosylmethionine synthase**										
*Camellia sinensis*	2402	sp|Q9LDQ7|METK_	48.07/6.1	43.23/5.3	555/100	403/100	1.401	0.349	0.454^*^	2.449
*Elaeagnus umbellata*	2405	sp|Q9AT55|METK	47.84/6.2	43.56/5.5	731/100	563/100	1.579^*^	0.772	0.674	1.081
**Vit.b12 indep. methionine synthase**										
*Pop. trichocarpa*	6801	tr|B9HQI3|B9HQI	87.0/6.97	85.03/6.2	566/100	499/100	2.555	2.484	1.998^*^	0.889
**Formate dehydrogenase**										
*Quercus robur*	7302	tr|Q7XHJ0|Q7XHJ	43.52/7.1	40.85/6.5	509/100	365/100	1.291	1.299	0.966	0.763^*^
	7408	tr|Q7XHJ0|Q7XHJ	44.88/7.4		489/100	368/100	1.587	2.649^*^	1.361	0.531^*^
**Methylene tetrahydro folate reductase**										
*Arabidopsis thaliana*	4704	sp|O80585|MTHR	65.2/6.62	67.39/5.3	104/100	95/100	1.007	1.274	0.279^*^	1.114
**AMINE BIOSYNTHESIS**										
**Betaine aldehyde dehydrogenase**										
*Amaranthus hypochondr.*	1603	sp|O04895|BADH_	63.69/5.8	55.4/5.4	246/100	222/100	1.877^*^	0.77	0.775	1.009
*Corylus heterophylla*	1605	tr|E9NRZ9|E9NRZ	63.72/5.9	55.2/5.44	152/100	127/100	0.677	0.36	0.221^*^	1.404
**ETHANOL FERMENTATION**										
**Pyruvate decarboxylase**										
*Prunus armeniaca*	3703	tr|B0ZS79|B0ZS7	69.93/6.3	66.2/5.74	244/100	234/100	0.345^*^	1.54	0.818	0.451^*^
	3705		70.07/6.4		248/100	230/100	0.209^*^	0.348^*^	0	1.128
**SECONDARY METABOLISM**										
**Chalcone synthase**										
*Juglans nigra xJuglans regia Malus domestica*	6403	tr|Q42864|Q4286	46.52/7.1	42.9/6.3	323/100	235/100	2.609^*^	0.554^*^	0.927	1.317
	7401	tr|B8R6A2|B8R6A	46.7/7.12	42.79/5.7	494/100	396/100	2.472^*^	0.709	0.963	1.252
**(**+**)-Neomenthol dehydrogenase**										
*Arabid.thaliana*	0104	sp|Q9M2E2|SDR1_	25.81/5.5	33.07/5.4	83/99.97	68/99.997	1.342^*^	0.407^*^	1.556^*^	1.067
**Caffeoyl CoA 3-O-methyltransferase**										
*Betula platyphylla*	0105	tr|Q5I2D1|Q5I2D	23.07/5.5	27.95/5.4	433/100	325/100	1.718^*^	0.603^*^	1.051	1.151
**Flavanone 3-hydroxylase**										
*Ampelopsis grossedentata*	3303	tr|I6ZTY9|I6ZTY	40.46/6.4	41.2/5.3	840/100	606/100	1.994^*^	0.449^*^	1.106	1.252
**Dihydroflavonol 4-reductase**										
*P.suffruticosa*	6304	tr|G4WCQ6|G4WCQ	41.6/7.03	41.4/5.67	92/99.996	76/100	2.738^*^	2.012^*^	1.594^*^	0.731
**Shikimate dehydrogenase**										
*Populus trichocarpa*	6601	tr|B9HSF2|B9HSF	64.05/6.9	58.27/5.9	96/99.998	80/100	2.154^*^	1.066	1.105	0.95
**UDP-glucosyl transferase**										
*Ricinus communis*	4507	tr|B9S1I8|B9S1I	54.8/6.61	52.97/6.21	69/99.224	59/99.987	0	0.194^*^	0.382^*^	1.814^*^
**ATP SYNTHESIS**										
**ATP synthase subunit beta**										
*Vitis vinifera*	1601	tr|F6GTT2|F6GTT	58.71/5.7	59.3/5.9	941/100	741/100	1.284	0.943	1.724^*^	0.963
**NADH-ubiquinone oxidoreductase**										
*Zea mays*	3806	tr|B6U2J0|B6U2J	84.5/6.42	81.67/6.1	235/100	212/100	1.605^*^	0.388^*^	0.806	0.926
**Gamma carbonic anhydrase**										
*A. thaliana*	5107	sp|Q9FWR5|GCA1_	23.46/6.91	30.12/7.13	115/100	94/100	2.505	2.939^*^	1.991	0.712
**PROTEIN SYNTHESIS**										
**DEAD box RNA helicase**										
*Pisum sativum*	1509	tr|Q8H1A5|Q8H1A	50.52/5.5	47.14/5.39	310/100	202/100	1.784^*^	0.495	1.14	1.509^*^
**Glycyl-tRNA synthetase**										
*Arabidopsis lyrata*	3805	tr|D7KEM8|D7KEM	78.97/6.5	82.49/6.43	443/100	395/100	0.411^*^	0.558	0.878	0.466^*^
**PROTEIN FOLDING/PROCESSING**										
**Groes chaperonin**										
*Ricinus communis*	3007	tr|B9RR63|B9RR6	19.06/6.3	26.58/8.89	86/99.983	53/99.912	2.342^*^	0.43^*^	2.245^*^	1.634
**Protein disulfide-isomerase**										
*Nicotiana tabacum*	1202	tr|P93358|P9335	38.81/5.8	40.08/5.99	201/100	180/100	0.884	0.409^*^	1.2	0.834
**Chaperonin-60kD**										
*Ricinus communis*	1701	tr|B9S582|B9S58	66.69/5.7	61.48/6.2	393/100	332/100	0.646	0.606^*^	0.638	0.987
**heat shock protein STI-like**										
*Glycine max*	4809	tr|I1LGM2|I1LGM	77.99/6.6	65.86/6.26	67/98.652	54/99.95	0.632^*^	0.485^*^	0.991	0.795
	4810	tr|I1J9X8|I1J9X	78.27/6.6	66.91/6.07	68/99.023	50/99.85	0.405^*^	0.414^*^	0.912	0.356^*^
**Chaperonin,TCP-1**										
*Malus domestica*	3604	tr|D9ZJD1|D9ZJD	63.5/6.33	57.4/5.6	292/100	223/100	0.398^*^	0.379^*^	1.254	1.04
*Ricinus communis*	4705	tr|B9RSN1|B9RSN	70.46/6.6	59.56/ 6.08	362/100	238/100	0.917	0.77	0.692	0.854
*Ricinus communis*	5701	tr|B9SUJ3|B9SUJ	69.96/6.8	60.64/5.97	157/100	135/100	0.161^*^	0.509	0.462	1.138
**PROTEIN DEGRADATION**										
**Proteasome subunit beta type**										
*Glycine max*	2004	tr|C6SVE5|C6SVE	18.43/6.2	25.17/5.3	651/100	592/100	1.363	0.671	1.456^*^	1.013
*Ricinus communis*	5004	tr|B9SJ80|B9SJ8	20.5/6.92	30.6/7.6	58/90.457	47/99.745	1.58^*^	1.167	1.216	1.133
*Picea sitchensis*	7002	tr|A9NZ27|A9NZ2	19.97/7.4	29.59/5.58	224/100	203/100	1.281	1.723^*^	0.903	0.951
**Xaa-Pro aminopeptidase**										
*Solanum lycopersicum*	3803	tr|Q93X46|Q93X4	79.52/6.4	73.45/5.98	88/99.99	81/100	1.58	1.776^*^	1.341	0.923
**Mitochondrial processing peptidase**										
*Cucumis melo*	3607	tr|Q9AXQ2|Q9AXQ	62.57/6.5	58.9/6.56	656/100	575/100	1.25	0.703^*^	1.22^*^	0.894
**Leucine aminopeptidase**										
*Medicago truncatula*	2603	tr|G7JGP9|G7JGP	58.61/6.1	59.86/7.56	292/100	220/100	0.577	0.252^*^	0.363^*^	1.152
**26S protease regulatory subunit 6B**										
*M. truncatula*	1402	tr|G7IV48|G7IV4	51.86/5.9	46.58/5.4	540/100	335/100	0.786	0.552^*^	0.673^*^	1.322^*^
**Ubiquitin-activating enzyme E1**										
*Ric.communis*	1901	tr|B9SKZ1|B9SKZ	117.4/5.8	123.59/5.0	87/99.988	67/99.997	2.059^*^	0.758^*^	0	2.624^*^
**STRESS RESPONSE**										
**Aldo/keto reductase**										
*Manihot esculenta*	0203	tr|Q52QX9|Q52QX	35.02/5.6		232/100	210/100	4.2^*^	1.116	2.516^*^	1.422
	3205	tr|Q52QX9|Q52QX	38.15/6.3	38.03/6.38	325/100	246/100	2.025^*^	0.785	2.41^*^	0.738
	4201	tr|Q52QX9|Q52QX	38.37/6.51		327/100	296/100	0.341^*^	0.178^*^	1.203	1.444
**Alcohol dehydrogenase**										
*Pyrus communis*	6302	tr|G0Z9K2|G0Z9K	41.76/6.9	42.08/6.51	611/100	545/100	0.119^*^	1.149	0.091	0.889
**Short-chain dehydrogenase / reductase (SDR) family protein**										
*Solanum lycopers.*	0103	tr|K4AZG5|K4AZG	25.77/5.4	35.46/5.39	82/99.95	78/100	1.066	0.323^*^	1.185	1.675^*^
**Aldehyde dehydrogenase**										
*Vitis vinifera*	6602	tr|G9HQG0|G9HQG	60.16/6.98	52.53/6.18	77/99.86	63/99.993	0.464^*^	1.572^*^	0.668	1.039
**Peroxidase**										
*Linum usitatissimum*	0507	tr|Q43782|Q4378	48.17/5.4	38.63/8.07	77/99.865	68/99.997	0.332^*^	0.406^*^	3.295^*^	0.96
**Stem-specific protein TSJT1**										
*Ricinus Communis*	0005	tr|B9RTW0|B9RTW	20.5/5.54	25.5/5.56	77/99.87	70/99.998	0.189^*^	0.451^*^	0.906	1.298
**Hypersensitive induced response protein 3**										
*Triticum aestivum*	0108	tr|B6D9L4|B6D9L	24.68/5.67	31.8/5.68	231/100	202/100	0.395^*^	1.238	2.499^*^	0.949
**Al induced protein**										
*Arabidopsis thaliana*	2105	tr|Q9LIL3|Q9LIL	22.29/5.96	27.5/5.84	94/99.997	85/100	0.179^*^	0.837	0.755	0.305^*^
**CELL MOTILITY**										
**Actin 2**										
*Gossypium hirsutum*	0404	tr|B8YPL4|B8YPL	45.11/5.7	41.94/5.38	787/100	597/100	1.552	1.01	1.804^*^	1.052
*Annona cherimola*	0703	tr|H9A1W7|H9A1W	65.5/5.51	41.85/5.31	788/100	562/100	0	0.411	0	2.661
**PHOTOSYNTHESIS**										
**PsbP domain-containing protein**										
*M. truncatula*	0113	tr|G7J6G5|G7J6G	26.71/5.3	27.95/6.92	62/95.93	55/99.96	0.514	0.056^*^	0.889	3.077
**Oxygen-evolving enhancer protein**										
*Populus euphratica*	0109	sp|P84989|PSBO_	24.86/5.5	10.6/5.36	423/100	361/100	0	1.935	5.33^*^	0.605
**Rubisco LS**										
*Noronhia emarginata*	3706	tr|Q06QN8|Q06QN	66.65/6.4	53.05/6.22	197/100	101/100	0.354^*^	0.727	0	0.825

Spot volume ratio changes of variants: D—plants subjected to water limitation treatment for 10 days (D10) or 20 days (D20). R—recovery for 10 days after 10 days of drought (R10); C10, C20—control plants (the respective age controls of days of treatment). Statistically significant differences between control and treated variants or between age controls (p < 0.05) are indicated by asterix.

## Discussion

### Physiological changes in *Quercus ilex* roots under water limitation

Holm oak is regarded as relatively drought tolerant plant species, well adapted to Mediterranean type of climate. Its tolerance is due to the large well developed root system, the evergreen sclerophylous leaf anatomy, the very economic use of water resources, as well as some particularities in photosynthesis (David et al., 2007; Tsakaldimi et al., 2009). However, seedling establishment is one of the most stress vulnerable phases in plant development (Tsakaldimi et al., 2009). In our experimental system, much more plant biomass is allocated in roots than in shoots (2.5 to 3-fold more) at early seedling stage. Significant growth inhibition was detected after relatively long period of water limitation (20 days), both for roots and for shoots, which confirms the expected drought resilience of this tree species. Some reports emphasize the active root growth vs. shoot growth inhibition as an adaptive strategy for drought adaptation, which is reflected in changes in root to shoot biomass ratio (Yamaguchi and Sharp, 2010), while in other cases root growth is inhibited in response to progressive water stress (Sengupta et al., 2011), probably linked to stress duration and species peculiarities in stress tolerance. In the case of *Quercus ilex* seedlings, however, the total root biomass was more negatively affected by prolonged drought than the shoot biomass was, resulting in diminution in root to shoot ratio compared to the respective age controls. In the time course of the experiment, the root to shoot ratio in the age controls was constantly increasing, which supports the more active root growth compared to shoots. Besides root biomass diminution, prolonged drought leaded to changes in the root aspect—longer and thinner roots with less white tip part at D20, which may be linked to increased lignification as a response to water deficit. Increased degree of lignification in the basal part of root elongation zone has been reported in response to drought for other plant species (Yamaguchi et al., 2010). The difference in leaf and root response to water shortage was further supported by the observed changes in water status and membrane stability after drought stress, expressed mainly for the root system. Pronounced change in water status of roots subjected to drought stress, compared to leaves, is also documented for other plant species (Yoshimura et al., 2008; Wendelboe-Nelson and Morris, 2012).

### Secondary metabolism is activated in roots under drought treatment

Drought adaptation of plants requires complex rearrangements of the metabolism with interactions between several metabolic pathways. One of the most striking observations in our comparative study was the relatively early increase of several cytoplasmic enzymes engaged in secondary metabolism in roots under water stress, like: shikimate dehydrogenase (EC 1.1.1.25), naringenin-chalcone synthase (EC 2.3.1.74), flavanone 3-hydroxylase (EC 1.14.11.9), dihydroflavonol 4-reductase (EC 1.1.1.219), (+)-neomenthol dehydrogenase (EC 1.1.1.208), and caffeoyl CoA 3-O-methyltransferase (EC 2.1.1.104). Shikimate dehydrogenase, an enzyme of the shikimic acid pathway leading to biosynthesis of aromatic amino acids and simple phenolics, catalyzes the reversible NADP^+^-dependent reaction of 3-dehydroshikimate to shikimate. Chalcone synthase (or naringenin-chalcone synthase) is a plant enzyme in the initial step and central hub for the pathway of flavonoid biosynthesis, leading to production of flavanoids, isoflavonoid-type phytoalexins, and other metabolites with stress protective functions for plants. Other enzymes of the flavonoid biosynthesis pathway which are found to be up-accumulated in concert with chalcone synthase under drought were: flavanone 3-hydroxylase which catalyzes the stereospecific conversion of flavanones to dihydroflavonols, and dihydroflavonol reductase, which catalyzes the reduction of dihydroflavonols to leucoanthocyanins (Dao et al., 2011). Chalcone synthase is induced under different abiotic and biotic stresses like UV, wounding, herbivory, and microbial pathogens, resulting in the production of compounds with antimicrobial, insecticidial, and antioxidant activity (Selmar and Kleinwächter, 2013). Flavonoids interfere with hormone signaling by inhibiting polar auxin transport (Dao et al., 2011). Increasing evidence suggests that plants exposed to drought accumulate secondary metabolites, and a plausible explanation could be to protect cells from oxidative stress by consuming NADPH^+^ H^+^ for the synthesis of highly reduced precursors like aromatic amino acids, monoterpens, alkaloids (Selmar and Kleinwächter, 2013). Increased abundance under drought of the enzyme (+)-neomenthol dehydrogenase, which participates in monoterpenoid biosynthesis, observed in this study, could be linked to possible protective function of monoterpens. Caffeoyl-CoA 3-O-methyltransferase is engaged in the pathway of lignin biosynthesis. The accumulation of this enzyme under drought could be related to increased lignification of the cell wall—a modification in order to avoid water loss. Similar up regulation of Caffeoyl-CoA 3-O-methyltransferase in roots subjected to water stress is reported by several authors (Alam et al., 2010; Yamaguchi et al., 2010; Fulda et al., 2011). In concert with the changes in root growth (longer and thinner roots with less biomass) we observed an increase in abundance of actin 2 (component of microphilaments) as well as early decrease and late increase in peroxidase abundance—a cell wall cross-linking enzyme participating in cell wall lignification, defense against pathogen attack, and activated oxygen consumer. Increased content of peroxidase III in roots of wild watermelon under drought has been reported (Yoshimura et al., 2008).

### Carbon metabolism and energy production—glycolysis is down-regulated in roots in water deficit conditions while ATP synthesis is stimulated

In this study we observed concerted decrease in abundance of glycolytic enzymes—glucose-6-phosphate isomerase (EC 5.3.1.9), pyrophosphate-dependent phosphofructokinase (EC 2.7.1.90), 2,3-bisphospho glycerate-independent phosphoglycerate mutase (EC 5.4.2.12), as well as of the enzyme pyruvate dehydrogenase (EC 1.2.4.1)—mitochondrial enzyme which links the glycolysis metabolic pathway to the citric acid cycle. Decrease in the amount of the cytoplasmic aconitate hydratase (EC 4.2.1.3), an enzyme of the glyoxylate bypass in plants for utilization of fatty acids as a carbon source, was detected. As for enzymes of the tricarboxylic acid cycle, an increase in abundance of isocitrate dehydrogenase which catalyzes the rate-limiting step of the cycle, was detected (EC 1.1.1.42). Two protein spots related to ATP production were found to be increased in abundance under drought—ATP synthase (EC 3.6.3.14) subunit beta and NADH-ubiquinone oxidoreductase (EC 1.6.5.3). The general down-regulation of carbohydrate degrading glycolytic enzymes could be linked to reduced root biomass accumulation, and could be regarded as a mechanism to accumulate and store sugars for rapid growth in recovery. Similar decrease of glycolysis-related enzymes in roots under drought stress is reported for soybean (Alam et al., 2010). In the roots of other species—the xerophyte wild watermelon, an up-regulation of glycolysis and tricarboxylic acid cycle was found (Yoshimura et al., 2008). Tricarboxylic acid cycle is embedded into a larger metabolic network, constantly sharing substrates and products with other pathways (Sweetlove et al., 2010). Besides carbohydrates, the TCA cycle may be fuelled by products derived from protein and other macromolecules degradation, in order to produce sufficient ATP to meet the energetic needs under stress. The up-regulation of ATP-synthesis related enzymes could be explained by the need of energy for stress protection and maintaining tissue functional state under water limiting conditions. ATP energy is necessary for many cellular processes, including secondary metabolism and protein quality control.

### Dynamic changes are observed in enzymes related to amino acid and one carbon metabolism

We have found increased abundance under drought of some enzymes related to one-carbon and amino acid metabolism—S-adenosyl methionine synthase (EC 2.5.1.6) and formate dehydrogenase (EC 1.2.1.2); glutamine synthetase (EC 6.3.1.2) and cysteine synthase (E.C.2.5.1.47). Decrease in content was detected after prolonged drought for methylene tetrahydrofolate reductase (EC 1.5.1.20), a cytoplasmic enzyme member of one-carbon metabolism. The most interesting qualitative differences were in one cysteine synthase isoform which spot was below detection under drought; however, there was another spot identified also as cysteine synthase with increased abundance under drought. The amino acid cysteine is incorporated into proteins and glutathione (GSH); moreover, it is considered to be the bottleneck for GSH production. The cysteine synthase complex is considered to be the rate-limiting step of cysteine biosynthesis (Chan et al., 2013). Besides, cysteine acts as sulfur donor for methionine (Met) for S-adenosylmethionine and S-methylmethionine synthesis (Ravanel et al., 1998). Glutamine synthetase catalyzes the condensation of glutamate and ammonia to form glutamine, thus playing an essential role in nitrogen metabolism and ammonia assimilation. Ammonia is produced by nitrate reduction or amino acid degradation; on the other hand the amide group of glutamate serves as a readily mobilized nitrogen source for incorporation of amino group in various metabolites. Glutamine synthetase isoforms are reported to be highly responsive to drought (Yoshimura et al., 2008; Alam et al., 2010; Singh and Ghosh, 2013). S-adenosyl methionine synthase catalyzes the conversion, at the expense of ATP, of L-methionine into S-adenosylmethionine—AdoMet or SAM, the major methyl donor for proteins, nucleic acids, carbohydrates, lipids, and small molecules for lignin and many other biosynthesis, precursor for polyamine ant ethylene biosynthesis. Different trends of change in the enzymes related to one-carbon metabolism were reported in drought-stressed roots from different plant species and stress treatment (Yoshimura et al., 2008; Mohammadi et al., 2012; Grebosz et al., 2014). In our study, the accumulation of S-adenosyl methionine synthase and related enzymes in holm oak roots under drought could be linked to enhanced secondary metabolism and lignification which utilize activated one carbon particles.

### Protein synthesis, folding/processing and degradation processes are highly responsive to the applied stress

Changes in the protein complement of cells are indispensable for adaptation to stress. Within the large group of proteins related to protein synthesis, folding/processing and degradation, some spots with opposite trends of changes under stress were found, which may reflect the dynamic changes in cell protein profile. A DEAD box RNA helicase was detected among the identified proteins with earlier and reversible increase in abundance under drought. RNA helicases are involved in various aspects of RNA metabolism, including nuclear transcription, pre mRNA splicing, ribosome biogenesis, nucleocytoplasmic transport, translation, RNA decay, and organellar gene expression (Aubourg et al., 1999). Similar increase of DEAD box RNA helicase was reported in osmotically stressed triticosecale roots (Grebosz et al., 2014). On the other hand, glycyl-tRNA synthetase abundancy was found to decrease under drought and in recovery which is not in favor of up-regulation of translation under drought stress and may rather reflect changes in the composition of newly synthesized proteins. Chaperones and proteases are elements of the protein quality control machinery. Maintaining proteins in their functional conformation, preventing aggregation of non-native proteins, refolding of denatured proteins and removal of non-functional and potentially harmful polypeptides are very important for cell survival under drought stress (Vaseva et al., 2012). Some molecular chaperones, which assist protein folding or renaturation, presented complex changes: chaperonin 60 kD, protein disulfide isomerase and chaperone of TCP-1 family decreased in abundance, while GroES chaperonin increased following water stress. TCP-1 family chaperones are related to cpn60/groEL chaperonin family and assist the folding of cytoskeletal proteins in the cytoplasm (Wang et al., 2004). Cpn60 is homologous to *E. Coli* GroEL found in chloroplasts and mitochondria. It acts in cooperation with GroES chaperonin in assisting proper folding of newly synthesized and membrane-translocated proteins into their native conformation. The observed different abundance changes in GroEL/GroES may reflect changes in composition within the chaperonin family. Under stress the folding capacity of cpn60 is suppressed while its binding affinity toward unfolded proteins is increased, thus protecting proteins from unfavorable conditions by sequestration (Vaseva et al., 2012). Protein disulfide isomerase is an enzyme located in the endoplasmic reticulum with the main function—correct arrangement of disulfide bonds in proteins. Decrease in abundance in this enzyme could reflect inhibition of protein synthesis at the endoplasmic reticulum. The proteasome plays a crucial role in the turnover of regulatory proteins, cellular house-keeping and stress tolerance (Kurepa and Smalle, 2008). Relatively early increase in abundance under stress was found for an ubiquitin activating enzyme E1 which may reflect the importance of stress signaling. The 6B regulatory subunit of 26S proteasome, involved in ATP-dependent protein degradation in the cytosol and nucleus, decreased under drought, while the catalytic proteasome subunit beta type increased. Some evidence suggests that the control of proteasome function may be at the level of subunit composition rather than total increase in proteasome abundance (Kurepa and Smalle, 2008). The proteasome could function in two forms—26S and 20S, the latter containing only the catalytic core subunits and operating without ATP in degradation of oxidized proteins. Thus, 20S may play an important role in tolerance to the secondary oxidative stress developing under drought stress (Vaseva et al., 2012). Up-regulation of the 20S proteasome subunit was found in drought-treated poplar trees (Plomion et al., 2006), alfalfa plants (Aranjuelo et al., 2011), and rapeseed roots (Mohammadi et al., 2012). Opposite changes were observed for some aminopeptidases in roots under applied stress. Leucine aminopeptidase (EC 3.4.11.1) abundancy diminished under drought and in recovery, while Xaa-pro aminopeptidase (EC 3.4.11.9) increased. Aminopeptidases liberate free aminoacids from the N-terminus of the polypeptide chains. Amino acid metabolism was found to be among the top biological processes affected by drought (Kang et al., 2011).

In conclusion, differently abundant identified protein species in holm oak roots subjected to water limitation treatment point at early activation of secondary metabolism, down-regulation of glycolysis and stimulation of ATP synthesis, accumulation of some enzymes related to aminoacid and one-carbon metabolism, and complex changes in protein synthesis, folding/processing and degradation processes, which emphasize the active metabolic adjustment and mobilization of the defense system in roots to actively counteract stress.

### Conflict of interest statement

The reviewer Sabine Lüthje declares that, despite having co-hosted the Research Topic INPPO World Congress 2014 with the author Jesús V. Jorrín-Novo, the review process was handled objectively. The authors declare that the research was conducted in the absence of any commercial or financial relationships that could be construed as a potential conflict of interest.
