# Supplementary material for: 2-DE proteomics analysis of drought treated seedlings of Quercus ilex supports a root active strategy for metabolic adaptation in response to water shortage
Source: Front Plant Sci. 2015 Aug 14;6:627. doi: 10.3389/fpls.2015.00627 (PMC4536546; doi:10.3389/fpls.2015.00627)
Supplement: Supplementary file 1 [file Table1.DOCX]

**Table S1.** Detailed table of identified differentially abundant protein species.

| **SSP** | **Prot.**  **Sco-**  **re** | **Prot.**  **score**  **C.I.%** | **Homologous. protein name** | **Plant species** | **Function/**  **localisation** | **Fold Changes D/R** | **Experim.**  **MW/pI** | **Theoret.**  **MW/pI** | **Accession N** | **Pep.count** | **Total ion score** | **Total ion C.I.%** |
| --- | --- | --- | --- | --- | --- | --- | --- | --- | --- | --- | --- | --- |
| 0005 | 77 | 99.87 | Stem-specific protein TSJT1, RCOM_0913370 | *Ricinus*  *Communis* | Auxin responsive, Al ion induced, chloroplast | D_10_↓ 5x  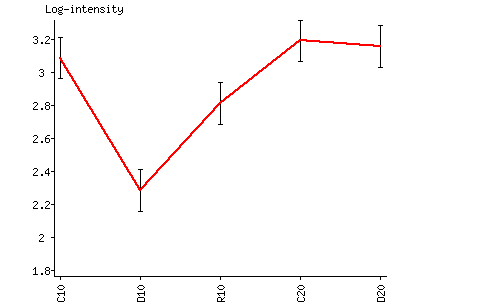 | 20.5/5.54 | 25.5/5.56 | tr\|B9RTW0\|B9RTW | 2 | 70 | 99.998 |
| 0103 | 82 | 99.95 | Uncharacterized protein  Solyc01g094240.2 | *Solanum lycopers.* | short-chain dehydrogenases/reductases (SDR) family | R_10_↓ 3x  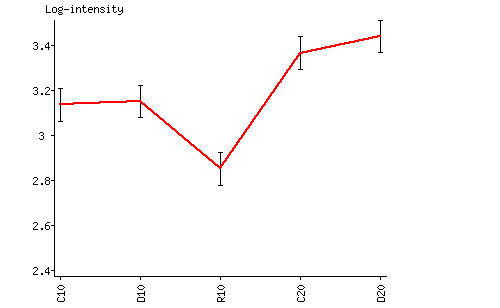 | 25.77/5.4 | 35.46/5.39 | tr\|K4AZG5\|K4AZG | 1 | 78 | 100 |
| 0104 | 83 | 99.97 | (+)-neomenthol dehydrogenase | *Arabid.*  *thaliana* | Monoterpe-noid biosynth.  Defence response | R_10_↓ 2x  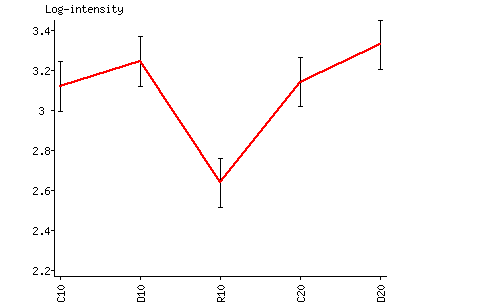 | 25.81/5.46 | 33.07/5.38 | sp\|Q9M2E2\|SDR1_ | 4 | 68 | 99.997 |
| 0105 | 433 | 100 | Caffeoyl CoA 3-O-methyl transferase | *Betula*  *platyphylla* | Aromatic compound metabolism; phenylpropanoid biosynthesis | D_10_↑ 1,7x  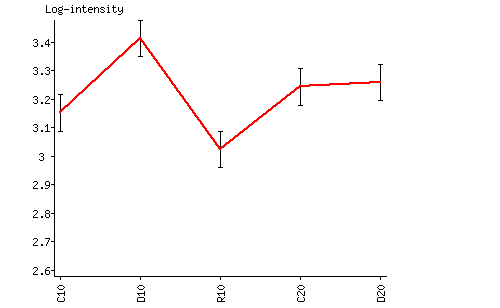 | 23.07/5.48 | 27.95/5.29 | tr\|Q5I2D1\|Q5I2D | 12 | 325 | 100 |
| 0108 | 231 | 100 | Hypersensitive induced response  protein 3 | *Triticum*  *aestivum* | Membrane cation transport cytoskeletal anchor | D_10_↓2,5x  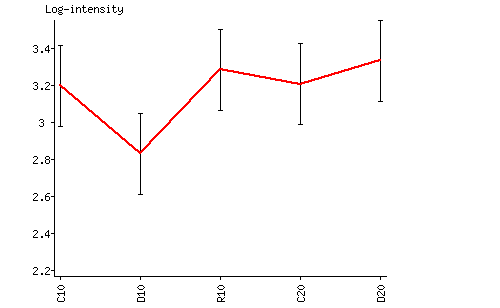 | 24.68/5.67 | 31.8/5.68 | tr\|B6D9L4\|B6D9L | 6 | 202 | 100 |
| 0109 | 423 | 100 | Oxygen-evolving enhancer protein 1, chloroplastic  (Fragments) | *Populus euphratica* | Photosynthesis photosystem II stabilisation, plastid | Q  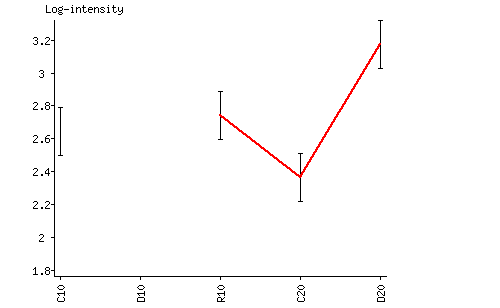 | 24.86/5.48 | 10.6/5.36 | sp\|P84989\|PSBO_ | 6 | 361 | 100 |
| 0113 | 62 | 95.93 | PsbP domain-containing protein | *Medicago*  *truncatula* | Photosynthesisoxygen evolving complex | R_10_↓ 17x  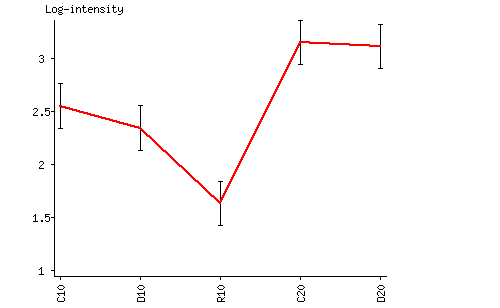 | 26.71/5.3 | 27.95/6.92 | tr\|G7J6G5\|G7J6G | 2 | 55 | 99.963 |
| 0203 | 232 | 100 | Aldo/keto reductase AKR | *Manihot esculenta* | Small molecule biosynthesis | D_10,20_↑  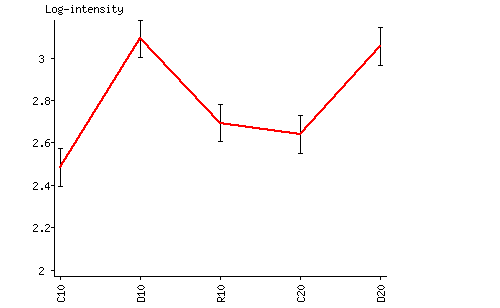 | 35.02/5.63 | 38.03/6.38 | tr\|Q52QX9\|Q52QX | 5 | 210 | 100 |
| 0404 | 787 | 100 | Actin 2 | *Gossypium hirsutum* | Cell motility, Cytoplasm, cytoskeleton | D_10,20_↑  1,8x  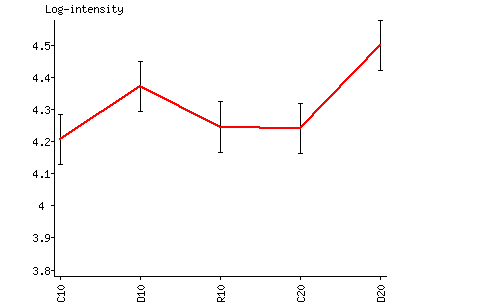 | 45.11/5.68 | 41.94/5.38 | tr\|B8YPL4\|B8YPL | 20 | 597 | 100 |
| 0507 | 77 | 99.865 | Peroxidase | *Linum usitatissimum* | H2O2 metabolism, cytoplasm | D_10_↓D_20_↑3x  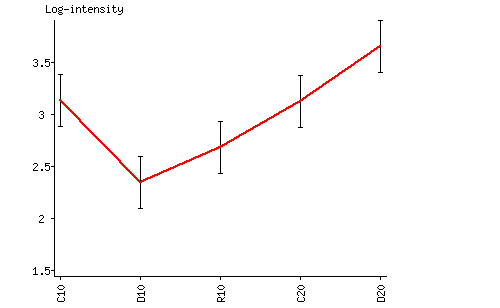 | 48.17/5.44 | 38.63/8.07 | tr\|Q43782\|Q4378 | 3 | 68 | 99.997 |
| 0606 | 227 | 100 | Putative alanine aminotransferase | *Oryza sativa*  *subsp. japonica* | Aminoacid metabilosm | D_10,20_↓  2x  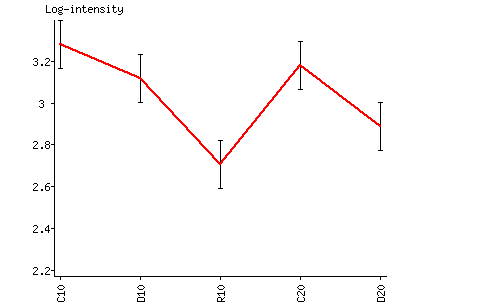 | 56.56/5.5 | 53.77/5.15 | tr\|Q7X7S9\|Q7X7S | 7 | 199 | 100 |
| 0703 | 788 | 100 | Actin 2 | *Annona cherimola* | Cell motility, Cytoplasm, cytoskeleton | Q  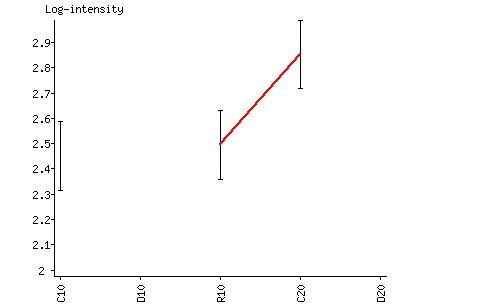 | 65.5/5.51 | 41.85/5.31 | tr\|H9A1W7\|H9A1W | 16 | 652 | 100 |
| 1201 | 251 | 100 | Pyruvate dehydrogenase E1 comp. subunit beta, | *Pisum sativum* | Glycolysis  Mitocondrial matrix | D_20_↑1,5x  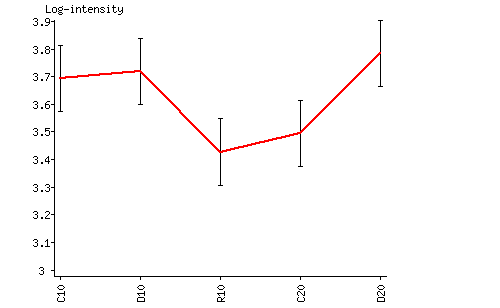 | 38.03/5.75 | 38.99/5.88 | sp\|P52904\|ODPB_ | 4 | 234 | 100 |
| 1202 | 201 | 100 | Protein disulfide-isomerase | *Nicotiana tabacum* | Cell redox homeostasis, EPR protein processing | R↓2,5x  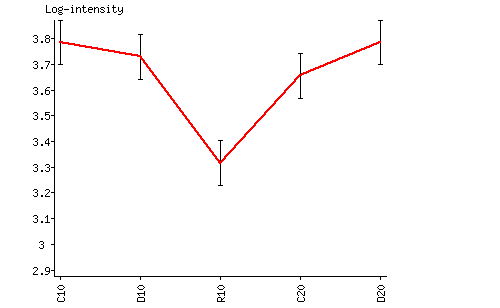 | 38.81/5.83 | 40.08/5.99 | tr\|P93358\|P9335 | 4 | 180 | 100 |
| 1401 | 367 | 100 | Glucose-6-phosphate isomerase | *Populus*  *trichocarpa* | Glycolysis, gluconeogenesiscytoplasm | D_10,20_↑1,5x  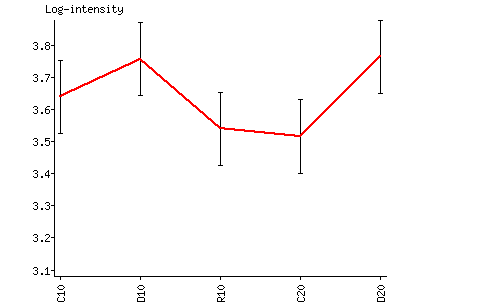 | 45.56/5.8 | 68.1/5.55 | tr\|B9GV29\|B9GV2 | 15 | 275 | 100 |
| 1402 | 540 | 100 | 26S protease regulatory subunit 6B | *Medicago*  *truncatula* | ATP -dep. protein degradation, cytoplasm, nucleus | D_10,20_↓  1,5x  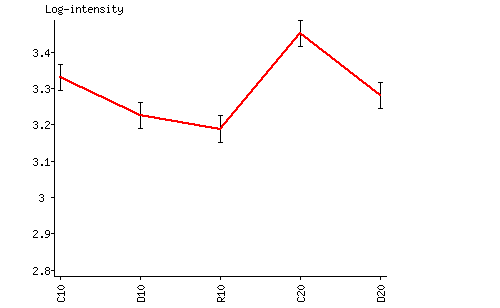 | 51.86/5.85 | 46.58/5.4 | tr\|G7IV48\|G7IV4 | 22 | 335 | 100 |
| 1407 | 574 | 100 | Glucose-1-phosphate adenylyl transferase | *Glycine*  *max* | Gluconeogenesis, glycolysis  cytoplasm | D↑1,5x  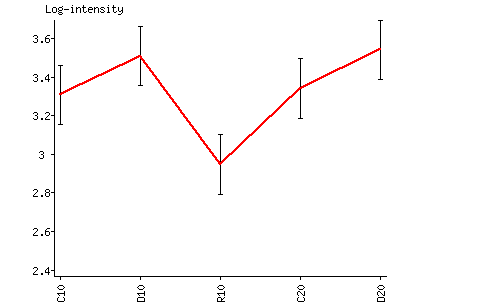 | 51.01/6.03 | 56.71/6.3 | tr\|I1JJP2\|I1JJP | 17 | 445 | 100 |
| 1502 | 350 | 100 | Dihydrolipoyl lysine-residue acetyltransferase | *Arabidopsis thaliana* | component 3 of pyruvate dehydrogenase complex,  mitochondrial | R↓5,5x  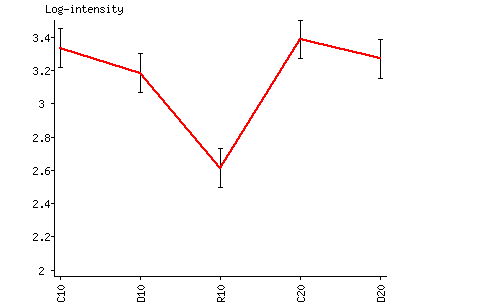 | 54.32/5.84 | 58.89/7.95 | sp\|Q5M729\|OPD23 | 6 | 324 | 100 |
| 1504 | 384 | 100 | Enolase | *Jatropha curcas* | glycolysis | D_10,20_↑1,8x  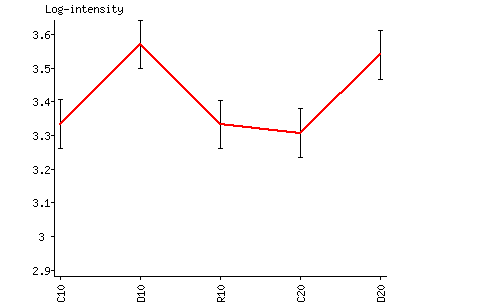 | 55.7/5.92 | 52.69/6.29 | tr\|E6NU46\|E6NU4 | 11 | 319 | 100 |
| 1507 | 187 | 100 | Enolase | *Spinacia oleracea* | glycolysis | R↓ 5x  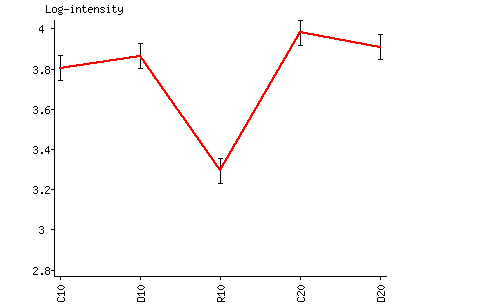 | 55.25/6.01 | 48.37/5.49 | tr\|Q9LEE0\|Q9LEE | 9 | 134 | 100 |
| 1509 | 310 | 100 | DEAD box RNA helicase | *Pisum sativum* | mRNA transport RNA-dep. ATPase | D_10_↑1,8x  R↓2x  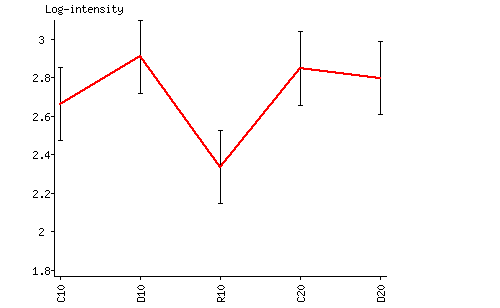 | 50.52/5.55 | 47.14/5.39 | tr\|Q8H1A5\|Q8H1A | 15 | 202 | 100 |
| 1601 | 942 | 100 | ATP synthase subunit beta | *Vitis vinifera* | ATP synthesis coupled H ion transport | D_10,20_↑1,7x  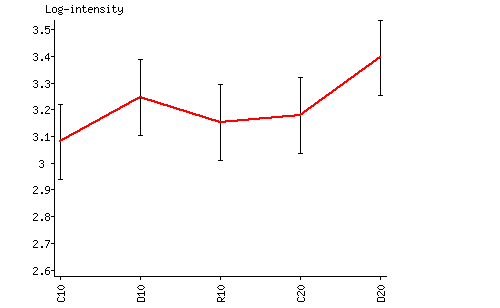 | 58.71/5.74 | 59.3/5.9 | tr\|F6GTT2\|F6GTT | 21 | 761 | 100 |
| 1603 | 246 | 100 | Betaine aldehyde dehydrogenase | *Amaranthus hypo-chondr.* | Amine and polyamine biosynthesis, plastids | D_10_↑2x  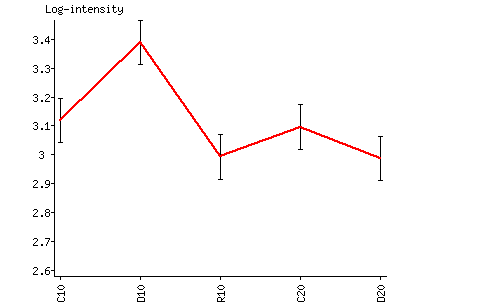 | 63.69/5.84 | 55.4/5.4 | sp\|O04895\|BADH_ | 6 | 222 | 100 |
| 1605 | 152 | 100 | Betaine-aldehyde dehydrogenase | *Corylus*  *heterophylla* | Amine and polyamine biosynthesis, plastids | D_20_↓4,5x  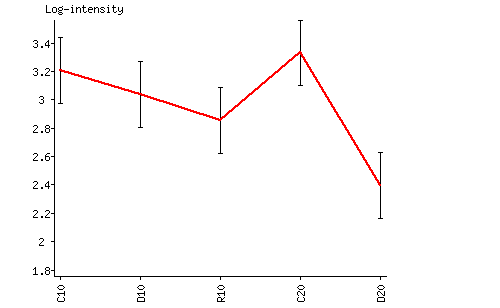 | 63.72/5.96 | 55.2/5.44 | tr\|E9NRZ9\|E9NRZ | 6 | 127 | 100 |
| 1701 | 393 | 100 | Chaperonin-60kD, ch60, putative | *Ricinus*  *communis* | Protein folding, mitochondria | D_10,20_↓1,6x  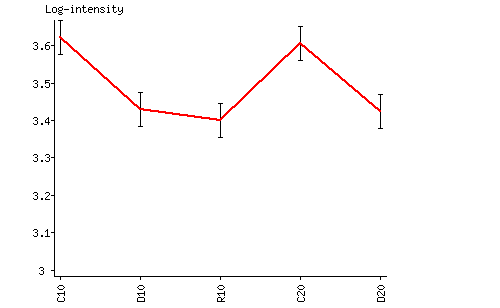 | 66.69/5.73 | 61.48/6.2 | tr\|B9S582\|B9S58 | 13 | 332 | 100 |
| 1702 | 249 | 100 | 2,3-bisphospho glycerate-independent  phosphoglycerate mutase | *Ricinus communis* | Carbohydrate degradation, glycolysis, cytoplasm | D_10,20_↓3x  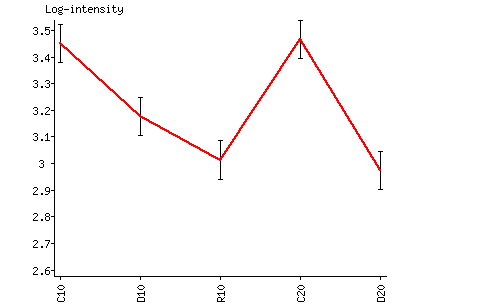 | 70.51/5.89 | 61.01/5.52 | sp\|P35493\|PMGI_ | 8 | 205 | 100 |
| 1901 | 87 | 99.988 | Ubiquitin-activating enzyme E1, putative | *Ricinus*  *communis* | Protein ubiquitination  Cytoplasm, nucleus | D_10,_↑3x, Q  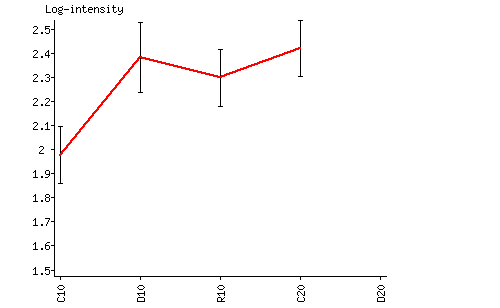 | 117.43/5.8 | 123.59/5.04 | tr\|B9SKZ1\|B9SKZ | 9 | 67 | 99.997 |
| 2004 | 651 | 100 | Proteasome subunit beta type | *Glycine max* | ATP dependent protein degradation | D_10,20_↑1,5x  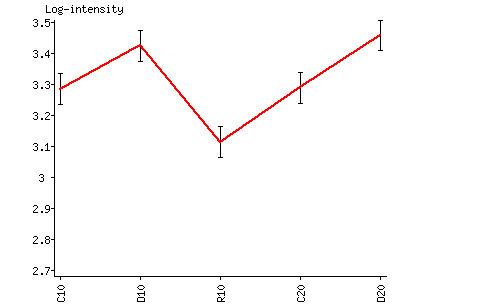 | 18.43/6.22 | 25.17/5.3 | tr\|C6SVE5\|C6SVE | 8 | 592 | 100 |
| 2102 | 409 | 100 | Cysteine synthase | *Ricinus communis* | Aminoacid biosynthesis | D_10,20_↑1,5x  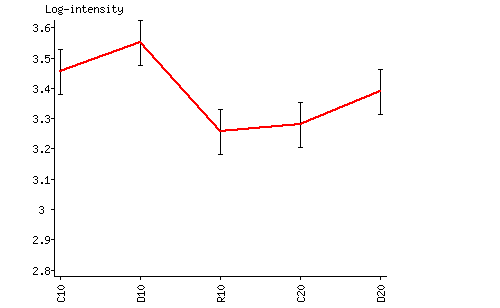 | 31.66/6.15 | 34.44/ 5.5 | tr\|B9RET4\|B9RET | 7 | 369 | 100 |
| 2105 | 94 | 99.997 | Aluminum induced protein with YGL and LRDR motif | *Arabidopsis thaliana* | response to abscisic acid stimulus  cytosol, nucleus | D_10_ ↓ 1,5x  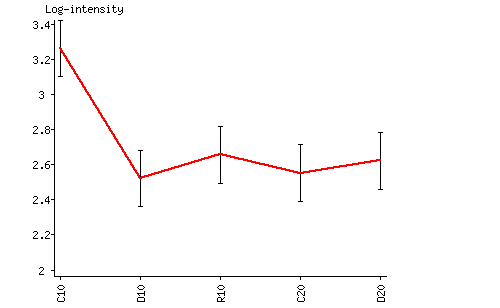 | 22.29/5.96 | 27.5/5.84 | tr\|Q9LIL3\|Q9LIL | 2 | 85 | 100 |
| 2303 | 327 | 100 | Isocitrate dehydrogenase, putative | *Ricinus*  *communis* | Tricarboxylic acid cycle  Cytosol, plastids | D↑1,6x  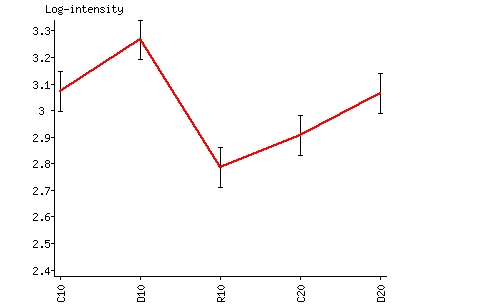 | 41.8/6.11 | 40.63/7.04 | tr\|B9SRZ2\|B9SRZ | 10 | 258 | 100 |
| 2402 | 555 | 100 | S-adenosylmethionine synthase | *Camellia sinensis* | Amino acid  and lignin biocynthesis  one carbon metabolism cytoplasm | D_10_↑D_20_↓  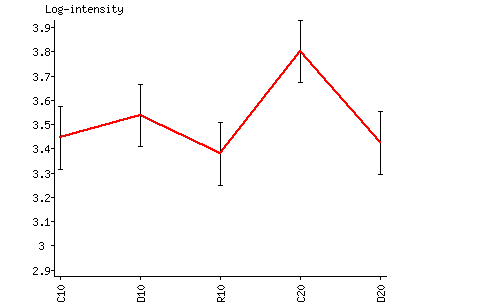 | 48.07/6.06 | 43.23/5.34 | sp\|Q9LDQ7\|METK_ | 17 | 403 | 100 |
| 2405 | 731 | 100 | S-adenosylmethionine synthase | *Elaeagnus*  *umbellata* | Amino acid  and lignin biocynthesis  one carbon metabolism cytoplasm | D_10_↑D_20_↓  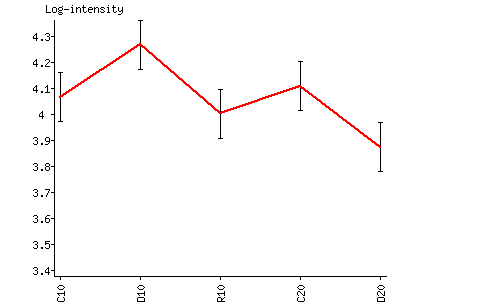 | 47.84/6.17 | 43.56/5.5 | sp\|Q9AT55\|METK | 18 | 563 | 100 |
| 2603 | 292 | 100 | Leucine aminopeptidase  MTR_4g130860 | *Medicago truncatula* | Proteolysis cytoplasm  Metalloexopeptidase (Mg ion binding) | D_10,20_↓2,5x  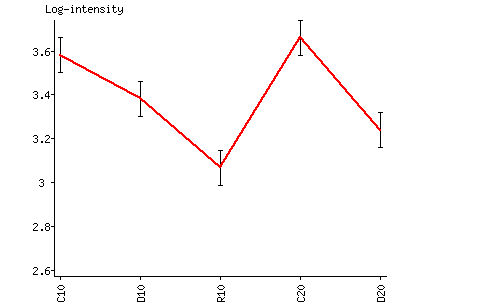 | 58.61/6.1 | 59.86/7.56 | tr\|G7JGP9\|G7JGP | 12 | 220 | 100 |
| 2701 | 306 | 100 | 2,3-bisphosphoglycerate-independent  Phosphoglyce-rate mutase  RCOM_1323170 | *Ricinus communis* | Glucose catabolism, stress response | D_10,20_↓ 2x  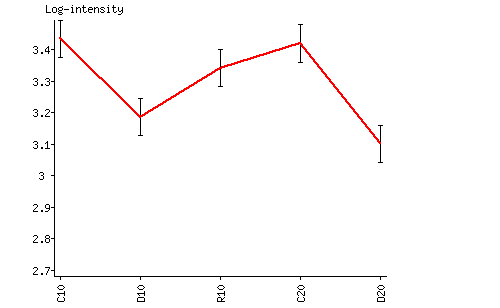 | 69.86/6.08 | 61.38/5.52 | tr\|B9S1V6\|B9S1V | 9 | 255 | 100 |
| 3007 | 86 | 99.983 | Groes chaperonin, putative  RCOM_0710470 | *Ricinus communis* | Chaperone, protein folding, ATP binding, cytoplasm | D_10,20_↑2,3x  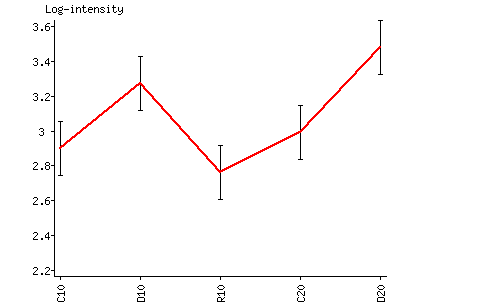 | 19.06/6.31 | 26.58/8.89 | tr\|B9RR63\|B9RR6 | 5 | 53 | 99.912 |
| 3205 | 325 | 100 | Aldo/keto reductase AKR | *Manihot esculenta* | Oxidation/reduction process, small molec. biosynthesis, oxidative stress | D_10,20_↑ 2x  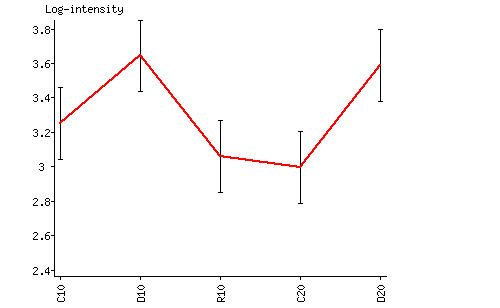 | 38.15/6.34 | 38.03/6.38 | tr\|Q52QX9\|Q52QX | 6 | 296 | 100 |
| 3206 | 75 | 99.786 | Cysteine synthase  At3g59760 | *Arabi-dopsis thaliana* | Amino acid biosynthesis cytoplasm | Q  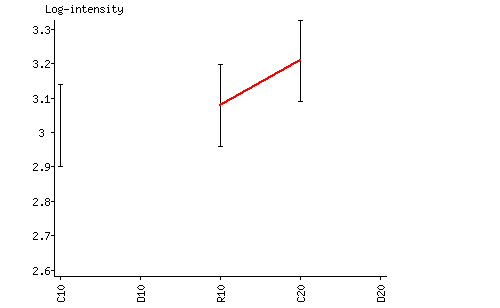 | 35.25/6.49 | 46.13/8.39 | tr\|Q0WWQ5\|Q0W  WQ | 9 | 27 | 67.421 |
| 3302 | 206 | 100 | Glutamine synthetase  CsGS1 | *Camellia sinensis* | Ammonia reassimilation plastids, mitochondria | D_10,20_↑1.5x  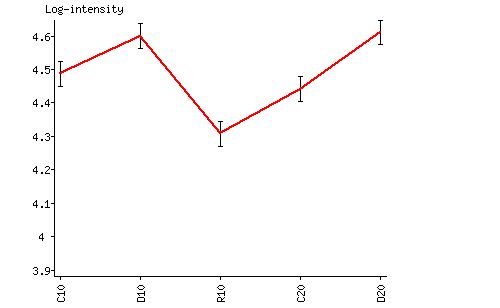 | 40.11/6.28 | 39.41/5.52 | tr\|Q762D2\|Q762D | 4 | 188 | 100 |
| 3303 | 840 | 100 | Flavanone 3-hydroxylase  F3H | *Ampelopsis*  *grossedentata* | Biosynthesis of flavones, anthocyanidins, hormones | D_10_↑ 2x  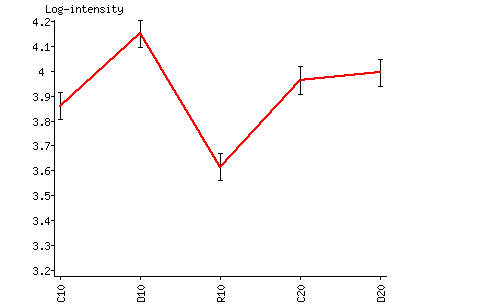 | 40.46/6.35 | 41.2/5.33 | tr\|I6ZTY9\|I6ZTY | 17 | 686 | 100 |
| 3503 | 460 | 100 | Enolase PGH1 | *Alnus glutinosa* | glycolysis | D_20_↓ 2x  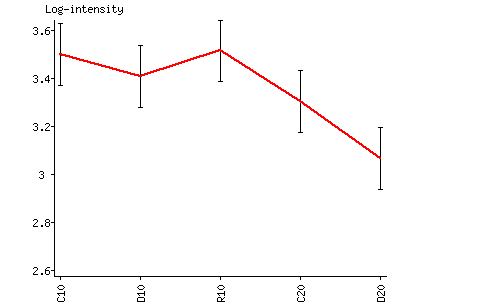 | 56.84/6.35 | 47.79/5.41 | sp\|Q43321\|ENO_A | 10 | 396 | 100 |
| 3604 | 292 | 100 | TCP domain class transcription factor  TCP3 | *Malus*  *domestica* | Chaperonin,TCP-1/cpn60 chaperonin family protein  ATP binding, protein folding  cytoplasm, anchored to plasma membrane | D_10_↓ 2,5x  D_20_↑1,5x  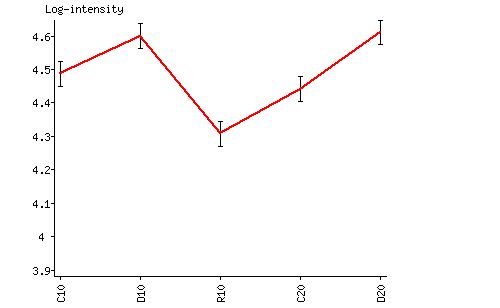 | 63.5/6.33 | 57.4/5.6 | tr\|D9ZJD1\|D9ZJD | 12 | 223 | 100 |
| 3607 | 656 | 100 | Mitochondrial processing peptidase beta subunit | *Cucumis melo* | Proteolysis, peptidase M16 family | D_10,20_↑ R↓  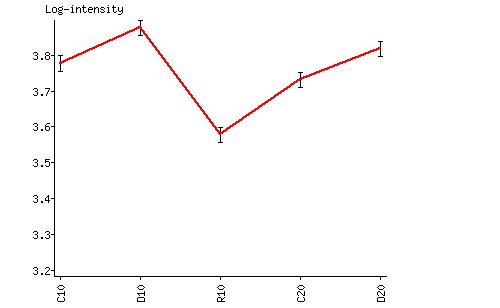 | 62.57/6.45 | 58.9/6.56 | tr\|Q9AXQ2\|Q9AXQ | 13 | 575 | 100 |
| 3703 | 244 | 100 | Pyruvate decarboxylase PDC | *Prunus armeniaca* | ethanol fermentation, anaerobic metabolism, acetaldehyde biosynthesis | D_10_↓ 2,8x  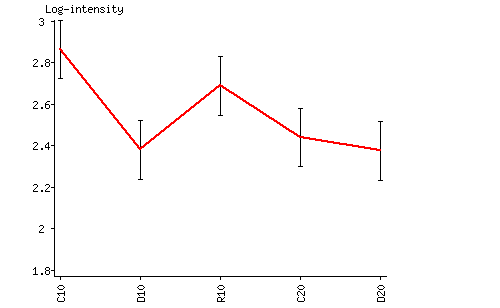 | 69.93/6.25 | 66.2/5.74 | tr\|B0ZS79\|B0ZS7 | 4 | 234 | 100 |
| 3705 | 248 | 100 | Pyruvate decarboxylase PDC | *Prunus armeniaca* | ethanol fermentation, anaerobic metabolism, acetaldehyde biosynthesis | D10↓5x, D_20_ -Q  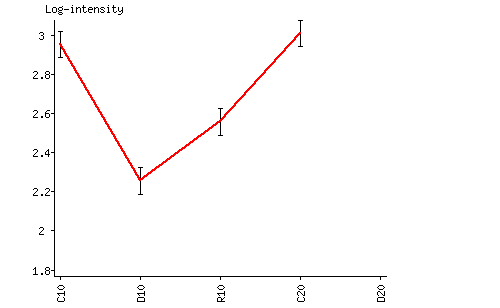 | 70.07/6.36 | 66.2/5.74 | tr\|B0ZS79\|B0ZS7 | 6 | 230 | 100 |
| 3706 | 197 | 100 | Ribulose bisphosphate carboxylase large chain  rbcL | *Noronhia emarginata* | Calvin cycle  CO2 fixation, photorespi-ration, plastids | D_10_↓2,8x, D_20_ -Q  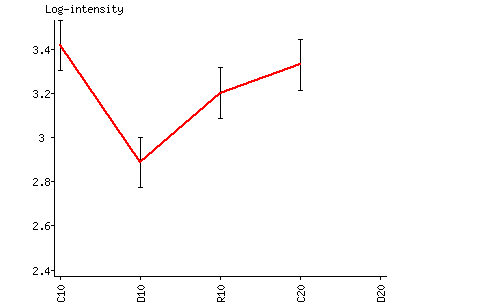 | 66.65/6.42 | 53.05/6.22 | tr\|Q06QN8\|Q06QN | 14 | 101 | 100 |
| 3707 | 240 | 100 | Phosphoglucomutase, cytoplasmic  PGM1 | *Populus*  *tremula* | Carbohydrate metabolism | Q-only R_10_  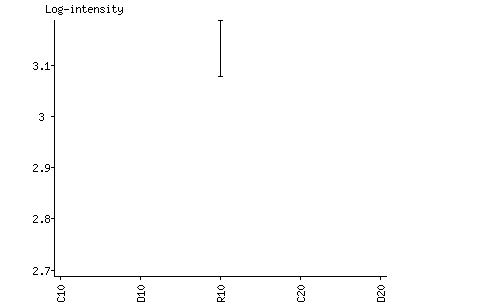 | 74.14/6.42 | 63.37/5.49 | sp\|Q9ZSQ4\|PGMC_ | 15 | 145 | 100 |
| 3803 | 88 | 99.99 | Uncharacterized protein  app1 Xaa-Pro aminopeptidase 1 | *Solanum lycopersicum* | Peptidase M24B family  aminopeptidase | D_10,20_↑1.5x  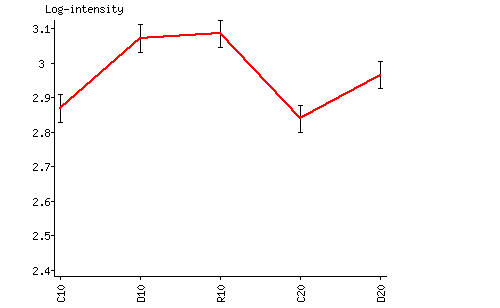 | 79.52/6.42 | 73.45/5.98 | tr\|Q93X46\|Q93X4 | 3 | 81 | 100 |
| 3805 | 443 | 100 | Glycyl-tRNA synthetase  ARALYDRAFT_473197 | *Arabidopsis lyrata subsp.*  *lyrata* | Aminoacyl/tRNA synthetase, translation, cytoplasm | D_10_↓2,4x,  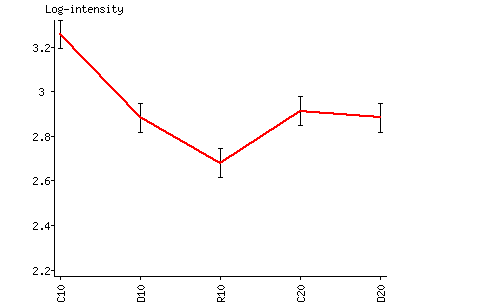 | 78.97/6.46 | 82.49/6.43 | tr\|D7KEM8\|D7KEM | 12 | 395 | 100 |
| 3806 | 235 | 100 | NADH-ubiquinone oxidoreductase 75 kDa subunit | *Zea mays* | Plastids, Mitochondrial respiratory chain, ATP synthesis coupled ET | D_10_↑D_20_↓  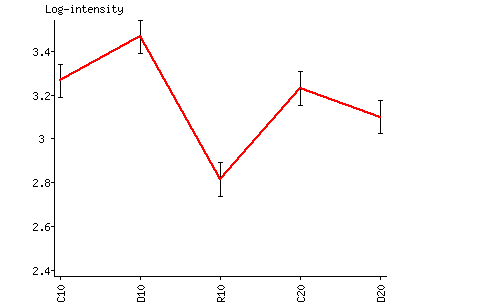 | 84.5/6.42 | 81.67/6.1 | tr\|B6U2J0\|B6U2J | 8 | 212 | 100 |
| 4201 | 327 | 100 | Aldo/keto reductase | *Manihot esculenta* | Oxidoreductase, auxin mediated signaling pathway | D_10_↓ 3x  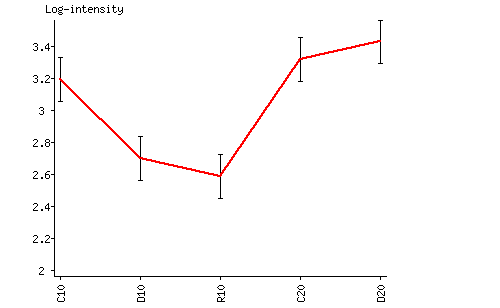 | 38.37/6.51 | 38.03/6.38 | tr\|Q52QX9\|Q52QX | 6 | 296 | 100 |
| 4507 | 69 | 99.224 | UDP-glucosyltransferase, putative  RCOM_0865380 | *Ricinus*  *communis* | Secondary metabolism | D_10_-Q  D_20_↓ 2,6x  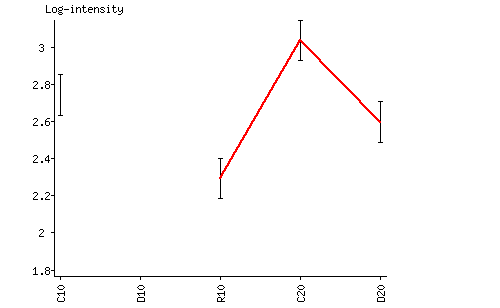 | 54.8/6.61 | 52.97/6.21 | tr\|B9S1I8\|B9S1I | 3 | 59 | 99.987 |
| 4605 | 102 | 100 | Predicted protein (Fragment)  POPTRDRAFT_863097 | *Populus trichocarpa* | Methylmalo-nate-semialdehyde dehydrogenase (acylating) activity | D_20_↓ 4,4x  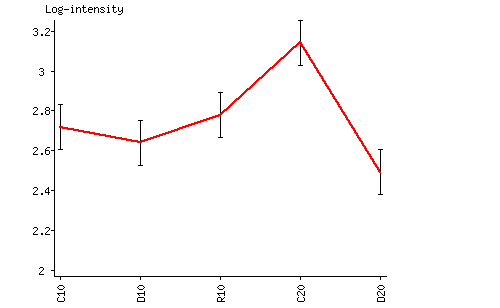 | 59.85/6.68 | 53.87/5.67 | tr\|B9H6P5\|B9H6P 1 102 100 | 1 | 102 | 100 |
| 4702 | 210 | 100 | D-3-phospho glycerate dehydrogenase putative  RCOM_0811570 | *Ricinus communis* | D-isomer specific 2-hydroxyacid dehydrogenase family L-serine biosynthetic process | D_10_↓ 2x  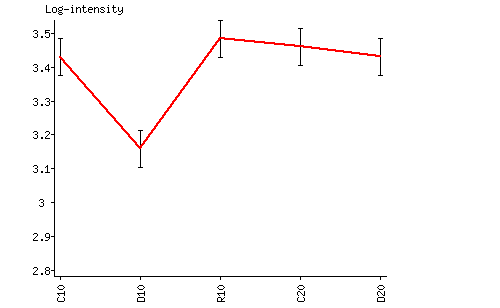 | 64.92/6.54 | 63.35/7.64 | tr\|B9RYA3\|B9RYA | 10 | 162 | 100 |
| 4704 | 104 | 100 | Methylenetetrahydrofolate reductase 2  MTHFR2 | *Arabidopsis*  *thaliana* | One carbon metabolism  cytoplasm | D_20_↓ 3,6x  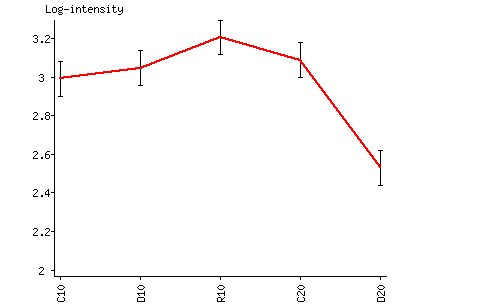 | 65.2/6.62 | 67.39/5.33 | sp\|O80585\|MTHR | 3 | 95 | 100 |
| 4705 | 362 | 100 | Chaperonin containing t-complex protein 1, zeta  subunit, tcpz, RCOM_1727810 | *Ricinus communis* | Chaperone, TCP-1 family, Protein folding, cytoplasm | D_20_↓ 1,5x  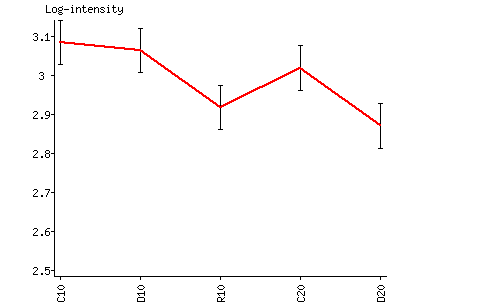 | 70.46/6.64 | 59.56/ 6.08 | tr\|B9RSN1\|B9RSN | 18 | 238 | 100 |
| 4707 | 163 | 100 | Pyrophosphate-dependent phosphofructokinase beta  Sub. PPi-PFKb | *Citrus paradisi* | glycolysis | D_10,20_↓1,5x  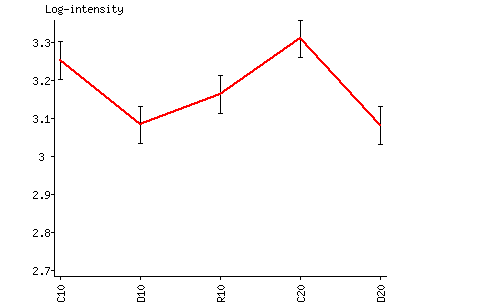 | 65.7/6.71 | 62.23/6.11 | tr\|Q9ZST3\|Q9ZST | 8 | 129 | 100 |
| 4809 | 67 | 98.652 | Uncharacterized protein | *Glycine max* | NCBI blastp - heat shock protein STI-like [Glycine max] | D_10_R_10_↓  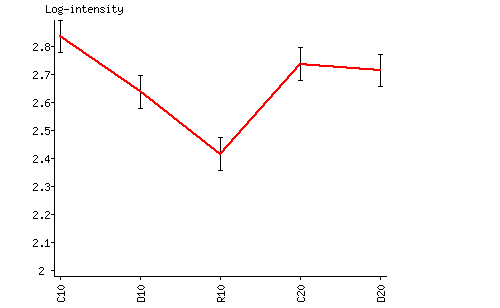 | 77.99/6.58 | 65.86/6.26 | tr\|I1LGM2\|I1LGM | 6 | 54 | 99.95 |
| 4810 | 68 | 99.023 | Uncharacterized protein | *Glycine max* | NCBI blastp - heat shock protein STI-like [Glycine max] | D_10_R_10_↓  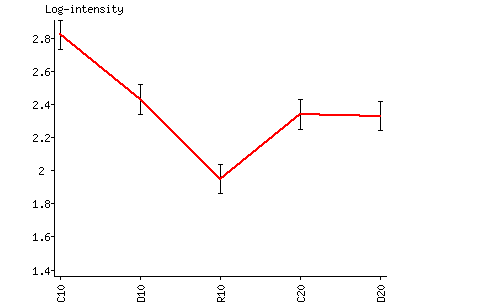 | 78.27/6.63 | 66.91/6.07 | tr\|I1J9X8\|I1J9X | 7 | 50 | 99.854 |
| 5004 | 58 | 90.457 | Proteasome subunit beta type  RCOM_1478370 | *Ricinus communis* | ATP dep. protein degradation, cytoplasm, nucleus | D_10_↑1,6x  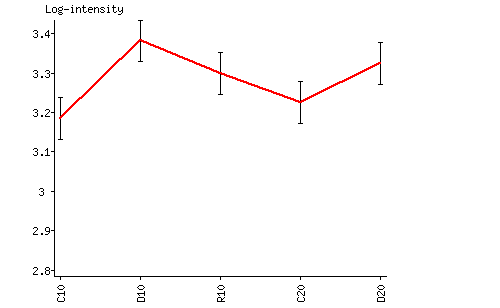 | 20.5/6.92 | 30.6/7.6 | tr\|B9SJ80\|B9SJ8 | 3 | 47 | 99.745 |
| 5106 | 378 | 100 | Triosephosphate isomerase | *Solanum tuberosum* | glycolysis | D_10_R_10_↓  D_20_-Q  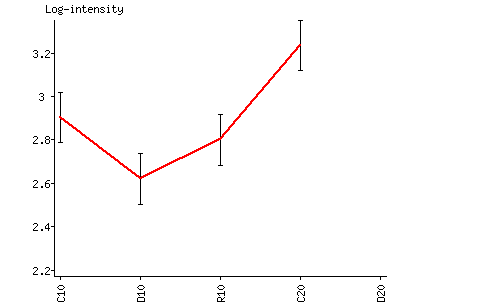 | 20.82/6.81 | 27.92/5.9 | tr\|Q3HRV9\|Q3HRV | 8 | 321 | 100 |
| 5107 | 115 | 100 | Gamma carbonic anhydrase 1, mitochondrial  GAMMACA1 | *Arabidopsis thaliana* | Respiration, mitochondrial membrane peripheral | D_10,20_↑2x  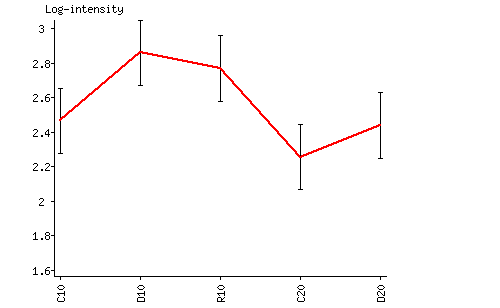 | 23.46/6.91 | 30.12/7.13 | sp\|Q9FWR5\|GCA1_ | 4 | 94 | 100 |
| 5505 | 261 | 100 | UDP-sulfoquinovose synthase, putative  RCOM_0797290 | *Ricinus*  *communis* | Sulfolipid biosynthesis | D_10,20_↓2x  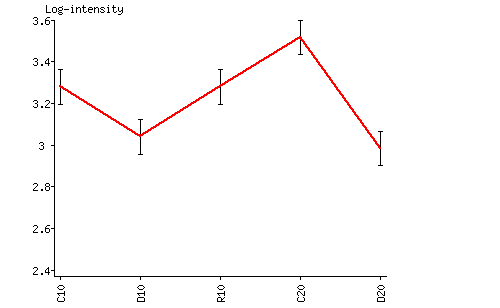 | 52.85/6.89 | 54.28/8.53 | tr\|B9RRR0\|B9RRR | 8 | 222 | 100 |
| 5602 | 461 | 100 | Enolase | *Ricinus communis* | glycolysis | D_10,20_↓4x  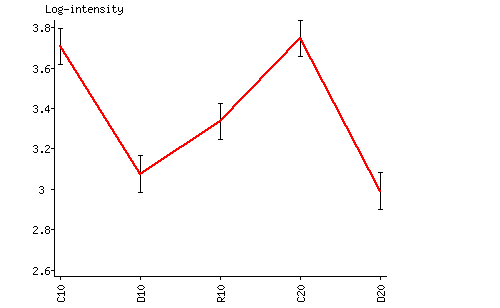 | 58.52/6.78 | 48.15/5.71 | tr\|B9R9N6\|B9R9N | 10 | 400 | 100 |
| 5701 | 157 | 100 | Chaperonin containing t-complex protein 1, gamma  subunit, tcpg,  RCOM _ 0573520 | *Ricinus communis* | Chaperone, TCP-1 family, Protein folding, cytoplasm | D_10,20_↓2x  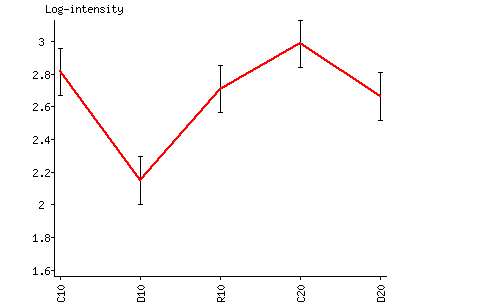 | 69.96/6.78 | 60.64/5.97 | tr\|B9SUJ3\|B9SUJ | 7 | 135 | 100 |
| 5702 | 114 | 100 | Predicted protein  POPTRDRAFT_554969 | *Populus trichocarpa* | NCBI blastp - sorting and assembly machinery (sam50) protein, putative [Ricinus communis] | Q – only R  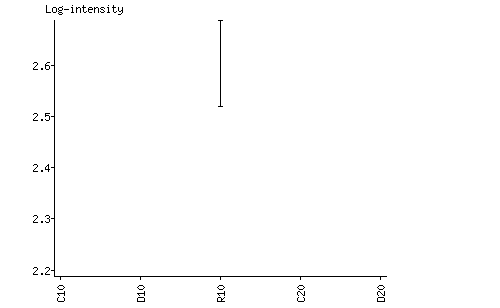 | 73.75/6.79 | 74.62/8.05 | tr\|B9GWA2\|B9GWA | 5 | 104 | 100 |
| 5806 | 166 | 100 | Aconitate hydratase, cytoplasmic | *Cucurbita*  *maxima* | Carbohydrate metabolism; tricarboxylic acid cycle | D_10,20_↓2x  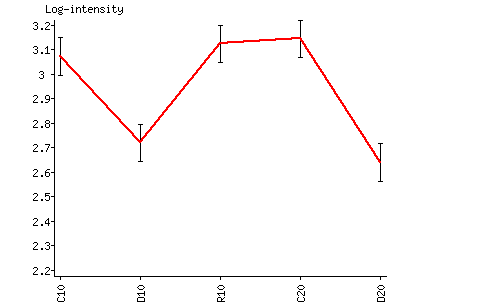 | 98.54/6.89 | 98.57/5.74 | sp\|P49608\|ACOC_ | 12 | 118 | 100 |
| 6302 | 611 | 100 | Alcohol dehydrogenase  Adh1-1a | *Pyrus communis* | Stress response cytosol, plasma membrane | D_10,20_↓8x  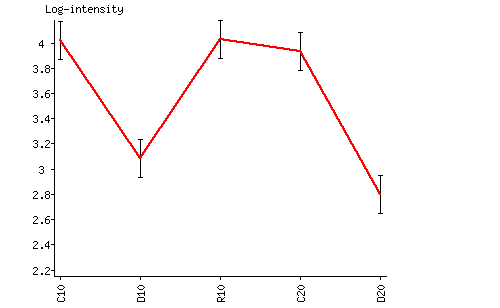 | 41.76/6.96 | 42.08/6.51 | tr\|G0Z9K2\|G0Z9K | 10 | 545 | 100 |
| 6304 | 92 | 99.996 | Dihydroflavonol 4-reductase | *Paeonia suffruticosa* | Secondary metabolism | D_10,20_↑ 2x  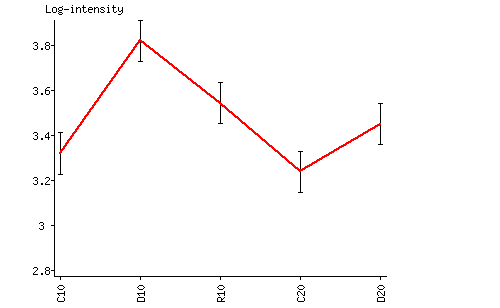 | 41.63/7.03 | 41.398/5.67 | tr\|G4WCQ6\|G4WCQ | 4 | 76 | 100 |
| 6403 | 323 | 100 | Naringenin-chalcone synthase  CHS1 | *Juglans nigra x*  *Juglans regia* | Secondary metabolism, flavonoid biosynthesis | D_10_↑2,6x  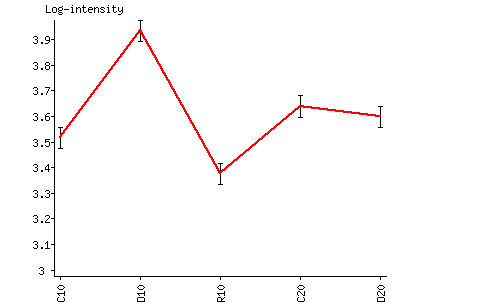 | 46.52/7.05 | 42.9/6.34 | tr\|Q42864\|Q4286 | 12 | 235 | 100 |
| 6601 | 96 | 99.998 | Dehydroquinate dehydratase/ shikimate dehydrogenase  DHQD4 | *Populus trichocarpa* | Aromatic amino acid biosynthesis  Secondaary metabolism | D_10_↑2,2x  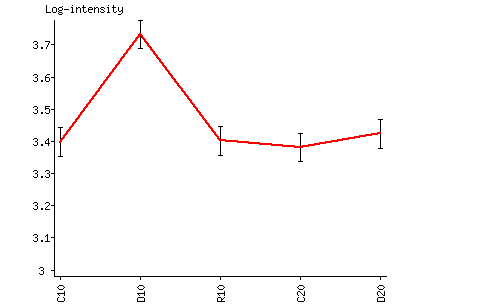 | 64.05/6.94 | 58.27/5.94 | tr\|B9HSF2\|B9HSF | 5 | 80 | 100 |
| 6602 | 77 | 99.868 | Aldehyde dehydrogenase ALDH2B4_V2 | *Vitis*  *vinifera* | Aldehyde metabolism, stress response | D_10,20_↓1,5x  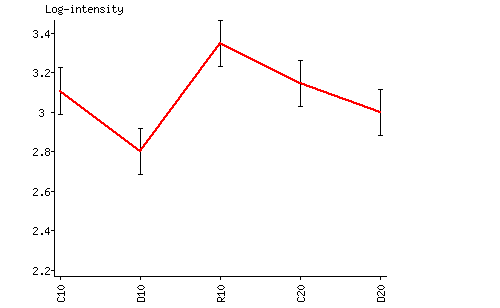 | 60.16/6.98 | 52.53/6.18 | tr\|G9HQG0\|G9HQG | 5 | 63 | 99.993 |
| 6801 | 566 | 100 | Vitamin-b12 independent methionine synthase | *Populus trichocarpa* | Aminoacid biosynthesis, one carbon metabolism,  cytoplasm | D_10,20_↑2x  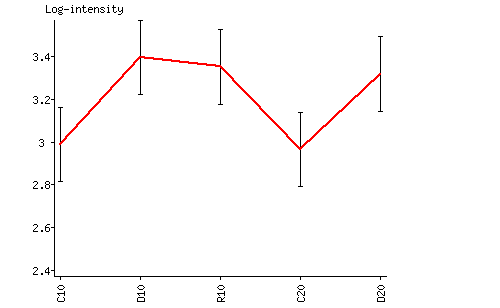 | 87.02/6.97 | 85.03/6.17 | tr\|B9HQI3\|B9HQI | 13 | 499 | 100 |
| 7002 | 224 | 100 | Proteasome subunit beta type | *Picea sitchensis* | ATP dep. protein degradation, cytoplasm, nucleus | D_10_R↑1,5x  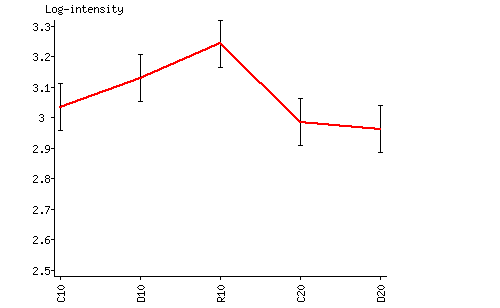 | 19.97/7.36 | 29.59/5.58 | tr\|A9NZ27\|A9NZ2 | 4 | 203 | 100 |
| 7302 | 509 | 100 | Formate dehydrogenase Oxidore-ductase activity, acting on the CH-OH group of donors, NAD or NADP as acceptor | *Quercus robur* | Formate metabolism formate-dependent oxygen uptake coupled to ATP synthesis  mitochindrion | D_10_R↑1,5x  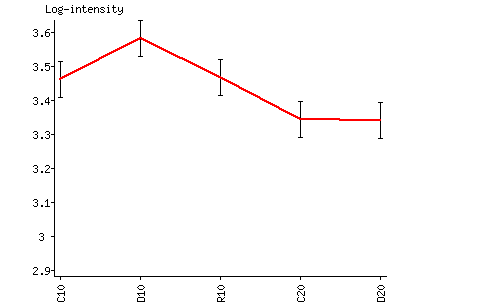 | 43.52/7.13 | 40.85/6.54 | tr\|Q7XHJ0\|Q7XHJ | 17 | 365 | 100 |
| 7306 | 104 | 100 | Fructose-bisphosphate aldolase, cytoplasmic isozyme 2 | *Pisum sativum* | Glycolysis  cytoplasm | Q only R  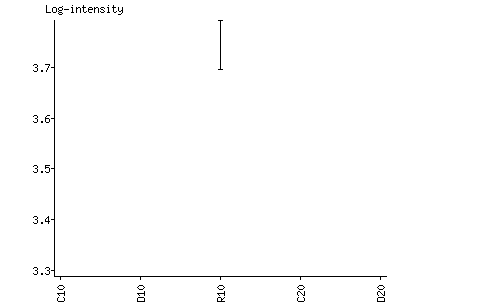 | 43.39/7.43 | 38.64/6.77 | sp\|P46257\|ALF2_ | 5 | 83 | 100 |
| 7401 | 494 | 100 | Chalcone synthase | *Malus domestica* | Secondary metabolism, flavonoid biosynthesis | D_10_↑2,5x  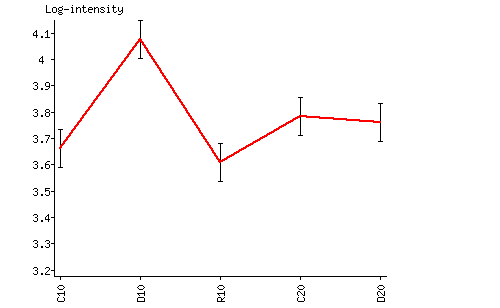 | 46.7/7.12 | 42.79/5.65 | tr\|B8R6A2\|B8R6A | 15 | 366 | 100 |
| 7402 | 338 | 100 | Glutamate dehydrogenase  POPTRDRAFT_826140 | *Populus trichocarpa* | Amino acid metabolism, mitochondrion | D_10,20_↑1,5x  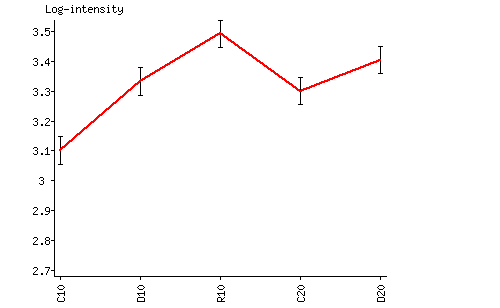 | 45.2/7.15 | 44.72/6.84 | tr\|B9IPQ2\|B9IPQ | 10 | 281 | 100 |
| 7408 | 489 | 100 | Formate dehydro-genase  mitochondria | *Quercus robur* | formate-dependent oxygen uptake coupled to ATP synthesis | D_10,20_↑1,5x  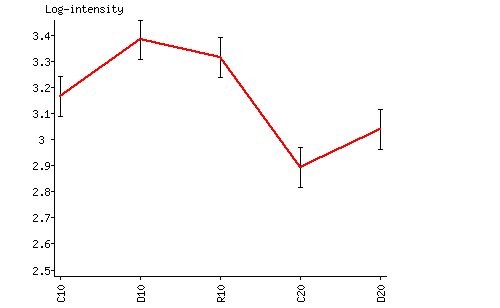 | 44.88/7.37 | 40.85/6.54 | tr\|Q7XHJ0\|Q7XHJ | 15 | 368 | 100 |
| 7605 | 317 | 100 | 6-phospho-fructokinase 3  PFK3 | *Arabidopsis thaliana* | glycolysis | D_10,20_↓1,5x  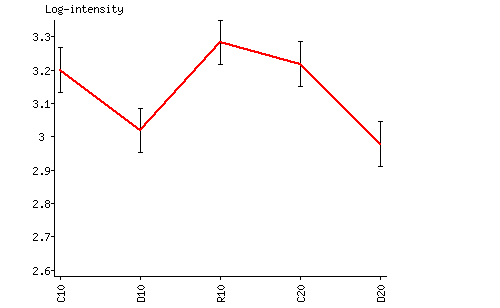 | 61.41/7.4 | 54.09/6.61 | sp\|Q94AA4\|K6PF3 | 10 | 268 | 100 |
| 7701 | 123 | 100 | Malic enzyme  OB01G39450 | *Oryza brachyantha* | Glycolysis, malate metabolism | Q-D absent  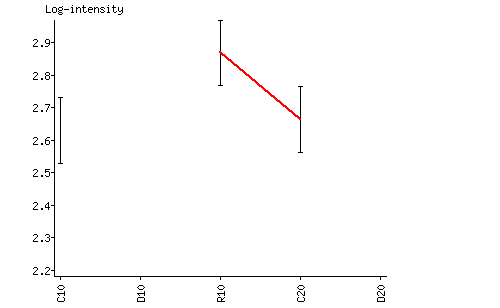 | 76.28/7.11 | 71.65/8.78 | tr\|J3L3Y9\|J3L3Y | 4 | 116 | 100 |
| 8202 | 200 | 100 | Putative uncharacterized protein  SELMODRAFT_107855 | *Selaginella*  *moellendorffii* | NCBI blastp –  quinone oxidoreductase [Arabidopsis thaliana] | D_10_↑D_20_↓  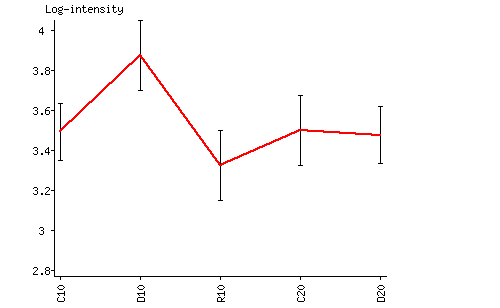 | 36.34/7.58 | 34.88/7.04 | tr\|D8S344\|D8S34 | 4 | 184 | 100 |
|  |  |  |  |  |  |  |  |  |  |  |  |  |

Protein identification – *changingin abundance* protein species in roots ↓diminishing, ↑increasing. D-drought, R-recovery, Q-qualitative changes
