# Supplementary material for: 2-DE proteomics analysis of drought treated seedlings of Quercus ilex supports a root active strategy for metabolic adaptation in response to water shortage
Source: Front Plant Sci. 2015 Aug 14;6:627. doi: 10.3389/fpls.2015.00627 (PMC4536546; doi:10.3389/fpls.2015.00627)
Supplement: Supplementary file 2 [file Table2.DOCX]

Table S2. Localization prediction of identified protein species. Abbreviations: CYT – cytosolic, CYSK – cytoskeleton, NUCL – nuclear, CHL-chloroplast/amyloplast, MIT-mitochondia, PER-peroxisomes, ER, ENDO-endoplasmatic reticulum, VAC-vacuole, SECR- secretory pathway, EXTR – extracellular, PLAS – plasma membrane, OTHER – nonCHL/MIT/SECR for TargetP and PredSL.

| SSP | Homologous protein | Mascot data | | WolfPSORT | | TargetP | | PredSL | | MultiLoc | | Consensus |
| --- | --- | --- | --- | --- | --- | --- | --- | --- | --- | --- | --- | --- |
|  | Name/ species | Seq. cove  rage | Amino acid sequence of the selected homologue | Locali-  zation | Sco  re | Locali-  zation | Sco  re | Locali-  zation | Sco  re | Locali-  zation | Sco  re |  |
| 0005 | Stem-specific protein TSJT1, RCOM_0913370  *Ricinus Communis* | 33% | MESASENKDT SAELQRKNLG RDIVDDKAAE RKWSDLPLDI LPMIAGRLGI IDLISFRSVC KDWKSASATA SAEIESSPLR **EPWFLLYGGE ASQCLLLSRT GNKYTINIPE MNGATCIASK** K**GWLLLLREE DYSIYFFCPF SR**AKIDLPKL QNLASVPNHL AAFPSAPTSQ DCFVSIVCR**D ETSSPEMIQL YVLLRGDK**EW TKHTYSHTSV GELDKIKCVA YYDDELHIMD AR**TQILITYS FKTSMWK**KHT ILHYPVPHAP FLKFCMASSY FQERDLR**EKL DLEGDVSVTI CGSAFK**IGTK NLDRVIFSES IDATGGSVTR RVKGVWIHPR FFILPPNQSW | CYT  NUCL | 10  3 | CHL MIT  SECR  OTHER | 0.05 0.10 0.04  0.95 | CHL  MIT  SECR  OTHER | 0  0.02  0.01  0.96 | CYT  CHL | 0.39  0.37 | CYT |
| 0103 | Uncharacterized protein Solyc 01 g094240.2  *Solanum lycopers.* | 5% | MAEKITSVDS IRYAVVSGGN KGLGYETCKQ LASKGVVVVL TSRDEKRGIE AIERLKKESN FTNDQVLFHQ LDIMDPASIS SLVDFINTKF GRLDILINNA GIGGLMVEGD VLVLKEIIEG DLFTVSTENG EDGGTMKSYP EITTNYELTK QCVETNYYGA KRTIEAFAPL LQLSNSPRIV NVASFLGKLK LLRNEWAKKV LSDAKSLTED KVEQVLNEFL KDFTENAIES KGWPTNFTAY RVSKAALIAY TRILATKYPN FRVNSVCPGY CKTDVNANTG NLTAEEGAES FVK**LALLPND GPSGLFFYR**Q EVTLF | CYT  CHL | 9  4 | CHL MIT  SECR  OTHER | 0.10 0.12 0.12  0.79 | CHL  MIT  SECR  OTHER | 0  0.01  0.02  0.97 | CYT  PER  CHL | 0.87  0.07  0.02 | CYT |
| 0104 | (+)-neomenthol dehydrogenase  *Arabid.thaliana* | 13% | MAEETPR**YAV VTGANRGIGF EICR**QLASEG IRVVLTSRDE NRGLEAVETL KKELEISDQS LLFHQLDVAD PASITSLAEF VKTQFGKLDI LVNNAGIGGI ITDAEALRAG AGKEGFKWDE IITETYELTE ECIKINYYGP KR**MCEAFIPL LK**LSDSPRIV NVSSSMGQLK NVLNEWAK**GI LSDAENLTEE R**IDQVINQLL NDFKEGTVKE KNWAKFMSAY VVSKASLNGY TRVLAKKHPE FRVNAVCPGF VKTDMNFKTG VLSVEEGASS PVRLALLPHQ ETPSGCFFSR KQVSEF | CYT  CHL | 10  3 | CHL  MIT  SECR  OTHER | 0.1  0.2  0.05  0.67 | CHL  MIT  SECR  OTHER | 0  0.02  0.03  0.95 | CYT | 0.94 | CYT |
| 0105 | Caffeoyl CoA 3-O-methyl transferase  *Betula Platyphylla* | 48% | MATNGEDNQN QVSRHQEVGH KSLLQSDALY QYILETSVYP K**EPEPMKELR** EVTAK**HPWNI MTTSADEGQF LNMLLK**LINA KNTMEIGVYT GYSLLATALA LPDDGK**ILAM DINRENYELG LPVIEKAGVA HKIDFR**EGPA LPLLDQLIAD EKNHGSYDFI FVDADKDNYI NYHK**RLIDLV KVGGVIGYDN TLWNGSVVAP PDAPLRK**YVR **YYRDFVLELN K**ALAADPR**IE ICMLPVGDGI TLCR**RIK | CYT  CYSK  cytoER  ENDO | 6.5  5  4.5  1.5 | CHL  MIT  SECR  OTHER | 0.06 0.41 0.05  0.73 | CHL  MIT  SECR  OTHER | 0  0.02  0.03  0.95 | CYT  ENDO | 0.98  0.01 | CYT |
| 0108 | Hypersensitive induced response protein 3  *Triticumaestivum* | 21% | MGNLCCCVQV DQSTVAIREQ FGKFDSVLEP GCHCLPWIFG KRVVGHLTLR LQQLDVRCET KTKDNVFVTV VASIQYRPLA GKESDAYYKL TNTR**SQIQAY VFDVIR**ASVP KLNLDDAFVQ KNDIAK**AVED ELEK**AMSAYG FEIVQTLIVD IEPDAHVK**QA MNEINAAARM RVAANEK**AEA EKIVQIKRAE GEAEAK**YLSG LGIAR**QRQAI VDGLRDSVLG FSVNVPGTTA KDVMDMVLIT QYFDTMKEIG ASSK**SSAVFI PHGPGAVR**DI ATQIRDGLLQ GQSASDN | CYT  CYSK  CHL  NUCL | 9  2.5  2  2 | CHL  MIT  SECR  OTHER | 0.08 0.10 0.20  0.72 | CHL  MIT  SECR  OTHER | 0  0.01  0.01  0.98 | CYT | 0.95 | CYT |
| 0109 | Oxygen-evolving enhancer protein 1 (Fragments)  *Populus euphratica* | 81% | **RLTYDEIQSK** AEGINKNSPP DFQKTK**LMTR DGIDYAAVTV QLPGGERVPF LFTIKGGSTG YDNAVALPAG GRSKPETGEI IGVFESLQPS DTDLGAK**TPK | NUCL CHL CYS CYSK MIT | 3.5  3  3  2.5  2 | CHL  MIT  SECR  OTHER | 0.19 0.12 0.05  0.89 | CHL  MIT  SECR  OTHER | 0  0.01  0.03  0.96 | CYT  CHL | 0.46  0.32 | ? |
| 0113 | PsbP domain-containing protein  *Medicago truncatula* | 11% | MASISWFSCL HIRPTATAGD KGLSSPITVE HHKTRPQNLL SSSEEGLAIN RRQLILYTST AAIAASSTDS NALALNDVSE DFSIYTDDEN KFKIDIPQEW QIGTGESAGF KSLTAFYPKE QSNSNVSVVI TGVGPDFTK**M ESFGKVEEFA DTLVSGLDR**S WKKPPGVAAK LIDCKSSK**VL TASLPR**KNGS GFYFIEYTLQ SPGEGRKHLY SAIGMLTNGW YNRLYTVTGQ YGEEETDKYA SKIQKAVRSF KFI | CHL  NUCL  CYT  MIT | 7  3  2  1 | CHL  MIT  SECR  OTHER | 0.70 0.05 0.04  0.16 | CHL  MIT  SECR  OTHER | 0.72  0.66  0  0 | CHL  VAC | 0.83  0.09 | CHL |
| 0203 | Aldo/keto reductase AKR  *Manihot esculenta* | 20% | MAGAAVKRIK LGSQGLEVSA QGLGCMSMSA FYGPPKPESD MIALIHHAIN TGVTFFDTSD VYGPHTNEIL LGKALKGDIR KKVELATKFA INLKDGKREI RGDPAYVRAA CEASLKRLDV DCIDLYYQHR VDTSVPIEVT VGELKKLVEE GKIK**YIGLSE ASASTIR**R**AH AVHPITAVQL EWSLWSR**DVE EEIVPTCR**EL GIGIVAYSPL GR**GFFSSGPK LVETLSEGDF RKYLPR**FQPE NLEHNK**HLFE RVNEIAARKQ CTPSQLALAW VHHQGDDVCP IPGTTK**IENF NQNIGALSVK** LTPEDMAELE SIASASAVKG GRYGSDMGTY KDSDTPPLSS WKAV | CYT  CHL  NUCL  MIT | 7  2  2  2 | CHL  MIT  SECR  OTHER | 0.05 0.38 0.15  0.41 | CHL  MIT  SECR  OTHER | 0  0.99  0  0 | CYT  MIT | 0.92  0.06 | CYT/  MIT? |
| 0404 | Actin 2  *Gossypium hirsutum* | 71% | **MADTEEIQPL VCDNGTGMVK AGFAGDDAPR AVFPSIVGRP RHTGVMVGMG QKDAYVGDEA QSK**RGILTLK **YPIEHGIVSN WDDMEKIWHH TFYNELRVAP EEHPVLLTEA PLNPK**ANREK MTHIMFETFN VPAMYVAIQA VLSLYASGR**T TGIVLDSGDG VSHTVPIYEG YALPHAILRL DLAGRDLTDS LMKILTERGY MFTTTAER**EI VRDMKEK**LAY VALDYEQELE TAKSSSSVEK NYELPDGQVI TIGAERFRCP EVLFQPSLIG MEAAGIHETT YNSIMK**CDVD IK**KDLYGNIV LSGGSTMFPG IADR**MSKEIT ALAPSSMKIK VVAPPERKYS VWIGGSILAS LSTFQQMWIS K**GEYDESGPS IVHR**KCF | CYSK | 14 | CHL  MIT  SECR  OTHER | 0.17 0.110.09  0.88 | CHL  MIT  SECR  OTHER | 0  0.02  0.04  0.94 | CYT  CHL | 0.93  0.06 | CYSK |
| 0507 | Peroxidase  *Linum usita-tissimum* | 7% | MAPTSSHVVA LTIMLSAVLF ASTTTAQIPA PAKGMSWTFY KSSCPKLESI ITKRLKEVFK K**DIGQAAGLL R**LHFHDCFVE GCDGSVLLTG SAGGPSAEQG SPPNLSLRKE AFRIIDDLRA RVHKECGRVV SCSDIVALAA R**DSVVLSGGP KYQVALGRR**D GTTLVTQDTT LANLPPPFAT TGTILSSLAT KNLNPTDAVA LSGAHTIGIS HCSSFTDRLY PNQDPSMDQT FAKNLKATCP QAATTDNIVD IRSPNVFDNK YYVDLMNRQG LFTSDQDLYT DSRTRGIVTS FAINQTLFFE KFVVAMIKMG QISVLTGKQG EIRANCSVTN SAKVQTSSFL EEAVEEAVEL LAGMR | EXTR  CHL  CYT  MIT | 6  3  2  2 | CHL  MIT  SECR  OTHER | 0.060.01 0.87  0.06 | CHL  MIT  SECR  OTHER | 0.99  0.01  0  0 | CHL  ENDO  MIT | 0.17  0.17  0.15 | EXTR/  SECR? |
| 0606 | Putative alanine aminotransferase  *Oryza sativa*  *subsp. Jap.* | 14% | MSTAAAGAAP VSLDTINPKV LKCEYAVRGE IVTHAQLQQE LQKNPDSLPF DEILYCNIGN PQSLGQQPVT FFR**EVLSLCD HPALLDK**SET HALYSDAIER **AWQILDKIPG R**ATGAYSHSQ GIK**GLRDEIA AGIAAR**DGFH ASGDNIFLTD GASPAVHMMM QLLIRSENDG ILCPIPQYPL YSASIALHGG SLVPYFLDEE TGWGLEVDEL KKQLEEAQSK GITVRALVVI NPGNPTGQVL AEENQKKIVE FCKNEGLVLL ADEVYQENIY VEDKKFHSFK KIARSMGYTD DDLPLVSFQS VSKGYYGECG KRGGYMEVTG FSADVREQIY KVASVNLCSN VSGQILASLI MNPPKAGDES YESFMVEKDG ILSSLARRAK ALEEAFNSLE GITCNK**AEGA MYLFPR**IYLP QKAIGAAQAA GTAPDAYYAR RLLEATGIVV VPGSGFGQVP GTWHFR**CTIL PQEDKIPAII SK**FKEFHEK**F MDEFRD** | NUCL  CYT  CHL  VAC | 5  4  3  1 | CHL  MIT  SECR  OTHER | 0.430.07 0.17  0.56 | CHL  MIT  SECR  OTHER | 0.09  0.02  0  0.89 | CYT  PER | 0.93  0.06 | CYT/  NUCL? |
| 0703 | Actin 2  *Annona cherimola* | 61% | **MADTEDIQPL VCDNGTGMVK AGFAGDDAPR AVFPSIVGRP RHTGVMVGMG QKDAYVGDEA QSK**RGILTLK **YPIEHGIVSN WDDMEKIWHH TFYNELRVAP EEHPVLLTEA PLNPK**ANREK MTQIMFETFN VPAMYVAIQA VLSLYASGR**T TGIVLDSGDG VSHTVPIYEG YALPHAILRL DLAGRDLTDA LMK**ILTER**GY SFTTTAER**EI VRDMKEK**LAY VALDYEQELE TAK**SSSSVEK **SYELPDGQVI TIGAER**FRCP EVLFQPSLIG MEAAGIHETT YNSIMKCDVD IR**KDLYGNIV LSGGSTMFPG IADR**MSKEIT ALAPSSMKIK VVAPPERKYS VWIGGSILAS LSTFQQMWIA K**AEYDESGPS IVHRK**CF | CYSK | 14 | CHL  MIT  SECR  OTHER | 0.16  0.11  0.09  0.89 | CHL  MIT  SECR  OTHER | 0  0.01  0.03  0.94 | CYTO  CHLO | 0.90  0.06 | CYSK |
| 1201 | Pyruvate dehydrogenase E1 subunit beta  *Populus trichocarpa* | 28% | MLGIIRQKAF GQR**IRPAVSA WRGYSSAAK**E MTVR**EALNSA LDEEMSADPK VFLMGEEVGE YQGAYK**ISK**G LLDKYGPER**V LDTPITEAGF TGIGVGAAYH GLKPVIEFMT FNFSMQAIDH IINSAAK**SNY MSAGQISVPI VFR**GPNGAAA GVGAQHSHCY AAWYASCPGL KVLAPYSSED ARGLLKAAIR DPDPVVFLEN ELLYGETFPV SAEVLDSSFC LPIGKAKIEK EGKDVTITAF SKMVGYALKA AEILAK**EGIS AEVINLRSIR PLDR**NTINAS VRKTNRLVTV EEGFPQHGVG AEICASVVEE SFGYLDAPVE RIAGADVPMP YAANLER**LAV PQVEDIVR**AA KRACYRSV | MIT  CYT  CHL | 7  4  2 | CHL  MIT  SECR  OTHER | 0.05 0.94 0.02  0.06 | CHL  MIT  SECR  OTHER | 0  0.99  0  0 | MIT  PER | 0.96  0.03 | MIT |
| 1202 | Protein disulfide-isomerase  *Nicotiana tabacum* | 11% | MGRSRICNTL AILALFLFSS ALAEDVVVLT EENFEKEIGQ DR**AALVEFYA PWCGHCKK**LA PEYEKLGASF RKAKSILIGK VDCDEHKSVC SKYGVQGYPT IQWFPKGSLE PKKYEGGRTA EALAEFVNSE GGTNVKIAST PSSVVVLSPD NFDEIVLDET K**DVLVEFYAP WCGHCK**SLAP IYEKVATSFK QEEDVVIANL DADKHRDLGE K**YGVSGFPTL K**FFPKGNKAG EHYDGGRHLY DFVNFINEKS GTSRDSKGQF TLNAGIVESL DTLVNEFVSA TNEEKKAVFS KMEDEAGKLN GFAARYGKIY LKAAKSSMEK GADYAKNEIQ RLERMLAKSI SPAKSDEFTL KKNILATFA | CHL  EXTR | 9  4 | CHL  MIT  SECR  OTHER | 0  0.04  0.99  0.03 | CHL  MIT  SECR  OTHER | 0  0.30  0  0.69 | ENDO  VAC | 0.89  0.1 | ENDO |
| 1401 | Glucose-6-phosphate isomerase  *Populus*  *Trichocarpa* | 27% | MASLSGLCSS SPSLKPKHSL WKTTLNPPLL KTSLTYRTRT LLTPTRSIAS DIPADLSKTN DKLPNKPKQL GLEKDPNSLW R**RYVDWLYQH K**ELGLYLDVS RIGFTDEFVS EMEPRFHKAF KDMEELEKGA IANPDEGR**MV GHYWLR**NSTL APKSFLKTQI DKALDAVCDF ADQVVSGKIK TPDGGRFTQI LSVGIGGSAL GPQFVAEALA PDNPPLKIRF IDNTDPAGID HQIAQLGPEL ASTLVIVISK SGGTPETRNG LLEVQQAFRE AGLDFAKQGV AITQENSLLD NTAR**IEGWLA RFPMFDWVGG RTSEMSAVGL LPAALQGIDI R**EMLAGAALM DEANRTTVLR NNPAALLALC WYWASEGVGS K**DMVVLPYKD SLLLFSRYLQ QLVMESLGKE FDLDGNRVNQ GLTVYGNKGS TDQHAYIQQL REGVHNFFVT FIEVLR**DRPP GHDWELEPGV TCGDYLFGML QGTRSALYAK DR**ESITVTVQ EVTPR**SVGAL IGLYER**AVGI YASLVNINAY HQPGVEAGKK** AAGEVLALQK RVLAVLNEAS CKQPVEPLTI EEVADRCHAT EDIEMIYKII AHMAANDRAL IAEGSCGSPR  SLKVFLGECN VDELFA | CHL  MIT | 9  5 | CHL  MIT  SECR  OTHER | 0 0.04 0.99  0.03 | CHL  MIT  SECR  OTHER | 0  0.29  0  0.7 | CHL  ENDO | 0.91  0.03 | ? |
| 1402 | 26S protease regulat.subunit 6B  *Medicago Truncatula* | 54% | MGSSAMVLDP KPLTEPPPSL SSSSPADTDS SSTSDDLYTL LKSLQR**QLEF IDIQEEYVKD EQK**NLKRELL RAQEEVKRIQ SVPLVIGQFM EMVDTNNGIV GSTTGSNYYV RILSTINR**EL LKPSASVALH R**HSNALVDVL PPEADSSISL LSQSEKPDVT YNDIGGCDIQ KQEIR**EAVEL PLTHHELYKQ IGIDPPRGVL LYGPPGTGK**T MLAK**AVANHT TAAFIRVVGS EFVQK**YLGEG PRMVRDVFR**L AKENAPAIIF IDEVDAIATA RFDAQTGADR EVQRILMELL NQMDGFDQTV NVK**VIMATNR **ADTLDPALLR PGRLDRKIEF PLPDRR**QK**RL VFQVCTAKMN LSDEVDLEDY VSRPDKISAA EISAICQEAG MHAVRK**NRYV ILPKDFEKGY R**TNVKKPDTD FEFYK** | CYT  CYSK  NUCL | 10  2  1 | CHL  MIT  SECR  OTHER | 0.660.05 0.08  0.50 | CHL  MIT  SECR  OTHER | 0.98  0  0  0 | NUCL  CYT | 0.95  0.04 | CYT/  NUCL? |
| 1407 | Glucose-1-phosphate adenylyl transferase  *Glycine max* | 44% | MASMAAIGSL NVPCSASSRS SNVGRK**SFPR SLSFSASQLC GDK**IHTDSVS FAPKIGRNPV IVTPKAVSDS QNSQTCLDPD ASR**SVLGIIL GGGAGTR**LYP LTKKR**AKPAV PLGANYRLID IPVSNCLNSN VSKIYVLTQF NSASLNR**HLS RAYASNMGGY KNEGFVEVLA AQQSPENPNW FQGTADAVRQ YLWLFEEHNV LEFLVLAGDH LYR**MDYEKFI QAHRETDADI TVAALPMDEK R**ATAFGLMKI DEEGRIIEFA EKPKGEQLKA MK**VDTTILGL DDER**AKELPY IASMGIYVVS KNVMLDLLR**E KFPGANDFGS EVIPGATSIG MR**VQAYLYDG YWEDIGTIEA FYNANLGITK **KPVPDFSFYD RSSPIYTQPR** YLPPSK**MLDA DVTDSVIGEG CVIK**NCK**IHH SVVGLRSCIS EGAIIEDTLL MGADYYETEA DKR**FLAAKGS VPIGIGRNSH IKRAIIDKNA RIGENVK**IIN SDNVQEAARE TDGYFIK**SGI VTVIKDALIP SGTVI | CHL  MIT  CYT | 9  2.5  2 | CHL  MIT  SECR  OTHER | 0.660.040.09  0.49 | CHL  MIT  SECR  OTHER | 0.99  0  0  0 | CHLO  MITO | 0.96  0.03 | CHL |
| 1502 | Dihydrolipoyl lysine-residue acetyltransferase  *Arabidopsis thaliana* | 12% | MAYASRIINH SKKLKDVSTL LRRENAATIR YYSNTNRAPL NREDTFNSRL GYPPLERISI CSTSTLPVSI IFSTTRSNLS SAMGRPIFGK EFSCLMQSAR GFSSGSDLPP HQEIGMPSLS PTMTEGNIAR WLKKEGDKVA PGEVLCEVET DKATVEMECM EEGYLAKIVK AEGSKEIQVG EVIAITVEDE EDIGKFKDYT PSSTADAAPT KAEPTPAPPK EEKVKQPSSP PEPKASKPST PPTGDRVFAS PLARKLAEDN NVPLSDIEGT GPEGRIVKAD IDEYLASSGK GATAKPSKST DSKAPALDYV DIPHSQIRKV TASRLAFSK**Q TIPHYYLTVD TCVDK**LMALR SQLNSFKEAS GGKRISVNDL VVKAAALALR **KVPQCNSSWT DDYIR**QFKNV NINVAVQTEN GLYVPVVKDA DRKGLSTIGE EVRLLAQK**AK ENSLKPEDYE GGTFTVSNLG GPFGIK**QFCA VVNPPQAAIL AVGSAEKRVV PGNGPDQFNF ASYMPVTLSC DHRVVDGAIG AEWLK**AFKGY IENPK**SMLL | CHL  CYT  MIT | 6.5  5  1.5 | CHL  MIT  SECR  OTHER | 0.17 0.34 0  0.22 | CHL  MIT  SECR  OTHER | 0.07  0.95  0  0 | CHL  NUCL  MIT | 0.70  0.10  0.05 | CHL/ MIT |
| 1504 | Enolase  *Jatropha curcas* | 25% | MAFATSQPAT TTGNLLSKPF FSSKPQSSTS TSIPFPPLKS RSLVVRNSVT VAPPSAAKIA KECKIKSVKA RQIIDSRGNP TVEVDLITDD LYR**SAVPSGA STGIYEALEL RDGDK**SVYGG KGVLKAVKNI NDILGPKLIG VDVRNQDDVD AIMLDIDGTP NKSKLGANAI LGVSLSICRA GAGAKGLPLY KHIQELSGTK ELVMPVPAFN VINGGSHAGN NLAMQEFMIL PVGATSFAEA LRMGSEVYHT LKGIIK**AKYG QDACNVGDEG GFAPNVQDNR EGLVLLIDAI EK**AGYTGKIK **IGMDVAASEF LTK**DGKYDLN FKKQPNDGAH VLSAQNLGEL YKEFIKDFPI VSIEDPFDQD DWSSWASLQS SVDIQLVGDD LLVTNPKRIA EAIQKKACNG LLLKVNQIGS VTESIRAALD SK**AAGWGVMV SHRSGETEDN FIADLSVGLA SGQIK**TGAPC RSERLAK**YNQ LLRIEEELGN VRYAGEAFR**S P | CHL  MIT | 7  7 | CHL  MIT  SECR  OTHER | 0.97 0.03 0  0.15 | CHL  MIT  SECR  OTHER | 0.99  0  0  0 | CHL  MIT  CYT | 0.98  0.01  0.01 | CHL |
| 1507 | Enolase  *Spinacia oleracea* | 32% | MVTIKSVKAR **QIFDSRGNPT VEADIHLDDG TFARAAVPSG ASTGIYEALE LR**DGGKDYMG KGVFKAVQNV NEIIGPALVG KDPTEQTAID NFMVQELDGT TNEWGWCKQK LGANAILAVS LAVCKAGAQV KKIPLYQHIA EISGNKKMVL PVPAFNVING GSHAGNKLAM QEFMILPTGA SSFREAMKMG SEVYHHLKSV IKKK**YGQDAT NVGDEGGFAP NIQENKEGLE LLKTAIEKAG YTGKVVIGMD VAAAEFYSK**D KTYDLNFKEE NNDGSQKISG DALKDLYK**SF VSEYPIVSIE DPFDQDDWEH YGK**LTAEIGD K**VQIVGDDLL VTNPK**RVEKA INGKSCNALL LKVNQIGSVT ESIEAVKMSK R**AGWGVMASH R**SGETEDTFI ADLSVGLSTG QIKTGAPCRS ERLAKYNQLL RIEEELGDKA IYAGADFRAP VEPY | CYT  NUC  CHL  CYSK | 9.5  5.8  2  1,3 | CHL  MIT  SECR  OTHER | 0.1 0.15 0.11 0.72 | CHL  MIT  SECR  OTHER | 0  0.04  0.01  0.95 | MIT  VAC  CYT | 0.76  0.08  0.08 | CYT/  MIT? |
| 1509 | DEAD box RNA helicase  *Pisum sativum* | 52% | MAGVAPEGSQ FDAKQFDTKM NELLTTEGQD FYTFYEEVYD SFDAMGLQEN LLR**GIYAYGF EKPSAIQQR**G IVPFCKGLDV IQQAQSGTGK **TATFCSGILQ QLDYSVTECQ ALVLAPTR**EL AQQIEKVMRA LGDYLGVKVH ACVGGTSVRE DQR**ILSSGVH VVVGTPGRVF DMLRRQSLRP DYIKMFVLDE ADEMLSRGFK DQIYDIFQLL PSKIQVGVFS ATMPPEALEI TR**KFMNKPVR ILVK**RDELTL EGIKQFYVNV DKEEWK**LDTL CDLYETLAIT QSVIFVNTRR KVDWLTDKMR SRDHTVSATH GDMDQNTRDI IMREFRSGSS R**VLITTDLLA RGIDVQQVSL VINYDLPTQP ENYLHR**IGRS GRFGRK**GVAI NFVTKDDERM LGDIQKFYNV LIEELPSNVA ELL** | CYT  CHL  NUCL | 11  1  1 | CHL  MIT  SECR  OTHER | 0.11  0.07  0.09  0.94 | CHL  MIT  SECR  OTHER | 0  0.01  0.04  0.95 | CYT  NUCL  PER | 0.95  0.05  0.02 | CYT |
| 1601 | ATP synthase subunit beta  *Vitis vinifera* | 53% | MASRKLLSSL LRTSVR**RPIS RSVLSNPR**SP LPRPSPVGHL LARAANYASS ASAAAPAAST PSAAKGAGPS GKITDEFTGA GAIGQVCQVI GAVVDVRFDE GLPPILTALE VLDNSIR**LVL EVAQHLGENM VRTIAMDGTE GLVR**GQRVLN TGSPITVPVG RATLGR**IINV IGEPIDERGD IK**TDHFLPIH REAPSFVDQA TEQQILVTGI K**VVDLLAPYQ R**GGK**IGLFGG AGVGKTVLIM ELINNVAKAH GGFSVFAGVG ER**TREGNDLY REMIESGVIK LGEK**QSESKC ALVYGQMNEP PGAR**AR**VGLT GLTVAEHFRD AEGQDVLLFI DNIFRFTQAN SEVSALLGRI PSAVGYQPTL ATDLGGLQER** ITTTK**KGSIT SVQAIYVPAD DLTDPAPATT FAHLDATTVL SRQISELGIY PAVDPLDSTS RMLSPHILGE EHYNTAR**GVQ KVLQNYK**NLQ DIIAILGMDE LSEDDKLTVA R**ARKIQRFLS QPFHVAEVFT GAPGKYVELK **ESITSFQGVL DGK**YDDLSEQ SFYMVGGIEE VIAKAEKIAK ESAA | MIT  CHL | 12  2 | CHL  MIT  SECR  OTHER | 0.70  0.87  0  0 | CHL  MIT  SECR  OTHER | 0.49  0.92  0  0 | MIT  CHL  PER | 0.96  0.02  0.01 | MIT |
| 1603 | Betaine aldehyde dehydrogenase  *Amaranthus hypochondr.* | 17% | MAIR**VPSRQL FIDGEWR**EPI KKNRIPIINP STEEIIGVIP AATAEDVELA VAAARRALKR NKGEDWASAS GAHRAKYLRA IAAKITEKKD YFAKLEAMDC GKPLDEAAWD IDDVAGCFEY YADQAEALDA KQKAPIALPM DTFKCHVLKQ PIGVVGLISP WNYPLLMATW K**VAPALAAGC SAVLKPSELA SVTCLELAEV CR**EVGLPPGV LNILTGLGPE AGGPLACHPD VDKVAFTGST ATGSKVMSSA AQLVKPVTLE LGGK**SPIVIF EDVDLDK**AAE WTAFGCFWTN GQICSATSR**L LVHESIAAEF LDR**LVKWCKN IKISDPFEEG CRLGPVVSKS QYEKVLKFIS TAKSEGATIL CGGSRPEHLK KGYYVEPTII SDVSTSMQIW REEVFGPVLC VKTFGSEDEA IELANDTQYG LGAAVLSKDL DRCERITKAL QAGIVWVNCS QPCFCQAPWG GTKRSGFGR**E LGEWGIENYL NIK**QVTEYIS DEPWGWYKSP | CHL  CYT  MIT | 6  5  2 | CHL  MIT  SECR  OTHER | 0.26  0.14  0.09  0.59 | CHL  MIT  SECR  OTHER | 0  0.2  0.01  0.079 | CYT  CHL  PER | 0.88  0.07  0.04 | CYT |
| 1605 | Betaine-aldehyde dehydrogenase  *Corylus Heterphylla* | 17% | MAIPIPSR**QL FIDGEWREPA LR**KRFPIVNP STEEIVGDIP AATAEDVEIA VDAARRALAR NKGRDWASAP GAVRAKYLRA IAAKITERKS KLAKLEAIDS GKPLDEAAWD IDDVAGCFEY YADLADGLDA KQKAPVALPM ETFKSYVLQE PMGVVGLITP WNYPLLMATW KVAPALAAGC AAILKPSELA SVTCLELGEV CR**EVGLPPGV LNILTGLGHE AGAPLASHPH VDK**IAFTGST ITGSKIMTAA AQMVKPVSLE LGGKSPIIVF EDFDLDKAAE WTIFGCFWTN GQICSATSRL IVHESIAAEF LDKLVKWVKN IK**ISDPLEEG CRLGPVVSGG QYEK**ILKFIA TAKSEGATVA CGGARPEHLK KGFFIEPTII TDVTTSMQIW REEVFGPVLC VKTFSTEDEA IELANDTHYG LGAAVISKDL ERCDRVSKAL QAGIVWINCS QPCFTQAPWG GNKRSGFGRE LGEWGLNNYL SVK**QVTQYVT DEPWGWYQSP SK**L | CHL  CYT  PER | 6  5  2 | CHL  MIT  SECR  OTHER | 0.35  0.11  0.07  0.60 | CHL  MIT  SECR  OTHER | 0  0.06  0.02  0.91 | CYT  PER  MIT | 0.74  0.23  0.01 | CYT |
| 1701 | Chaperonin-60kD, ch60, putative  *Ricinus Commnis* | 26% | MYRFVSGLAS KARLAKNSTQ QVGSRLAWSR SYAAKDIKFG VEARALMLKG VEDLADAVK**V TMGPKGRNVV LEQSWGAPK**V TK**DGVTVAKS IEFQDR**VKNV GASLVKQVAN ATNDVAGDGT TCATVLTRAI LVEGCK**SVAA GMNAMDLR**RG ISMAVDTVIT NLKSRTRMIS TSEEIAQVGT ISANGER**EIG ELIAKAMEK**V GKEGVITISD GK**TLYNELEV VEGMKLDR**GY ISPYFVTNTK NQK**CELEDPL ILIHEK**KISN LNAVVKVLEL ALKKQRPLLI VAEDVESEAL ATLILNKLRA GIKVCAIK**AP GFGENRKASM QDLAVLTGGQ VITEELGMNL EK**VGAEAFGS CKKVTVSKDD TIILDGFGDK KALEERCEQL RSSIELSTSD YDKEKLQERL AKLSGGVAVL KIGGASEAEV SEKKDRVTDA LNATK**AAVEE GIVPGGGVAL LYASK**DLDKL HTANFDQK**IG VQIIQNALK**M PVHTIAANAG VEGAVVVGKL LEQDNPDLGY DAAKGEYVDM VKSGIIDPLK VIRTALVDAA SVSSLMTTTE AIVTELPKDE KEPTAMAGGG GGMGY | MIT  CHL | 11  3 | CHL  MIT  SECR  OTHER | 0.06  0.89  0.01  0.05 | CHL  MIT  SECR  OTHER | 0.01  0.99  0  0 | MIT  VAC  CHL | 0.98  0.01  0.01 | MIT |
| 1702 | 2,3-bisphospho glycerate-independent  phosphoglycerate mutase  *Ricinus commnis* | 21% | GEFTWKLADH PKLPKGKTIA MVVLDGWGEA KPDQYNCIHV AETPTMDSFK KTAPERWRLI KAHGTAVGLP TEDDMGNSEV GHNALGAGRI YAQGAKLVDL ALASGKIYEG EGFKYVKECF DK**GTLHLIGL LSDGGVHSR**L DQLQLLLKGA AEHGAKRIRV HVLTDGRDVI DGTSVGFAET LEKDLENLRE KGVDAQVASG GGRMYVTMDR YENDWNVVKR GWDAQVLGEA PYKFKSAVEA IKKLREEPKA NDQYLPPFVI VDENGKPVGP IVDGDAVVTI NFRADR**MVML AKALEYENFD TFDR**VRFPKI HYAGMLQYDG ELK**LPSHYLV SPPEIER**TSG EYLVHNGVHT FACSETVK**FG HVTFFWNGNR SGYFNPEMEE YVEIPSDVGI TFNVQPKMK**A IEIAEKARDA ILSGKFQQVR VNIPNGDMVG HTGDVEATVV GCKAADEAVK **MIIDAIEQVG GIYVVTADHG NAEDMVKR**DK SGKPMADKSG KIQILTSHTL QPVPIAIGGP GLTPGVRFRS DIPTGGLANV AATVMNLHGF EAPSDYEPTL IEAVDN | CYT | 14 | CHL  MIT  SECR  OTHER | 0.09  0.17  0.10  0.86 | CHL  MIT  SECR  OTHER | 0  0.02  0.05  0.92 | CYT  MIT | 0.98  0.01 | CYT |
| 1901 | Ubiquitin-activating enzyme E1, putative  *Ricinus Communis* | 11% | MLPSKRAGGG EVVLEEEKPI HNATTTTDPL PKKPRIISDS DSDFSATATT TATTGNAKST SAFNNNSTNT NSNTEKVAES PAMTLANGKS QDIDEDLHSR **QLAVYGR**ETM RKLFASNILI SGIQGLGAEI AKNLILAGVK SVTLHDEGDV ELWDLSSNFV FTEDDIGKNR ALAAVHKLQE LNNSVVISTS TSQLTKEQLS DFQAVVFTDI SLEKAVEFDD YCHNHQPPIA FIKSEVRGLF GSIFCDFGPD FTVLDADGED PHTGIIASIS NDNPALVACV DDERLEFQDG DLVVFSEVQG MPELNDGKPR KVKNSRPYSF QIDEDTTNHG AYVRGGIVTQ VKEPKVLNFK PLCEALKDPG DFLLSDFSK**F DRPPLLHLAF QALDK**FILEL GRYPIAGSDE DAQKLISFVA SINDSSADGR LEEIDQKLLR HFAFGAR**AVL NPMAAMFGGI VGQEVVK**ACS GKFHPLFQFF YFDSVESLPS EPLDHDDFRP LNSRYDAQIS VFGSKLQK**KL EDANIFMVGS GALGCEFLK**N LALMGVSCGE KGKLTITDDD VIEKSNLSRQ FLFRDWNIGQ AKSMVAASAA ALINPHFNIE ALQNRASPET  ENVFDDAFWE NLNVVVNALD NVNARLYIDA R**CLYFQKPLL ESGTLGAK**CN TQMVIPHLTE NYGASRDPPE KQAPMCTVRS FPHNIDHCLT WARSEFEGLL EKTPGEVNAY LNNPREYMTA MK**NAGDAQAK DNLERVLECL DKER**CVAFED CITWTR**LKFE DYFVNR**VKQL TFTFPEDALT SSGTPFWSAP KRFPRPLQFS ADDLSHLQFI MAASILR**AET FGIPIPDWVK** VPKKLADAIS NVIVPDFQPQ ENVKIETDEK ATSLPASSID DAAVIDELIM KLEKCHQQLL PGFRMNPIQF EKDDDTNYHM DLIAGLANMR ARNYGIPEVD KLKAKFIAGR IIPAIATSTA MATGLVCLEL YKVLDGGHKV EDYRNSFANL ALPLFSMAEP VPPKVIKHQD MSWTVWDRWI LRNNPTLREL LEWLQHKGLN AYSISYGSCL LFNSMFPRHR DRMDKKLVDL AREVGKAELP AYRRHFDVVV ACEDDEDKDI DIPQISIYFK | CYT  NUCL  CHL  MIT | 7  4  1  1 | CHL  MIT  SECR  OTHER | 0  0  0.01  0.98 | CHL  MIT  SECR  OTHER | 0.21  0.06  0.02  0.75 | CYT  CHL | 0.95  0.03 | CYT |
| 2004 | Proteasome subunit beta  *Glycine max* | 36% | MDQKVDFNAP HSMGTTIIGV TYNGGVVLGA DSR**TSTGVYV ANRASDKITQ LTDNVYVCRS GSAADSQIVS DYVRYFLHQH TIQLGQPATV K**VAANLVR**LL AYNNK**NFLQT GLIVGGWDKY EGGQIYGVPL GGTTVQQPFA IGGSGSSYLY GFFDQAWKEG MTKDEAEDLV KK**AVSLAIAR** DGASGGVVR**T VIINSEGVTR** NFYPGDQLPL WHEEMEAHNS LLDILGVPEP MSM | CYT  NUCL  EXTR | 9  2  2 | CHL  MIT  SECR  OTHER | 0.09  0.07  0.18  0.93 | CHL  MIT  SECR  OTHER | 0  0  0.01  0.98 | CYT  CHL  VAC | 0.37  0.33  0.15 | CYT |
| 2102 | Cysteine synthase  *Ricinus commnis* | 24% | MVEEKSAIAK DVTELVGKTP LVYLNHVVDG CVARIAAKLE MMEPCSSVKD RIGYSMITDA EEKGLIKPGE SVLIEPTSGN TGIGLAFMAA AKGYK**LIITM PASMSLER**RM VLR**AFGAELV LTDPAR**GMKG AVQKAEEILA K**TPNSYILQQ FENPANPKIH YETTGPEIWK** GSGGKVDFFV SGIGTGGTVT GAGGYLREQN PDIKLIGVEP VESAVLSGGK PGPHKIQGIG AGFIPGVLDV SLLDEVVQIS SEEAIETAKL LALKEGLLVG ISSGAAAAAA IKIARRPENA GK**LIVVIFPS FGERYLSSVL FESVKR**EAES MVFEP | CYT  CHL  CYSK  PLAS  EXTR | 6  3  2  1  1 | CHL  MIT  SECR  OTHER | 0.04  0.09  0.22  0.88 | CHL  MIT  SECR  OTHER | 0  0.02  0.03  0.95 | CYT  PER  VAC | 0.55  0.22  0.11 | CYT |
| 2105 | Aluminum induced protein with YGL and LRDR motif  *Arabidopsis thaliana* | 13% | MLAIFDKNVA KTPEALQGQE GGSVCALKDR FLPNHFSSVY PGAVTINLGS SGFIACSLEK QNPLLPR**LFA VVDDMFCIFQ GHIENVPILK** QQYGLTKTAT EVTIVIEAYR TLRDRGPYSA EQVVRDFQGK FGFMLYDCST QNVFLAGDVD GSVPLYWGTD AEGHLVVSDD VETVKKGCGK SFAPFPK**GCF FTSSGGLR**SY EHPSNELKPV PRVDSSGEVC GVTFKVDSEA KKEAMPRVGS VQNWSKQI | CHL  NUCL  CYT  MIT | 9  2  1  1 | CHL  MIT  SECR  OTHER | 0.1  0.07  0.06  0.85 | CHL  MIT  SECR  OTHER | 0.01  0  0.04  0.95 | CYT  PER  VAC | 0.88  0.09  0.01 | CYT |
| 2303 | Isocitrate dehydrogenase, putative  *Ricinus Communis* | 30% | MARRSIPILK KLLSSSNNES TCSRLVSRRS VTYMPRPGDG APRGVTLIPG DGIGPLVTGA VEQVMEAMHA PVYFER**YEVH GDMKK**VPAEV IESIKKNKVC LKGGLATPMG GGVSSLNVQL R**KELDLYASL VNCFNLPGLP TRHENVDIVV IRENTEGEYS GLEHEVVPGV VESLK**VITKF CSER**IAKYAF EYAYLNNR**KK VTAVHKANIM K**LADGLFLES CR**EVATKYPG IKYNEIIVDN CCMQLVSKPE QFDVMVTPNL YGNLVANTAA GIAGGTGVMP GGNVGADHAI FEQGASAGNV GNEKIVEQK**K ANPVALLLSS AMMLRHLQFP SFADR**LETAV ERVISEGKYR TKDLGGDSST QEVVDAVIAA LD | CHL  MIT | 10.5  2.5 | CHL  MIT  SECR  OTHER | 0.7  0.6  0.03  0.03 | CHL  MIT  SECR  OTHER | 0.42  0.96  0  0 | MIT  CHL  VAC | 0.95  0.03  0.01 | MIT |
| 2402 | S-adenosyl methionine synthase  *Camellia sinensis* | 47% | METFLFTSES VNEGHPDKLC DQISDAVLDA CLEQDQDSKV ACETCTK**TNM VMVFGEITTK** AAVDYEKIVR DTCR**TIGFVS DDVGLDADNC KVLVNIEQQS PDIAQGVHGH LTK**RPEEIGA GDQGHMFGYA TDETSELMPL SHVLATKLGA RLTEVR**KNGT CPWLRPDGK**T QVTVEYYNEK GATVPIR**VHT LLISTQHDET VTNDEIAADL KEHVIKPVIP DK**YLDEK**TIF HLNPSGRFVI GGPHGDAGLT GRKIIIDTYG GWGAHGGGAF SGKDPTK**VDR SGAYIVRQAA K**SIVANGLAR** RCIVQVSYAI GVPEPLSVFV DTYGTGKIPD KEILKIVK**ES FDFRPGMIAI NLDLKR**GGNS RFLK**TAAYGH FGR**DDPDFTW ESGEAPQVGQ TSS | CYSK  PLAS  CYT | 8.5  5.5  4 | CHL  MIT  SECR  OTHER | 0.12  0.12  0.15  0.86 | CHL  MIT  SECR  OTHER | 0  0.01  0.03  0.96 | CYT  PER | 0.95  0.05 | CYT |
| 2405 | S-adenosyl methionine synthase  *Elaegnus umbellata* | 58% | **METFLFTSES VNEGHPDKLC DQISDAVLDA CLAQDPDSK**V ACETCSK**TNM VMVFGEITTK** ANVDYEKIVR DTCR**TIGFVS DDVGLDADNC KVLVNIEQQS PDIAQGVHGH FTK**RPEEIGA GDQGHMFGYA TDETPELMPL SHVLATKLGA RLTEVR**KDGT CPWLRPDGKT QVTVEYYNDK GAMVPVRVHT VLISTQHDET VTNDEIAADL K**EHVIKPVVP EKYLDEK**TIF HLNPSGRFVI GGPHGDAGLT GRKIIIDTYG GWGAHGGGAF SGKDPTK**VDR SGAYIVRQAA K**SIVANGLAR** RCIVQVSYAI GVPDPLSVFV DSYGTGKIPD KEILKIVKEN FDFRPGMITI NLDLKRGGND RFLK**TAAYGH FGRDDPDFTW EIVKPLK**WEK PQS | CYT  CYSK | 7  6 | CHL  MIT  SECR  OTHER | 0.13  0.11  0.14  0.87 | CHL  MIT  SECR  OTHER | 0  0  0.03  0.96 | CYT  PER | 0.96  0.03 | CYT |
| 2603 | Leucine aminopeptidase  MTR_4g130860  *Medicago truncatula* | 28% | MAAIASAIVI AFSKSSSSSL FLTSRIRFAS FPKRSFHSTT KLMSQSRYTT LGLTHPTNIE APKISFSATD VDVTEWKGDI LAVGVTEKDL TRDAKSRFEN SILNKIDLKL DGLLSEASSE EDFSGKVGQS TVVRIKGLGS KRVGLIGLGQ LPSTTALYKG LGEAVVAVAK SAQASNVAIV LASSEGLSSE SKLSTAYAIA SGAVLGLFED QRYKSESK**KP AVRSIDIIGL GTGPDLEKK**L KYAGDVSSGI IFGRELVNSP ANVLTPGVLA EEASKVASTY SDVFTAKILD ADQCKELK**MG SYLGVAAASA NPPRFIHLTY KPPSGSVKVK** LALVGK**GLTF DSGGYNIKTG PGCSIELMKF DMGGSAAVLG AAK**ALGQIKP LGVEVHFIVA ACENMISGTG MRPGDVVTAS NGK**TIEVNNT DAEGRLTLAD ALVYACNQGV EK**IIDLATLT GACVVALGPS IAGVFSPNDE LVKEVLEASE VSGEKLWRLP IEESYWESMK **SGVADMVNTG GRPGGSITAA LFLK**QFVDEK **VQWLHIDMAG PVWNDK**KRSA TGFGVSTLVE WVLKNSS | CHL | 13 | CHL  MIT  SECR  OTHER | 0.90  0.09  0.14  0.01 | CHL  MIT  SECR  OTHER | 0.99  0  0  0 | CHL  MIT  PER | 0.93  0.05  0.01 | CHL |
| 2701 | 2,3-bisphospho glycerate-independent  Phosphoglycerate mutase  RCOM_1323170  *Ricinus commnis* | 28% | MGSSGEFTWK LADHPKLPKG KTIAMVVLDG WGEAKPDQYN CIHVAETPTM DSFKKTAPER WRLIK**AHGTA VGLPTEDDMG NSEVGHNALG AGR**IYAQGAK LVDLALASGK IYEGEGFKYV KECFDK**GTLH LIGLLSDGGV HSR**LDQLQLL LKGAAEHGAK RIRVHVLTDG RDVIDGTSVG FAETLEKDLE NLREKGVDAQ VASGGGRMYV TMDRYENDWN VVK**RGWDAQV LGEAPYK**FKS AVEAIKKLRE EPKANDQYLP PFVIVDENGK PVGPIVDGDA VVTINFRADR **MVMLAKALEY ENFDTFDR**VR FPKIHYAGML QYDGELK**LPS HYLVSPPEIE R**TSGEYLVHN GVHTFACSET VK**FGHVTFFW NGNRSGYFNP EMEEYVEIPS DVGITFNVQP K**MKAIEIAEK ARDAILSGKF QQVRVNIPNG DMVGHTGDVE ATVVACKAAD EAVK**MIIDAI EQVGGIYVVT ADHGNAEDMV KR**DKSGKPMA DKSGKIQILT SHTLQPVPIA IGGPGLTPGV RFRSDIPTGG LANVAATVMN LHGFEAPSDY EPTLIEAVDN | CYT | 14 | CHL  MIT  SECR  OTHER | 0.12  0.13  0.09  0.87 | CHL  MIT  SECR  OTHER | 0  0  0.02  0.98 | CYT  MIT | 0.98  0.01 | CYT |
| 3007 | Groes chaperonin, putative  *Ricinus communis* | 30% | MATAQLTASS VSLSARNLTS FEGLRPSTVK FASLKAGGLS QRSFRSLVVK AATVVAPK**YT SIKPLGDR**VL LKIKVAEEK**T DGGILLPTSA QTKPQGGEVV AVGEGR**TIGK NKLDISVKTG AQVVYSKYAG TEVEFNGSSH LILK**EDDIVG VLETDDIKDL KPLNDR**VFIK IADAEEK**TAG GLLLTEATK**E KPSIGTVIAV GPGTLDEEGN RRPLSVSPGN SVLYSK**YAGN DFK**GSDGTNY IALRASDVMA VLS | CHL | 13 | CHL  MIT  SECR  OTHER | 0.9  0.08  0.01  0.03 | CHL  MIT  SECR  OTHER | 0.99  0  0  0 | CHL  MIT  PER | 0.92  0.05  0.02 | CHL |
| 3205 | Aldo/keto reductase AKR  *Manihot esculenta* | 19% | MAGAAVKRIK LGSQGLEVSA QGLGCMSMSA FYGPPKPESD MIALIHHAIN TGVTFFDTSD VYGPHTNEIL LGKALKGDIR KKVELATKFA INLKDGKREI RGDPAYVRAA CEASLKRLDV DCIDLYYQHR **VDTSVPIEVT VGELKK**LVEE GKIK**YIGLSE ASASTIRR**AH AVHPITAVQL EWSLWSRDVE EEIVPTCR**EL GIGIVAYSPL GR**GFFSSGPK LVETLSEGDF RKYLPR**FQPE NLEHNK**HLFE RVNEIAARKQ CTPSQLALAW VHHQGDDVCP IPGTTK**IENF NQNIGALSVK** LTPEDMAELE SIASASAVKG GRYGSDMGTY KDSDTPPLSS WKAV | CYT  CHL  NUCL  MIT | 7  2  2  2 | CHL  MIT  SECR  OTHER | 0.05  0.38  0.15  0.42 | CHL  MIT  SECR  OTHER | 0  0.99  0  0 | CYT  MIT  CHL | 0.92  0.06  0.01 | CYT/  MIT |
| 3206 | Cysteine synthase  At3g59760  *Arabidopsis thaliana* | 33% | M**VAMIMASRF NR**EAKLASQI LSTLLGNR**SC YTSMAATSSS ALLLNPLTSS SSSSTLR**RFR CSPEISSLSF SSASDFSLAM KRQSRSFADG SER**DPSVVCE AVKRETGPDG LNIADNVSQL IGKTPMVYLN SIAKGCVANI AAKLEIMEPC CSVK**DR**IGYS MVTDAEQK**GF ISPGKSVLVE PTSGNTGIGL AFIAASRGYR LILTMPASMS MERRVLLKAF GAELVLTDPA KGMTGAVQKA EEILKNTPDA YMLQQFDNPA NPKIHYETTG PEIWDDTK**GK VDIFVAGIGT GGTITGVGR**F IKEKNPKTQV IGVEPTESDI LSGGKPGPHK IQGIGAGFIP KNLDQKIMDE VIAISSEEAI ETAKQLALKE GLMVGISSGA AAAAAIKVAK RPENAGK**LIA VVFPSFGER**Y LSTPLFQSIR EEVEKMQPEV | CHL  MIT | 9  5 | CHL  MIT  SECR  OTHER | 0.63  0.10  0  0.03 | CHL  MIT  SECR  OTHER | 0.99  0.01  0  0 | MIT  CHL  VAC | 0.71  0.27  0.01 | CHL/  MIT |
| 3302 | Glutamine synthetase  CsGS1  *Vitis vinifera* | 34% | M**ALLSDLINL NLSDVTEKII AEYIWIGGSG MDLR**SKARTL SGPVSDPHKL PKWNYDGSST GQAPGEDSEV ILYPQAIFKD PFRRGNNILV MCDAYTPAGE PIPTNKRHNA AKIFSHPDVL AEETWYGIEQ EYTLLQNSVK WPIGWPVGGY PGPQGPYYCG IGADKAFGRD IVDSHYKACL YAGINISGIN GEVMPGQWEF QVGPSVGISA GDELWVARYI LER**ITEIAGV VVSFDPKPIQ GDWNGAGAHT NYSTK**SMRND GGYEIIKKAI EKLGLR**HKEH IAAYGEGNER** R**LTGRHETAD INTFLWGVAN R**GASIRVGRD TEKEGKGYFE DR**RPASNMDP YVVTSMIAET TILWKP** | CYT  NUCL | 11  2 | CHL  MIT  SECR  OTHER | 0.11  0.05  0.29  0.72 | CHL  MIT  SECR  OTHER | 0  0.01  0.01  0.98 | CYT  PER  VAC | 0.92  0.07  0.01 | CYT |
| 3303 | Flavanone 3-hydroxylase  F3H  *Ampelopsis Grossedent.* | 54% | M**APTTLTALA GEKTLQSSFV RDEDERPK**VA YNEFSNEIPI ISLEGIDEVG GRRDEICRKI VEACEDWGIF QVVNHGVDSN LISEMTRLAR **EFFALPPEEK LRFDMSGGKK GGFIVSSHLQ GEAVQDWREI VTYFSYPLR**T RDYSR**WPDKP EGWKSVTQEY SEKLMGLACK** LLEVLSEAMD LEKEALTKAC VDMDQKVVVN FYPQCPQPGL TLGLK**RHTDP GTITLLLQDQ VGGLQATRDG GKTWITVQPV EGAFVVNLGD HGHYLSNGR**F KNADHQAVVN SNYSR**LSIAT FQNPAPEATV YPLK**IREGEK **AVLEEPITFA EMYR**RKMSKD LELARLKKLA K**EQQLQDLEK** AKLESKPIDE IFA | CYT  CHL  CYSK  NUCL | 7  3  2  1 | CHL  MIT  SECR  OTHER | 0.10  0.18  0.08  0.8 | CHL  MIT  SECR  OTHER | 0  0.19  0  0.81 | CYT  ENDO | 0.98  0.01 | CYT |
| 3503 | Enolase PGH1  *Alnus glutinosa* | 34% | **MAEITHIKAR** QIFDSRGNPT VEAEVTTANG VVSRAAVPSG ASTGVYEALE LRDGGSDYLG KGVLKAVDNV NKIIGPALIG KDATEQTAID IDFMFQQLDG TVNEWGWCKQ KLGANAILAV SLAVCKAGAS VKKIPLYKHI ANLAGNPK**LV LPVPAFNVIN GGSHAGNKLA MQEFMILPVG ASSFK**EAMKM GVEVYHHLKA VIKKK**YGQDA TNVGDEGGFA PNIQENKEGL ELLK**TAIAKA GYVVIGMDVA ASEFYEKDKD ITNFKEENND GSQKISADQL KDLYKSFVDE YPIVSIEDPF DQDDWEHYSK LTAEIGEK**VQ IVGDDLLVTN PK**RVEKAIKE KACNALLLK**V NQIGSVTESI EAVK**MSKRAG WGVMAHR**SGE TEDTFIADLS VGLATGQIK**T GAPCRSERLA K**YNQLLRIEE ELGSEAVYAG ANFR**TPVEPY | CYT  PER  CYSK  CHL | 8  2  2  1 | CHL  MIT  SECR  OTHER | 0.25  0.18  0.07  0.70 | CHL  MIT  SECR  OTHER | 0  0.02  0  0.98 | CYT  MIT  ENDO | 0.94  0.03  0.01 | CYT |
| 3604 | TCP domain class transcription factor  TCP3  *Malus Domestica* | 35% | MAVERIFKDE ASEEKGER**AR LASFVGAIAI ADLVKTTLGP KGMDK**ILQST GRGHAVTVTN DGATILKSLH IDNAAAKVLV DISK**VQDDEV GDGTTSVVVL AGELLR**EAEK LVASK**IHPMT IISGYR**MAAE CARDALLRKV VDNKADSEKF KSDLMKIAMT TLSSKILSQD KEHFAQLAVD AVMRLKGSTN LEAIQIIKKP GGSLIDSFLD EGFILDKKIG LGQPKRIENA NILVANTAMD TDKVKIYGAR VRVDSMAKVA EIEGAEKDKM REKVQKIIGH GINCFVNRQL IYNFPEELFA DAGILAIEHA DFDGIER**LAL VTGGEIASTF DNPESVK**LGH CKLIEEIMIG EDK**LIHFSGV ELGQACTIVL RGASHHVLDE AERSLHDALC VLSQTVNDSR** VLLGGGWPEM IMAKEVDELA RKTPGKK**SHA IEAFSR**ALSA IPTIIADNAG LDSAELIAKL R**AEHQKEGCT SGIDVISGTV GDMAER**GISE AFKVK**QAVLL SATEAAEMIL RVDEIITCAP R**KREDRM | CYT  CHL  CYSK | 6  5  2 | CHL  MIT  SECR  OTHER | 0.12  0.12  0.06  0.86 | CHL  MIT  SECR  OTHER | 0  0  0.03  0.97 | CYT  NUCL  MIT | 0.53  0.19  0.1 | CYT |
| 3607 | Mitochondrial processing peptidase beta subunit  *Cucumis melo* | 30% | MAIKQILTLA RTSHRRSPAL FSQAVR**SAST FPAVASSSPL PSPPPPNAMI YDR**LAEAVKS KLKQLENPDP RFLKYGSPHP TITDHTRILS APETRVTTLS NGLR**VATESN LAARTATVGV WIDAGSR**FET EETNGTAHFL EHMIFKGTEK RSAR**QLEEEI ENMGGHLNAY TSR**EQTTYYA KVLDKDVPK**A LDILADILQN SK**FDEHRISR ERDVILREME EVEGQTEEVI FDHLHATAFQ YTPLGRTILG PAQNIR**TITK DHLQSYIQTH YTAPR**MVIAA SGAVKHEDFV EQVKKLFTKL SAEPTTAAQL VAKEPAIFTG SEVRIVDDDV PLAQFAIAFN GASWTDPDSI ALMVMQAMLG SWNKSAGGGK HMGSELAQR**V AINEVAESMM AFNTNYK**DTG LFGVYAVAKP DCLDDLAYAI MYETTKLAYR VSEADVTRAR NQLK**SSLLLH IDGTSPVAED IGRQLLTYGR RIPFAELFAR** IDAVDASTIK RVANRFIYDR DIAIAALGPI QGLPDYNWFR RR**TYWNRY** | MIT | 13.5 | CHL  MIT  SECR  OTHER | 0.59  0.65  0  0.03 | CHL  MIT  SECR  OTHER | 0.03  0.99  0  0 | MIT  PER  VAC | 0.87  0.11  0.01 | MIT |
| 3703 | Pyruvate decarboxylase  *Ricinus commnis* | 22% | MDTNIGSIDT LKPTTTDVCC PANGAVCTLQ SSTVSPSSIV SSPDSTLGRH LARRLVQVGV TDVFSVPGDF NLTLLDHLIA EPGLNVIGCC NELNAGYAAD GYARSRGVGA CVVTFTVGGL SVLNAIAGAY SENLPVVCIV GGPNSNDYGT NR**ILHHTIGL PDFSQELR**CF QPVTCFQAIV NNLEDAHELI DTAISTALKE SKPVYLSISC NLSAIPHPTF SR**EPVPFSLS PR**LSNK**IGLE AAVEAAAEFL NKAVKPVLVG GPK**LRVAKAC EAFVELADAC GYALAVMPSA KGLVPEHHSH FIGTYWGAVS TAFCAEIVES ADAYLFAGPI FNDYSSVGYS LLLKKEKSII VQPDRVVIGN GPAFGCVLMK DFLKALAKRL KNNTTAHENY RRIFVPEGQP LKSQPKEPLR VNVLFQHIQK MLSSETAVIA ETGDSWFNCQ KLKLPKGCGY EFQMQYGSIG WSVGATLGYA QAVPEKR**VIA CIGDGSFQVT AQDVSTMLR**C GQKTIIFLIN NGGYTIEVEI HDGPYNVIK**N WNYTGLVDAI HNGEGK**CWTA K**VQCEEELIE AIETATESKK DCLCFIEVIA HKDDTSKELL EWGSR**VSAAN  SRPPNPQ | CYT  ENDO CHL  MIT  PLAS  VAC | 5  4  1  1  1  1 | CHL  MIT  SECR  OTHER | 0.61  0.03  0.01  0.65 | CHL  MIT  SECR  OTHER | 0.09  0  0.08  0.83 | CYT  CHL  VAC | 0.81  0.18  0.01 | CYT |
| 3705 | Pyruvate decarboxylase  *Prunus armeniaca* | 11% | MDTKIGALDV CKPASNEVGS LPNGTAMAIQ NSVPSTVINS SEATLGRHLA RRLVQIGVTD VFTVPGDFNL TLLDHLIAEP GLTNIGCCNE LNAGYAADGY ARSRGVGACV VTFTVGGLSV LNAIAGAYSE NLPLICIVGG PNSNDYGTSR **ILHHTIGLPD FSQELR**CFQT VTCYQAVVNN LEDAHESIDT AISTALKESK PVYISISCNL AGIPHPTFSR **EPVPFSLSPR** LSNQWGLEAA VEAAAEFLNK AVKPVMVGGP KLRVAHAGEA FVELADTSGY ALAVMPSAKG LVPEHHPHFI GTYWGAVSTA FCAEIVESAD AYLFAGPIFN DYSSVGYSLL LKKEK**AIIVQ PDR**VTIANGP SFGCVLMKDF LRALAKKLNH NNTAHENYRR IFVPDGHPLK CAPREPLRVN VLFQHVQKML SSETAVIAET GDSWFNCQKL RLPAGCGYEF QMQYGSIGWS VGATLGYAQA VPEKRVLAFI GDGSFQVTAQ DVSTMIRNGQ RTIIFLINNG GYTIEVEIHD GPYNVIK**NWN YTGLVDAIHN GEGK**CWTTKV RYEEELIEAI ETATGDKKDS LCFIEVIVHK DDTSK**ELLEW GSRVSAANSR**  **PPNPQ** | CYT  ENDO  PLAS CHL  MIT | 5  4  2  1  1 | CHL  MIT  SECR  OTHER | 0.41  0.05  0.01  0.65 | CHL  MIT  SECR  OTHER | 0  0.03  0  0.97 | CYT | 0.99 | CYT |
| 3706 | Ribulose bisphosphate carboxylase large chain rbcL  *Noronhia emarginata* | 34% | MSPQTETKAS VGFKAGVKEY K**LTYYTPEYE TKDTDILAAF R**VTPQPGVPP EEAGAAVAAE SSTGTWTTVW TDGLTSLDRY KGRCYHIEPV PGEADQYICY VAYPLDLFEE GSVTNMFTSI VGNVFGFKAL R**ALRLEDLRI PTAYVKTFQG PPHGIQVER**D KLNK**YGRPLL GCTIKPKLGL SAK**NYGRAVY ECLR**GGLDFT KDDENVNSQP FMR**WRDRFLF CTEAIYKSQS ETGEIK**GHYL NATAGTCEEM IKR**AVFAREL GVPIIMHDYL TGGFTANTSL AHYCR**DNGLL LHIHRAMHAV IDR**QKNHGIH FRVLAKALRM SGGDHIHAGT VVGKLEGER**D ITLGFVDLLR DDFIEK**DRSR GIYFTQDWVS LPGVIPVASG GIHVWHMPAL TEIFGDDAVL QFGGGTLGHP WGNAPGAVAN RVALEACVKA RNEGR**DLASE GNVIIR**EASK **WSPELAAACE VWKEIK**FEFA AVDTL | CYT  PER  MIT | 8  4  2 | CHL  MIT  SECR  OTHER | 0.06  0.14  0.15  0.87 | CHL  MIT  SECR  OTHER | 0  0  0.02  0.98 | CYT  PER  MIT | 0.72  0.26  0.01 | CYT/ PER? |
| 3707 | Phosphogluco-mutase, cytoplasmic PGM1  *Populustremula* | 32% | MVLFNVSRVE TTPFGDQKPG TSGLRKKVKV FKQPNYLQNF VQSTFNALTP QNVR**GATLVV SGDGR**YFSKD AIQIITKMAA GNGLR**RVWVG QNGLLSTPAV SAVIR**ERVGV DGSKATGAFI LTASHNPGGP NEDFGIKYNM ENGGPAPEGI TDKIYENTKT IKEYLTADLP DVDITTIGVT SFSGSEGQFD VEVFDSASDY IKLMKSIFDF ESIRKLLSSP K**FTFCYDALH GVAGAYAK**RI FVEELGAQES SLLNCVPK**ED FGGGHPDPNL TYAK**ELVARM GLGKSNSEVE PPEFGAAADG DADRNMVLGK RFFVTPSDSV AIIAANAVEA IPYFSAGLK**G VARSMPTSAA LDVVAK**SLNL K**FFEVPTGWK** FFGNLMDAGL CSVCGEESFG TGSDHIREKD GIWAVLAWLS ILAYKNRENL GGGK**LVTVED IVHNHWATYG RHYYTRYDYE NVDAGAAK**EL MACLVK**LQSS LTEVNEIVSG IQSDVSKVVH ADEFEYKDPV DGSISK**HQGI R**YLFEDGSR**L VFRLSGTGSE GATIR**LYIEQ YEKDPSK**TGR DSQDALAPLV AVALGLXK**MQ EFTGR**SAPTV IT | CYT  CHL | 10  4 | CHL  MIT  SECR  OTHER | 0.06  0.33  0.02  0.93 | CHL  MIT  SECR  OTHER | 0.05  0.92  0  0 | PER  CYT  CHL | 0.74  0.13  0.07 | ? |
| 3803 | Uncharacterized protein app1 Xaa-Pro amino peptidase 1  *Solanum lycopersicum* | 8% | MADTLAALRS LMSSHSPSLH ALIIPSEDYH QSEYVSARDK RRAFVSGFTG SAGLALITMD EALLWTDGRY FLQAAQQLSD QWKLMRMGED PPVDIWMANN LPKDAAIGVD TWCVSVDTAQ KWECAFAKKQ QKLVQTTRNL VDDVWKNRLP AQANPVIVHP LQFAGQSVAE KLKELRKKLV MGKACAIIIT ALDEVAWLYN VRGSDVSYCP VVHAFAIVTI DSAFFYVDKQ KLSPEANSYM EENGIMVRDY GDVSSDVVLL ASDQLTSCSS TKGSKGNPKI DVRNATYVGN SDSHAAEFVN DLIWVDPGAC CFALYSKLSA DKVLLQQSPL ALAKALKNPV EIEGLKKAHF RDGAAVVQYL VWLDK**QMQEI YGASGYFMEA ESTKQK**KQLG TKRLTEVSVS DKLEEFRASK EHFRGLSFRT TSSVGSNAAI IHYKPEAETC AELDPDCIYL FDSGAQYLDG TTDITRTIHF GKPSPHEKSS YTAVLKGHIS LGNARFPNGT NGQALDILAR IPLWKDGLDY RHGTGHGIGS YLNVHEGPHN ISFRPSARDV PLQVSMAVTD EPGYYEDGNF GIRIENVLIV KEGHTKFNFG NK**GYLSFEHI**  **TWAPYQR**KLI DVSLLIPEEI EWLNEYHAK**C REILTPYLNT SEMEWLK**KAT EPIAA | CHL  CYT | 10  3 | CHL  MIT  SECR  OTHER | 0.24  0.08  0.16  0.49 | CHL  MIT  SECR  OTHER | 0.55  0.22  0  0.23 | MIT  CYT  VAC | 0.74  0.13  0.04 | CHL |
| 3805 | Glycyl-tRNA synthetase  ARALYDRAFT_473197  *Arabidopsis lyrata subsp.*  *lyrata* | 16% | MRIFSTSVFP RR**QQQQIFNL R**QFQTTTILR NPISIAPIQI PMDAEQSLRQ SLSEKSSSVE AQGNAVRALK ASR**AAKPEID AAIEQLNR**LK LEKSAVEKEL QSIISSSGNG SLNREAFRKA VVNTLER**RLF YIPSFK**IYSG VAGLFDYGPP GCAIKSNVLS FWRQHFILEE NMLEVDCPCV TPEVVLKASG HVDKFTDLMV KDEKTGTCYR ADHLLKDYCT EKLEKDLTIS AEKAAELKDV LAVMEDFSPE QLGAKIKEYG ITAPDTKNPL SDPYPFNLMF QTSIGPSGLI PGYMRPETAQ GIFVNFKDLY YYNGKK**LPFA AAQIGQAFR**N EISPRQGLLR **VREFTLAEIE HFVDPENK**SH PK**FSDVAKLE FLMFPR**EEQM SGQSAKKLCL GEAVSKGTVN NETLGYFIGR **VYLFLTR**LGI DKERLRFRQH LANEMAHYAA DCWDAEIESS YGWIECVGIA DRSAYDLR**AH SDKSGVPLVA EEK**FAEPKEV EKLVITPVKK ELGLAFKGNQ KNVVESLEAM NEEEAMEMKA SLESKGEVEF YVCTLNKTVS IKKNMVSISK EKKKEHQR**VF TPSVIEPSFG IGR**IIYCLYE HCFSTRPSKA GDEQLNLFR**F PPLVAPIK**CT VFPLVQNQQF EEAAKVISKE LASVGISHKI DITGTSIGKR YARTDELGVP FAITVDSDAS VTIRERDSKD QVRVSLKEAA SVVSSVSEGK MTWQDVWASF PHHSSAAADE | CHL  MIT | 8  6 | CHL  MIT  SECR  OTHER | 0.07  0.90  0  0.09 | CHL  MIT  SECR  OTHER | 0.12  0.94  0  0 | MIT  CHL  CYT | 0.66  0.17  0.14 | MIT |
| 3806 | NADH-ubiquinone oxidoreductase 75 kDa subunit  *Zea mays* | 13% | MAFLARALRQ SNSRLSSCPS VAASCRWISR TAAAGSPEAG AAVAPADPEL PPPREPVGGA RVELPPNPED ALEVFVDGHA VRIPKGFTVL QACEVAGVDI PR**FCYHSR**LS IAGNCRMCLV EVEKSPKPVA SCAMPALPGM KIKTNTPVAK KAREGVMEFL LMNHPLDCPI CDQGGECDLQ DQSMAFGADR GRFTEMKRSV VDK**NLGPLVK TVMTR**CIQCT RCVR**FATEVA GVQDLGMLGR** GSGEEIGTYV EKLMTSELSG NVIDICPVGA LTSKPFAFKA RNWELKGTET IDVTDAVGSN IRVDSRGPEV MRIVPR**LNED INEEWISDKT RFCYDGLKR**Q RLNDPMIRGP DGRFKAVTWR DALAVVAEVL HQVKPEEITG VAGKLSDAES MMALKDFVNR MGSDKVLCEG NGPNPPADLR SNYLMNTSIA GLEK**ADVFLL VGTQPR**VEAA MVNARIRKTV KATQAKVGYI GPPADFNYDH EHLGTGPQTL VEITEGRHPF CSVLQSAK**NP VIIAGAGLFE REDQDALFSM VETVAKK**FNV TRPDWNGLNV LLLHAAQAAA LDLGLVANPA ESIKSAKFLY LMGADDISLD KLPDDAFVVY  QGHHGDKAVY RANVILPSSA FSEKEGTYEN TEGCTQWTIP AVPTVGDARD DWKIIRALSE VAGALLPYDS LSAVRDRIST VAPNLVHVDE REPSTISVEV KPPVKQKVSS TPFKTVVENF YMTDAITRAS KIMAQCSATL LKK | CHL  MIT | 11  2.5 | CHL  MIT  SECR  OTHER | 0.28  0.84  0.03  0.01 | CHL  MIT  SECR  OTHER | 0.02  0.99  0  0 | MIT  CHL  VAC | 0.93  0.04  0.01 | MIT |
| 4201 | Aldo/keto reductase  *Manihot esculenta* | 20% | MAGAAVKRIK LGSQGLEVSA QGLGCMSMSA FYGPPKPESD MIALIHHAIN TGVTFFDTSD VYGPHTNEIL LGKALKGDIR KKVELATKFA INLKDGKREI RGDPAYVRAA CEASLKRLDV DCIDLYYQHR VDTSVPIEVT VGELKKLVEE GKIK**YIGLSE ASASTIRRAH AVHPITAVQL EWSLWSR**DVE EEIVPTCR**EL GIGIVAYSPL GR**GFFSSGPK LVETLSEGDF RKYLPR**FQPE NLEHNK**HLFE RVNEIAARKQ CTPSQLALAW VHHQGDDVCP IPGTTK**IENF NQNIGALSVK** LTPEDMAELE SIASASAVKG GRYGSDMGTY KDSDTPPLSS WKAV | CYT  CHL  NUCL  MIT | 7  2  2  2 | CHL  MIT  SECR  OTHER | 0.05  0.38  0.15  0.42 | CHL  MIT  SECR  OTHER | 0  0.99  0  0 | CYT  MIT  CHL | 0.92  0.06  0.01 | CYT/ MIT |
| 4507 | UDP-glucosyl transferase, putative RCOM_0865380  *Ricinus communis* | 12% | MSREIFVVPA FGQGHLLPCL ELCKHLAASL NFKIVLVIFS DLSSSIPASL R**HENPLIEVA QIQSPPQSFS HPFHK**MHNDQ IQLSLGLESL LSSR**TQSLPV CAIVDVLLVM GWTSQVFK**KF QVATVGFFTS GACSTAMEYA TWKAHPIDLK PGELRLIPGL PEQMALTVSD IKRRPHGGPQ GGGGVGGSKK FGPPNPGERP PWVDDTEDSI ALIINTCDDL ERPFIEYVAN EIRKPVWGIG PLLPQKYWES AGSILHDREI RSNRGSTVTE DQVMDWLDSK AERSVIYISF GSELGPTMEE YPHLAAAIEA WTGPFIWVIQ PGSGRPGPPG TVKAEEGYFP HGLDKKVGER GLIIRGWAPQ LLILSHPSTG GFLSHCGWNS TVEAIGR**GVP FLAWPIR**GDQ YYDAKLVVSY LKMGYMVSDD MSKMITDDNV IQGIHRLMGD DEVKRRADII RSKFVHGFPA SSLLALGAFK DFINQRLA | CHL  NUCL  EXTR  CYT  MIT | 5  3  3  1  1 | CHL  MIT  SECR  OTHER | 0.01  0.1  0.35  0.54 | CHL  MIT  SECR  OTHER | 0  0.02  0.01  0.96 | ENDO  CHL  VAC | 0.26  0.19  0.17 | SECR |
| 4605 | POPTRDRAFT_863097 Methylmalonate-semialdehyde dehydrogenase (acylating) activity  *Populus trichocarpa* | 3% | MKVPNFIGGK FVVSQGCTII DVLNPATQEV VSHLPLTTYE EFKDAVIAAK RAFPSWKNTP IATRQRVMFR FQELIRRDMD KLATSITSEQ GKTLKGALGD VLCGLEAVEH ACAMATLQMG EFVPNASNGI DTYCIREPLG VCAGICPFNF PAMIPLWMFP IAVTCGNTFV LKPCEKNPGA SMILAALAVE AGFPDGVLNV IHGTNDIVNY ICDDDDVKAI SFIGSDLAGL HIYARAAARG KRVQSNIGGK NHAIILPDAS IDDTLNALVA AGFGAAGQRC MALSTAVFVG GSSAWEHELV EHAKALKVNA GTDPSADLGP VISKEVKDRI CRLVQSGVDS GARLLLDGRN IVVPGYENGS FVGPTILCDV TISMECYKEE ILGPVLLCMQ ADSLEEAITI VNRNR**YGNGA SIFTTSGVAA R**KFQNDIDAV LVGINVSVPV PLPCSSFHEA  KVSFAGNLNF CGKTGVQFYT QIKTVAQQWR ELPSIGVSLS MHTSNE | CYT  CHL | 9  5 | CHL  MIT  SECR  OTHER | 0.06  0.04  0.55  0.52 | CHL  MIT  SECR  OTHER | 0  0  0.02  0.98 | PER  MIT  CYT | 0.49  0.33  0.14 | CYT/  PER |
| 4702 | D-3-phospho glycerate dehydrogenase putative  RCOM_0811570  *Ricinus communis* | 23% | MAISTTNLRT TVNRNLSSFS VASK**THKLST FSVPLR**NSRR NSRFIVLTAS LDAKPTVLVT EKLGEAGLNL LKEFANVDCS YNLSPEELCT K**ISLCDALIV R**SGTKVNREV FESSGGRLKV VGRAGVGIDN VDLSAATEHG CLVVNAPTAN TVAAAEHGIA LLAAMARNVA QADASVKAGK WQRNKYVGVS LVGKTLAVMG FGKVGSEVAR RAK**GLGMHVI AHDPYAPADR** ARAIGVELVS FDEAIGTADF ISLHMPLTPA TSKILNDENF AKMKKGVRIV NVAR**GGVIDE DALVR**AIDAG IVAQAALDVF TEEPPAKDSK LVQHEKVTVT PHLGASTVEA QEGVAIEIAE AVVGALKGEL AATAVNAPMV PAEVLTELKP FVMLAEKLGR **LAVQLVAGGS GVK**TVKVTYG STRAPDDLDT RLLRAMITK**G LIEPISSVFV NLVNADFTAK** QRGLRIAEER VTLDGSPESP LEFIQVQIAN VESKFASAIS ESGEIKVEGK **VKDGIPHLTK VGSFEVDVSL EGSIILCRQV DQPGMIGKVG SILGEENVNV SFMSVGR**IAP RKQAVMAIGV DDQPKKESLK KIGDIPAIEE FVFLKL | MIT  CHL | 11  3 | CHL  MIT  SECR  OTHER | 0.88  0.43  0  0.02 | CHL  MIT  SECR  OTHER | 0.99  00  0  0 | MIT  CHL  PER | 0.76  0.18  0.04 | CHL/ MIT |
| 4704 | Methylene  tetrahydrofolate reductase 2  MTHFR2  *Arabidopsis thaliana* | 6% | MKVIDKIQSL ADEGKTAFSF EFFPPKTEDG VDNLFERMDR MVAYGPTFCD ITWGAGGSTA DLTLDIASRM QNVVCVESMM HLTCTNMPVE KIDHALETIR SNGIQNVLAL RGDPPHGQDK FVQVEGGFDC ALDLVNHIRS KYGDYFGITV AGYPEAHPDV IGENGLASNE AYQSDLEYLK KKIDAGADLI VTQLFYDTDI FLKFVNDCRQ IGISCPIVPG IMPINNYRGF LRMTGFCKTK IPVEVMAALE PIKDNEEAVK AYGIHLGTEM CKKMLAHGVK SLHLYTLNME KSALAILMNL GMIDESKISR SLPWRRPANV FRTK**EDVRPI FWANRPK**SYI SRTKGWEDFP QGRWGDSRSA SYGALSDHQF SRPRARDKKL QQEWVVPLKS VEDIQEKFK**E LCLGNLKSSP WSELDGLQPE TR**IINEQLIK VNSKGFLTIN SQPSVNAERS DSPTVGWGGP VGYVYQKAYL EFFCSKEKLD AVVEKCKALP SITYMAVNKG EQWVSNTAQA DVNAVTWGVF PAKEIIQPTI VDPASFNVWK DEAFETWSRS WANLYPEADP SRNLLEEVKN SYYLVSLVEN DYINGDIFAV FADL | CYT  CHL  NUCL  MIT  PLAS | 6  3  2  1  1 | CHL  MIT  SECR  OTHER | 0.07  0.07  0.17  0.84 | CHL  MIT  SECR  OTHER | 0  0.01  0.02  0.97 | CYT  CHL  PLAS | 0.92  0.05  0.01 | CYT |
| 4705 | TCP-1 chaperonin-like protein  *Arabidopsis thaliana* | 31% | MSVRVLNPNA EVLNKSAALH MTINAAKGLQ DVLKSNLGPK GTIKMLVGGS GDIKLTKDGN TLLKEMGAQK PQIQNPTAIM IAR**TAVAQDD ISGDGTTSTV IFIGELMK**QS ERCIDEGMHP R**VLVDGFEIA KR**ATLQFLDN FK**TPVVMGDE VDKEILK**MVA RTTLRTKLYE GLADQLTDIV VNSVLCIR**KP EEAIDLFMVE IMHMRHKFDV DTRLVEGLVL DHGSRHPDMK** RRAENCHILT CNVSLEYEK**S EINAGFFYSN AEQR**EAMVTA ERRSVDERVK KIIELKKKVC GDNDNFVVIN QK**GIDPPSLD LLAR**EGIIGL RRAKRRNMER LVLACGGEAV NSVDDLTPES LGWAGLVYEH VLGEEKYTFV EQVKNPNSCT ILIK**GPNDHT IAQIK**DAVRD GLRSVKNTIE DECVVLGAGA FEVAARQHLL NEVKKTVQGR **AQLGVEAFAN ALLVVPK**TLA ENAGLDTQDV IISLTSEHDK GNVVGLNLQD GEPIDPQLAG IFDNYSVK**RQ LINSGPVIAS QLLLVDEVIR** AGRNMRKPTA | CYT | 13 | CHL  MIT  SECR  OTHER | 0.05 0.24 0.22  0.35 | CHL  MIT  SECR  OTHER | 0  0.01  0  0.99 | CYT  MIT | 0.97  0.01 | CYT |
| 4707 | Pyrophosphate-dependent phosphofructokinase beta  Sub. PPi-PFKb  *Citrus paradisi* | 21% | MSPSLVANAD LSPVTSGTVK GRVASVYSEL QTSRIDHALP LPSVLKNPFK **IVDGPASSAA GNPDEIAK**LF PNLFGQPSAL LVPNGADAVR SDEKLK**IGVV LSGGQAPGGH NVISGIYDYL QDR**AKGSVLY GFRGGPAGIM KCKYVELTSN YIYPYR**NQGG FDMICSGRDK IETPEQFK**QA TETAVKLDLD GLVVIGGDDS NTNACLLAEH FRSKNLKTLV MGCPKTIDGD LKCKEVPASF GFDTACKIYA EMIGNVMIDA RSTGK**YYHFV R**LMGR**AASHI TLECALQTHP NITIIGEEVA AK**KQTLKNVT DYIVDIICKR AELGYNYGVI LIPEGLIDFI PEVQQLIAEL NEILAHEVVD EGGQWKKKLT KQSLQLFEFL PQAIQEQLML ERDPHGNVQV AKIETEKMLI QMVETELENR KQEGVNKGQF K**GQSHFFGYE GR**CGLPTNFD ATYCYALGYG AGALLHSGKT GLISSVGNLA APVEEWTVSG TALTALMDVE RRHGKFKPVI KK**AMVELDGT PFKK**FVSMRD EWALNNRYIS PGPIQFTGPT SGDVNHTLLL ELGAQV | CYT  CHL  MIT  PLAS | 7  4  1  1 | CHL  MIT  SECR  OTHER | 0.5 0.1 0.05  0.35 | CHL  MIT  SECR  OTHER | 0.99  0  0  0 | CYT  MIT  VAC | 0.93  0.04  0.01 | CYT/ CHL |
| 4809 | Uncharacterized protein , NCBI blastp - heat shock protein STI-like  *Glycine max* | 11% | MADEAKAKGN AAFSSGDYPA AIHHFSDAIA LAPTNHVLYS NR**SAAYASLQ NYTDALADAK** KTVELKPDWS KGYSRLGAAH LGLSQYGDAV SAYEKGLKID PNNEPLKSGL ADAQKALAAA SRPRSSAANP FGDAFSGPEM WARLTADPTT R**AYLQQPDFV K**MMQDIQRDP NNLNLHLK**DQ RIMQALGVLL NVK**IQTPPTG ADTDMPDSPS PSAAASERKR AAEAEPAKQP ESEPEPEPES MELTGEEKGA KQRKAEALKE KDAGNAAYKK KDFDTAIQHY TKALELDDED ISYLTNRAAV YLEMGKYEEC IKDCDKAVER GRELRSDFKM IARALTRKGN ALVKLAKCSK DYEPAIETYQ KALTEHRNPD TLKKLNEAEK AKK**ELEQQEY FDPK**LADEER EKGNEFFKQQ KYPEAVKHYT ESIRRNPKDP RAYSNRAACY TKLGAMPEGL KDAEKCIELD PTFVKGYTRK GAVQYFMKEY EKSLETYREG LKYDSNNQEL LEGIRTCIQQ INKASRGDLS PEELKERQAK AMQDPEIQNI LQDPVMR**QVL IDFQENPK**AA QEHTKNPMVM NKIQKLVSAG IVQMK | MIT  CYT  NUCL | 8  4  1 | CHL  MIT  SECR  OTHER | 0.14 0.120.13  0.76 | CHL  MIT  SECR  OTHER | 0  0.03  0.01  0.95 | CYT  NUCL | 0.99  0.01 | CYT |
| 4810 | Uncharacterized protein, NCBI blastp - heat shock protein STI-like  *Glycine max* | 12% | MADEAKAKGN AAFSSGDYPA AIHHFSDAIA LAPSNHVLYS NRSAAYASLK NYADALADAK **KTVELKPDWS K**GYSRLGAAH LGLSQYDDAI LAYKRGLEID PHNEPLKSGL ADAQKALAAA SRPRPSASNP FGDAFSGPEM WARLTADPTT R**AYLQQPDFV K**MMQDIQRDP NNLNLHLK**DQ RIMQALGVLL NVK**IQTPPTG ADTDMPDSPS PSAAASERKR AAEAEPAKQP EPEPEPEPVP MELTGEEKDA EQKKAEALKE KEAGNAAYKK KDFDTAIQHY TKALELDDED ISYLTNRAAV YLEMGKYEEC IKDCDKAVER GRELRSDFKM IARALTRKGN ALVKMAKCSK DYESAIETYQ KALTEHRNPD TLKKLNEAEK AKK**ELEQQEY FDPK**LADDER EKGNEFFKQQ KYPDAVKHYT ESIRRNPKDP RAYSNRAACY TKLGAMPEGL KDAEKCIELD PTFVKGYTRK GAVQYFMKEY DKALETYREG LKYDSNNQEL LEGIRTCIQQ INKASRGDLS PDELKER**QAK AMQDPEIQNI LQDPVMR**QSW VLLSMQVLID FQENPKAAQE HTK**NPMVMNK IQK**LVSAGIV QMK | MIT  CYT  NUCL | 8  4  1 | CHL  MIT  SECR  OTHER | 0.15 0.11 0.11  0.76 | CHL  MIT  SECR  OTHER | 0  0.03  0.01  0.96 | CYT  NUCL | 0.99  0.01 | CYT |
| 5004 | Proteasome subunit beta type  RCOM_1478370  *Ricinus commu*  *nis* | 11% | MSKSAIDVPP KGGFSFDLCK RNEMLAKKGV NLPSSYRKTG TTIVGLIFQD GVILGADTR**A TEGPIVCDKN CEK**IHYMAPN IYCCGAGTAA DTEAVTDMVS SQLQLHRYHT GRESR**VITAL TLLKK**HLFNY QGYVQAALVL GGVDCTGPHL HTIYPHGSTD TLPFATMGSG SLAAMAIFES KYREGLNRDE GIKLVTEAIC SGVFNDLGSG SNVDVCVITK GHKEYLR**NHL LPNPR**TYINP KGYVFSKKTE VLLTKITPLK EPSKEKEGVS EGGEAMEE | CYT  PLAS  PER | 11  1  1 | CHL  MIT  SECR  OTHER | 0.280.12 0.11  0.79 | CHL  MIT  SECR  OTHER | 0  0.04  0.01  0.94 | CYT  MIT EXTR | 0.94  0.04  0.01 | CYT |
| 5106 | Triosephosphate isomerase  *Solanum tubero*  *sum* | 33% | MAR**KFFVGGN WK**CNGTVEEV KKIVTTLSEA EVPSEDVVEV VISPPFVFLP LVKTLLRRDF SIAAQNCWVR KGGAFTGEVS AEMLVNLGIP WVILGHSERR LLLNESNDFV ADK**VAYALSQ GLKVIACVGE TLEQR**ESGST MAVVAAQTKA IAEQVMHWSN VVLAYEPVWA IGTGK**VATPA QAQEVHFELR K**WLHVNVGAE VAASTR**IIYG GSVNGANCKE LAAQPDVDGF LVGGASLKPE FIDIIK**SATV KTTAKSE | CHL | 13 | CHL  MIT  SECR  OTHER | 0.10 0.090.07  0.80 | CHL  MIT  SECR  OTHER | 0  0.02  0.03  0.94 | CYT  PER  VAC | 0.97  0.02  0.01 | CYT |
| 5107 | Gamma carbonic anhydrase 1, mitochondrial  *Arabidopsis thaliana* | 23% | MGTLGRAFYS VGFWIRETGQ ALDRLGCRLQ GKNYFREQLS RHR**TLMNVFD KAPIVDK**EAF VAPSASVIGD VHIGR**GSSIW YGCVLRGDVN TVSVGSGTNI QDNSLVHVAK** SNLSGKVHPT IIGDNVTIGH SAVLHGCTVE DETFIGMGAT LLDGVVVEKH GMVAAGALVR QNTR**IPSGEV WGGNPARFLR** KLTDEEIAFI SQSATNYSNL AQAHAAENAK PLNVIEFEKV LRKKHALKDE EYDSMLGIVR ETPPELNLPN NILPDKETKR PSNVN | CHL  CYT  MIT  NUCL | 6  3  3  1 | CHL  MIT  SECR  OTHER | 0.04 0.87 0.01  0.17 | CHL  MIT  SECR  OTHER | 0  0.24  0  0.75 | MIT  CHL  VAC | 0.93  0.06  0.01 | MIT |
| 5505 | UDP-sulfoquino vose synthase  RCOM_0797290  *Ricinus Commnis* | 24% | MAHLLSTSSS LKLSSSCSSY SLPLNQHATT FPTYFTLRTS RPLRKLVLQG ERPK**RCCVVH AASVPVSQEA PTR**SSSNSHQ TSGESFKSQR VMVIGGDGYC GWATALHLSK KGYEVAIVDN LIRRLFDQQL GLDSLTPISS IHNRLR**CWKS LTGK**TIELYI GDICDFEFLS ETFKSFEPDA VVHFGEQR**SA PYSMIDR**SR**A VFTQHNNVIG TLNVLFAIK**E FREECHLVK**L GTMGEYGTPN IDIEEGYITI THNGR**TDTLP YPKQASSFYH LSK**VHDSNNI AFTCK**AWGIR **ATDLNQGVVY GVR**TNETEMH EELYNR**FDYD GVFGTALNR**F CVQAAIGHPL TVYGKGGQTR GYLDIRDTVQ CVELAIANPA KPGEFRVFNQ FTEQFSVNEL ASLVTKAGEK IGLEVKTISV PNPRVEAEEH YYNAKHTKLI ELGLKPHLLS DSLLDSLLNF AIKFKDRVDT KQIMPSVSWK KIGVKPKTVA | CHL  MIT | 11  2 | CHL  MIT  SECR  OTHER | 0.880.010.07 0.06 | CHL  MIT  SECR  OTHER | 0.99  0  0  0 | PLAS  PER  CHL | 0.29  0.22  0.16 | CHL |
| 5602 | Enolase  *Ricinus communis* | 30% | MTTIKVVKAR QIFDSRGNPT VEVDVILSDG TLAR**AAVPSG ASTGIYEALE LR**DGGSDYLG KGVLKAVENV NSIIGPALIG KDPTEQTQID NFMVQELDGT VNEWGWCKQK LGANAILAVS LAVCKAGASV K**KIPLYQHIA NLAGNKTLVL PVPAFNVING GSHAGNK**LAM QEFMILPVGA SSFKEAMK**MG VEVYHHLK**AV IKK**KYGQDAT NVGDEGGFAP NIQENKEGLE LLK**TAIGKAG YTGKVVIGMD VAASEFYDNK DKTYDLNFKE ENNDGSEKIS GDSLKNVYKS FVTDYPIVSI EDPFDQDDWE HYSKLTAEIG EQVQIVGDDL LVTNPKRVNK GIREKTCNAL LLK**VNQIGSV TESIEAVK**MS KRAGWGVMAS HR**SGETEDTF IADLSVGLAT GQIK**TGAPCR SERLAK**YNQL LR**IEEELGSG AVYAGAKFRA PVAPY | CYT  MIT  CHL  PER | 9  2  1  1 | CHL  MIT  SECR  OTHER | 0.05 0.30 0.09  0.73 | CHL  MIT  SECR  OTHER | 0  0.03  0.01  0.97 | MIT  VAC  CYT | 0.82  0.06  0.05 | CYT/ MIT |
| 5701 | Chaperonin containing t-complex protein 1 gamma subunit, RCOM _ 0573520  *Ricinus communis* | 16% | MQAPVLVLKD SLKRESGTKV HHANIQASKA VADIIRTTLG PRSMLK**MLLD AAGGIVVTND GNAILR**ELDL AHPAAKSMIE LSRTQDEEVG DGTTSVIVLA GEMLHVAEAF IDK**SYHPTVI CR**AYIK**ALED AIAVLDK**IAM SIDVNDRATL LGLVKSCIGT KFTSQFGDLI ADLAIDATTT VGVDLGQGLX XDIKKYIKVE KVPGGQLEDS KVLKGVMFNK DVVAPGKMRR KIVNPR**IILL DCPLEYK**KGE NQTNAELVKE EDWAVLLKME EEYIQNMCMQ ILTFKPDLVI TEKGLSDLAC  HYLSRAGVGA IRRLRKTDNN RIAKACGAVI VNRPEELQES DVGTGAGLFE VKKIGDEFFS FIVDCKDPKA CTVLLRGASK DLLNEVERNL QDAMSVARNI LKNPKLLPGG GATELTVSAT LK**QKSSSVEG IEKWPYEAAA LAFEAIPRTL AQNCGVNVIR** TMTALQGKHA NGDNAWVGID GNTGEITDMK ERKIWDAYNV KAQTFKTAIE AACMLLRIDD IVSGIKKKQA PGAGPSKPKI ETEADADGEQ ILPD | CYT  PER  CHL | 9  3  1 | CHL  MIT  SECR  OTHER | 0.13 0.28 0.02  0.63 | CHL  MIT  SECR  OTHER | 0  0.04  0.01  0.95 | CYT  MIT | 0.97  0.02 | CYT |
| 5702 | Predicted protein  POPTRDRAFT_554969  NCBI blastp - sorting and assembly machinery (sam50) protein, putative  *Populus trichocarpa* | 7% | MIKNDDVSFT SSALKIAPFL HHQTKPSLPF FSQFVQTKLT FLDSLLTRTR FPNSPLLCSA SLSLTRPSSP GPDPKSLPIL CSASLSLSQS QLRDSTQSDS VVAQQKSGGA SGVHGPSRYD EER**VLISEVL VR**NKDGEELE RKDLEAEALA ALKACRANSA LTVREVQEDV HRVISSGYFC SCMPVAVDTR DGIRLVFQVE PNQEFHGLVC EGASVLPTKF LQDAFRGGYG KVVNIKQLDE VISSINSWYM ERGLFGMVSN AEILSGGIIR LQIAEAEVND ISIRFLDRKT GEPTKGKTKP ETILRQLTTK KGQVYSMLQG KRDVDTVLTM GIMEDVSFIP QPAEDTGKVD LIMNVVERPN GGFSAGGGIS SGFAYSHRNV FGRNQKLNIS LER**GQIDSIF RINYTDPWIE GDDKR**TSRTI MVQNSRTPGN LVHGNQPVNN SLTIGRVAAG IEFSRPLRPK WSGTVGLIFQ HAGARNEKGD PKIKDHYNSP LTASGKNHDD MLLAKFESVY TGSGDHGSSM FVFNMEQGLP LWPEWLFFNR VNTRARKGVE IGPALCLLSL SGGHVMGNFS PHEAFAIGGT NSVRGYEEGA VGSGRSYAVG  SGEISFPVLG PVEGVFFADY GTDLGSGPSV PGDPAGAR**LK PGSGYGYGFG IRVDSPLGPL R**LEYAFNDRH TKRFHFGVGH | NUCL  CYT | 10  3 | CHL  MIT  SECR  OTHER | 0.23 0.03 0.01  0.58 | CHL  MIT  SECR  OTHER | 0.21  0  0  0.97 | CHL  MIT  PLAS | 0.90  0.05  0.02 | NUC/  CHL |
| 5806 | Aconitate hydratase, cytoplasmic  *Cucurbita maxima* | 17% | M**AAENPFKEN LTSLPKPGGG EFGK**YYSLPS LNDPRIDRLP YSIR**ILLESA IR**NCDNFQVK KEDVEK**IIDW ENSSPKQVEI PFKPARVLLQ DFTGVPAVVD LACMRDAMNK LGSDSNK**INP LVPVDLVIDH SVQVDVAR**SE NAVQANMELE FQR**NKERFAF LKWGSNAFQN MLVVPPGSGI VHQVNLEYLG RVVFNTSGLL YPDSVVGTDS HTTMIDGLGV AGWGVGGIEA EAAMLGQPMS MVLPGVVGFK LSGKLRNGVT ATDLVLTVTQ MLRKHGVVGK FVEFYGDGME ELSLADR**ATI ANMSPEYGAT MGFFPVDHVT LQYLK**LTGRS DETVSMIEAY LRANK**MFVDY KEPQQEK**VYS SYLQLDLTDV EPCISGPK**RP HDRVPLK**EMK SDWHACLDNK VGFKGFAIPK EAQENVAKFS FHGQPAELKH GSVVIAAITS CTNTSNPSVM LGAALVAKKA CELGLQVKPW VKTSLAPGSG VVTKYLLKSG LQPYLNQQGF HIVGYGCTTC IGNSGDLDES VSAAISDNDI VAAAVLSGNR NFEGRVHPLT RANYLASPPL VVAYALAGTV DIDFEKEPIG KGKDGKDVYF RDIWPSTEEI  AEVVQSSVLP DMFKSTYESI TKGNPMWNQL SVPSGTLYSW DPNSTYIHEP PYFKNMTMDP PGAHGVKDAY CLLNFGDSIT TDHISPAGSI HKDSPAAKYL LERGVDRK**DF NSYGSRR**GND EVMARGTFAN IRLVNKLLDG EVGPKTVHVP TGEKLSVFEA AEKYKSAGQD TIVLAGAEYG SGSSRDWAAK GPMLLGVKAV IAKSFERIHR SNLVGMGIIP LCFKSGEDAD SLGLTGHERY TIDLPDDISK IRPGQDVTVT TDSGKSFTCT VRFDTEVELA YFNNGGILPY VIRNLIKQ | CYT  CHL  NUCL | 6  5  2 | CHL  MIT  SECR  OTHER | 0.07 0.22 0.05  0.89 | CHL  MIT  SECR  OTHER | 0  0.01  0.01  0.98 | CYT  CHL  VAC | 0.53  0.34  0.04 | CYT |
| 6302 | Alcohol dehydrogenase  Adh1-1a  *Pyrus communis* | 26% | DVYFWEAKGQ NPLFPRIYGH EAGGIVESIG EGVTDLKAGD HVLPVFTGEC KDCAHCK**SEE SNMCDLLR**IN TDRGVMLSDG KSRFSIKDKP IYHFVGTSTF SEYTVVHVGC LAKINPLAPL DKVCLLSCGI STGLGATLNV AKPK**KGSTVA VFGLGAVGLA AAEGAR**FSGA SRIIGVDLHS GRFEEAK**KFG VTEFVNPKEH KKPVQEVIAE LTNGGVDR**SI ECTGSVEFMI SAFECVHDGW GVAVLVGVPH KEAVFK**THPL NFLNERTLKG TFFGNYKPR**T DIPSVVEKYM NKELQLEKFI THKVPFSEIN KAFEYMLKGE GLRCIIHMED | CYT  PLAS | 12  1 | CHL  MIT  SECR  OTHER | 0.090.150.13  0.85 | CHL  MIT  SECR  OTHER | 0  0.03  0.07  0.9 | CYT  PER  VAC | 0.84  0.14  0.01 | CYT |
| 6304 | Dihydroflavonol 4-reductase  *Paeonia suffruticosa* | 16% | MEAVTECDAE TVCVTGAAGF IGSWLVMRLL EHGYVVRATV RDPENMRKVK HLLDLPKADT HLTLWKADLL VDGSFDEAIK **GCTGVFHVAT PMDFESK**DPE NEVIKPTIDG MLSIMRAWAK AKTVRRVVFT SSAGTVNVQQ HQQSVYDETC WSDMEFIQTK K**MTGWMYFVS K**ILAEQAAWK FAKENSIDFI SIIPTLVVGP FLMPSMPPSL VTALSLITGN EAHYSIIKQG QFVHLDDLCN AHIYLFEHPK VEGR**YICSSH DATIFSLAKM LR**DKYPEYNV PTEFKDVDES LKSVSFSSKK LMDSGFEFK**Y NLEDMFVGAI ETCR**EKGLLP LPVEKNHFLG AIETCRGKGL LPLPIERNHV NDTI | CHL  NUCL  CYTO  PLAS  EXTR  ENDO | 4  2  2  2  2  2 | CHL  MIT  SECR  OTHER | 0.06 0.150.27  0.63 | CHL  MIT  SECR  OTHER | 0  0  0.02  0.97 | PLAS  CYT  CHL | 0.46  0.19  0.11 | PLAS/ CYT |
| 6403 | Naringenin-chalcone synthase  CHS1  *Juglans nigra x*  *Juglans regia* | 37% | MVTVEDVRRA QR**AEGPATVM AIGTATPPNC VDQSAYPDYY FR**ITNSEHKT ELKEKFKRMC GKSMIKKRYM HLTEEILKEN PNVCAYMASS LDARQDMVVV EVPKLGKEAA TK**AIKEWGQP K**SK**ITHLVFC TTSGVDMPGA DYQLTKLLGL RPSVKRLMMY QQGCFAGGTV LR**LAKDLAEN NK**GARVLVVC SEITAVTFR**G PSDTHLDSLV GQALFGDGAA ALIVGADPVP GVEKPLFELV SAAQTILPDS DGAIDGHLR**E VGLTFHLLK**D VPGLISKNIE KSLVEAFQPL GITDWNSLFW IAHPGGPAIL DQVESK**LELK PEKLR**ATR**HV LSEYGNMSSA CVLFILDEMR K**KSAEDRLKT TGEGLEWGVL FGFGSGLTVE TVVLHSVSA | CYT  PER | 10  3 | CHL  MIT  SECR  OTHER | 0.03 0.48 0.06  0.74 | CHL  MIT  SECR  OTHER | 0  0  0.01  0.98 | CYT  CHL  MIT | 0.93  0.05  0.01 | CYT |
| 6601 | Shikimate dehydrogenase, putative  *Ricinus communis* | 29% | MSDLQLSNSS VQSSPTLLCT PLMGTTVDQM LLEMRKATEI SADVVEIRLD CLRNLNPRQD LEILIKQSPL PTLVTYRPIW EGGQYEGDET KRQDALRFAM QLGANYVDVE LEVAHEFNNS IYGKKPDNFK **VIVSSHNFHN TPSSEAIANL VAR**IQATGAD IVK**IATTALD ITDCAR**IFQI MVHCQVPVIG IVMGERGLIS RLLSPK**FGGY LTYGALEAGA ISAPGQPTAK DLLDLYNFR**L IRPDTK**VYGI IGKPVGHSKS PLLFNAAFK**S VGLNAVYVHF LVDDVEKFFS TYSSVDFASG CSCTIPHKEV ALKCMDEIDP IAKKIGAINN IIRRPDATLM AYNTDYIGAI DAIEDGLREL NGAVPAGTSP LAGK**LFVVLG AGGAGK**SLAY GAAQKGARVV VANRTFERAK ELADKVNGQA MTLDEVQNFH PEEGMVLANT TSVGMKPNID ATPLAKHALK **HYCVVFDAIY TPK**DTRLLRE AK**ESGAVIVY GTEMLIRQGF EQYKNFTGLP APEELFR**QLM EKHA | CHL  EXTR  NUCL  CYT  ENDO | 5  3  2  2  2 | CHL  MIT  SECR  OTHER | 0.28 0.08 0.18  0.49 | CHL  MIT  SECR  OTHER | 0  0  0.01  0.98 | CYT  PER  MIT | 0.92  0.05  0.02 | CYT |
| 6602 | Aldehyde dehydrogenase ALDH2B4_V2  *Vitis*  *vinifera* | 7% | MYETDVGIIT GKTFPTFDPR TGEVIANVAE GDAEDINRAV SAAR**KAFDEG PWPR**MSPYER SRILLRFADL AEKHNDELAA LETWNNGKPY EQAAKAELPL FVRLFRYYAG WADKIHGLTV QADGPHHVQI LHEPIGVAGQ IIPWNFPLMM FAWKVGPALA CGNTIVLKTA EQTPLTALFA AKLFHEAGLP PGILNIVSGY GPTAGAALAS HMDVDKIAFT GSTDTGKIVQ ELASKSNLKP VTLELGGKSP FIVCEDADID QAVELAHFAL FFNQGQCCCA GSRTFVHESV YDEFIEKAKA RALSRTVGDP FKKGIEQGPQ IDPEQFAKVL RYIRSGIESN ATLECGGGRI GSKGYFVQPT VFSNVQDDML IAKDEIFGPV QSILKYKDLD EVIRRANSTR **YGLAAGVFTK** NINTANTLTR ALRVGTVWVN CFDVFDAAIP FGGYKMSGVG R**EKGIYSLNN YLQVK**AVITP LKNPAWL | CYT  CHL  CYSK | 7  3  3 | CHL  MIT  SECR  OTHER | 0.1 0.05 0.12  0.95 | CHL  MIT  SECR  OTHER | 0  0.03  0.06  0.9 | CYT  PER  MIT | 0.87  0.11  0.01 | CYT |
| 6801 | Vitamin-b12 independent methionine synthase  *Populus trichocarpa* | 19% | MASHVVGYPR MGPKRELK**FA LESFWDGK**SS AEDLQNVAAE LRSSIWKQMS DAGIKFIPSN TFSCYDQVLD TTAMLGAVPP RYGWNGGEIG FDVYFSMARG NASVPAMEMT KWFDTNYHYI VPELGPEVNF SYASHKAVIE YKEAK**ALGVD TVPVLVGPVS YLLLSKPAK**G VEKSFSLLSL IDKILPVYQE VVAELKAAGA TWIQFDEPKL VMDLGAHELQ AFTHAYSALE ASLSGLNVLI ETYFADVPVE AYKTLISLKC VTGFGFDLVR GTKTLDLIKG GFPSGK**YLFA GVVDGR**NIWA NDLASSLSVL HALEGIVGKD KLVVSTSCSL LHTAVDLVNE PKLDKEIKSW LAFAAQKVVE VNALAKALSG QKDGAFFSAN AAAQASRKSS PRVTNEAVQQ AAAALKGSDH RRATNVSDRL DAQQK**KLNLP ILPTTTIGSF PQTLELRR**VR REYKAKK**VSE EDYVEAIK**KE INKVVK**LQEE LDIDVLVHGE PER**NDMVEYF GEQLSGFAFT ANGWVQSYGS RCVKPPIIYG DVSRPKAMTV FWSSLAQSMT KRPMK**GMLTG PVTILNWSFV RNDQPR**FETC YQIALAIKDE VEDLEKAGIT  VIQIDEAALR EGLPLRKSEH AFYLNWAVHS FRITNCGVED STQIHTHMCY SNFNDIIHSI IDMDADVITI ENSR**SDEKLL SVFREGVKYG AGIGPGVYDI HSPR**IPSEEE IADRVEKMLA VLECNILWVN PDCGLKTRKY AEVKPALSNM  VAAAKRLRTK FASSQ | CYT  CHL  MIT  PER | 5  4  2  2 | CHL  MIT  SECR  OTHER | 0.050.65 0.03  0.61 | CHL  MIT  SECR  OTHER | 0  0.06  0  0.93 | PER  CHL  CYT | 0.52  0.16  0.15 | CYT/  PER? |
| 7002 | Proteasome subunit beta type  *Picea sitchensis* | 17% | MKLDLSGLEP ATLHQLSNDN LCDVFSPTPS FEIPNDADFD GFQKAAVQMV KPAKGTTTLA FIFKEGIMVA ADSRASMGGY ISSQSVKKII EINPYLLGTM AGGAADCQFW HRNLGIKCRL HELANKRRIS VTGASK**LLAN ILYSYRGMGL SVGTMIAGWD ETGPGLYYVD SEGGR**LKGTR FSVGSGSPYA YGVLDSGYRW DMPVEEAAEL AR**RSIYHATF R**DGASGGVAS VYYVGPQGWK KLSGDDVAEL HFNYYPVTEN VVEQEMVEVT A | CYSK | 13 | CHL  MIT  SECR  OTHER | 0.160.050.15 0.87 | CHL  MIT  SECR  OTHER | 0  0.02  0.02  0.95 | CHL  MIT  CYT | 0.43  0.17  0.13 | CYT |
| 7302 | Formate dehydrogenase Oxidoreductase, acting on the CH-OH group of donors, with NAD or NADP  *Quercus robur* | 41% | MAGAATSAIK SVLTRHLHAS PGSK**KIVGVF YK**ANENAALN PNFVGCVEGS LGIR**DWLESQ GHQYIVTDDK** EGPNSELEK**H IPDLHVLITT PFHPAYVTAE R**ITKAKNLQL LLTAGIGSDH IDLPAAAAAG LTVAEVTGSN VVSVAEDELM RILILVR**NFL PGYHQAISGE WNVAAISHR**A YDLEGK**TVGT VGAGR**IGK**LL LQRLKPFNCN LLYHDRLK**MD PELENQIGAN FEEDLDAMLP K**CDIIVINTP LTDKTRGLFD KDR**IAKCK**KG VLIVNNAR**GA IMDIQAVADA CSSGHVAGYS GDVWFPQPAP KDHPWRYMPN HAMTPHISGT TIDAQLR**YAA GTKDMLERYF KGEEFPSQNY IVK**GGKLASQ YQ | CYT  MIT  PER  CHL | 5  5  2  1 | CHL  MIT  SECR  OTHER | 0.320.35 0.05  0.23 | CHL  MIT  SECR  OTHER | 0.89  0.28  0  0 | CYT  MIT  VAC | 0.83  0.14  0.01 | ? |
| 7306 | Fructose-bisphosphate aldolase, cytoplasmic isozyme 2  *Pisum sativum* | 13% | MSHFKSKYHD ELIANAAYIG TPGK**GILAAD ESTGTIGKR**L SSINVENVES NRQALRELLF TASWLFLQYL SGVILFEETL YQKTAAGKPF VDVLNEAGVL PGIKVDKGTV ELAGTDGETT TQGLDGLGAR CRK**YYEAGAR** FAKWRAVLKI GANEPSEHSI HENAYGLARY AVICQENGLV PIVEPEILVD GSHDILKCAA ITERVLAATY KALSDHHVIL EGTLLKPNMV TPGSDAPK**VA PEVIAEHTVR** ALQRTVPAAV PAVVFLSGGQ SEEEASVNLN AINQIK**GKKP WTLSFSFGR**A LQQSTLKAWG GKTENVKAAQ DALLTRAKAN SEATLGTYKG ASNLGAGASE SLHVKDYKY | CYT  CHL  PER | 9  2  2 | CHL  MIT  SECR  OTHER | 0.09 0.06 0.07  0.79 | CHL  MIT  SECR  OTHER | 0  0  0.05  0.94 | MIT  CYT  PER | 0.33  0.24  0.14 | CYT |
| 7401 | Chalcone synthase  *Malus domestica* | 38% | MVTVEEVRKA QR**AEGPATVF AIGTATPPNC VDQATYPDYY FR**ITNSEHKA ELKEKFQRMC DKSMIKK**RYM YLTEEILK**EN PSVCEYMAPS LDAR**QDMVVV EVPRLGKEAA TKAIKEWGQP KSKITHLVFC TTSGVDMPGA DYQLTKLLGL RPSVKRLMMY QQGCFAGGTV LR**LAKDLAEN NKGAR**VLVVC SEITAVTFR**G PSDTHLDSLV GQALFGDGAA AVIIGADPVP EVEKPLFELV SAAQTILADS DGAIDGHLR**E VGLTFHLLKD VPGLISK**NIE KSLNEAFKPI GISDWNSLFW IAHPGGPAIL DQVEAKLALK PEKLEATRQV LSDYGNMSSA CVLFILDEVR RKSAEKGLET TGEGLEWGVL FGFGPGLTVE TVVLHSVAA | CYT  PER | 11  3 | CHL  MIT  SECR  OTHER | 0.03 0.330.09  0.80 | CHL  MIT  SECR  OTHER | 0  0  0.03  0.96 | CYT  CHL  MIT | 0.94  0.05  0.01 | CYT |
| 7402 | Glutamate dehydrogenase  POPTRDRAFT_826140  *Populus tricho*  *carpa* | 25% | MNALVATNRN FKRAAKLLGL DSKLEK**SLLI PFREIKVECT IPKDDGTLAS FVGFR**VQHDN ARGPMKGGIR YHPEVDPDEV NALAQLMTWK **TAVANIPYGG AK**GGIGCNPG ELSVSELERL TRVFTQKIHD LIGIHTDVPA PDMGTGPQTM AWILDEYSKF HGYSPAVVTG KPIDLGGSLG RDAATGR**GVL FATEALLKEH GK**TISGQRFV IQGFGNVGAW AAQLISEQGG K**IVAVSDITG AIK**NNKGLDI PSLLKHANEH KGVKGFHGGD PIDPKSILVE DCDILIPAAL GGVINRENAS DIKSK**FIIEA ANHPTDPEAD EILTK**KGVVI LPDIFANSGG VTVSYFEWVQ NIQGFMWDEE KVNNELKNYM TRGFKDVKEM CK**THNCDLRM GAFTLGVNR**V ARATVLRGWG A | CYT  MIT  CHL | 6  3.5  3.5 | CHL  MIT  SECR  OTHER | 0.07 0.57 0.080.22 | CHL  MIT  SECR  OTHER | 0  0.99  0  0 | CYT  MIT  PER | 0.87  0.09  0.03 | CYT/ MIT |
| 7408 | Formate dehydro-genase  mitochondria  *Quercus robur* | 38% | MAGAATSAIK SVLTRHLHAS PGSK**KIVGVF YK**ANENAALN PNFVGCVEGS LGIR**DWLESQ GHQYIVTDDK** EGPNSELEK**H IPDLHVLITT PFHPAYVTAE R**ITKAKNLQL LLTAGIGSDH IDLPAAAAAG LTVAEVTGSN VVSVAEDELM RILILVR**NFL PGYHQAISGE WNVAAISHR**A YDLEGKTVGT VGAGRIGK**LL LQRLKPFNCN LLYHDR**LKMD PELENQIGAN FEEDLDAMLP K**CDIIVINTP LTDKTRGLFD KDR**IAKCK**KG VLIVNNAR**GA IMDIQAVADA CSSGHVAGYS GDVWFPQPAP KDHPWRYMPN HAMTPHISGT TIDAQLR**YAA GTKDMLERYF KGEEFPSQNY IVK**GGKLASQ YQ | CYT  MIT  PER  CHL | 5  5  2  1 | CHL  MIT  SECR  OTHER | 0.32 0.35 0.05  0.23 | CHL  MIT  SECR  OTHER | 0.89  0.28  0  0 | CYT  MIT  VAC | 0.83  0.14  0.01 | CYT/ MIT |
| 7605 | 6-phospho-fructokinase 3  PFK3  *Arabidopsis thaliana* | 22% | MSTVESSKPK IINGSCGYVL EDVPHLSDYL PGLPTYPNPL QDNPAYSVVK QYFVDADDSV PQKIVVHKDG PRGIHFRRAG PRQKVYFESD EVHACIVTCG GLCPGLNTVI REIVSSLSYM YGVKR**ILGID GGYRGFYAK**N TVSLDSKVVN DIHKRGGTIL GTSRGGHDTT K**IVDSIQDRG INQVYIIGGD GTQR**GASVIF EEIRRRGLKV AVIGIPK**TID NDIPVIDKSF GFDTAVEEAQ R**AINAAHVEA ESIENGIGVV KLMGR**YSGFI AMYATLASR**D VDCCLIPESP FYLEGEGGLF EYIEKRLKES GHMVLVIAEG AGQDLMSKSM ESMTLKDASG NKLLKDVGLW LSQSIKDHFN QKKMVMNLK**Y IDPTYMIR**AV PSNASDNVYC TLLAQSAVHG AMAGYTGYIS GLVNGRQTYI PFYRITEKQN HVVITDRMWA RLLSSTNQPS FLGPKDVFDN K**EKPMSALLD DGNCNGVVDV PPVTK**EITK | CYT  CYSK  NUCL | 7  4  2 | CHL  MIT  SECR  OTHER | 0.07 0.09 0.16  0.87 | CHL  MIT  SECR  OTHER | 0  0  0.03  0.96 | CYT  CHL  PER | 0.9  0.05  0.02 | CYT |
| 7701 | Malic enzyme  OB01G39450  *Oryza brachyantha* | 6% | MVASLPAARR ARVLLCTLAR ASPESPLYQL RVVASRTAFR ETARRCWLRW SLCRPGARTV SGMAGRGEGN DAATVGVVTG GVEDAYGEDR ATEDQPITPW AVCVASGHSL LRDPRHNKGL SFTEKERDAH YLRGLLPPVV LPQELQEKRL LQNMRQFPVP LQRYMALMDL QERNERLFYK LLIDNVEELL PVVYTPTVGE ACQKYGSIFR **RPQGLYISLK** EKGRILEVLR NWPEK**SIQVV VVTDGER**ILG LGDLGCQGMG IPVGKLALYT ALGGVRPSAC LPITIDVGTN NEDLLK**DEFY IGLR**QKRATG QEYSDLLNEF MDAVKQNYGQ KVLVQFEDFA NHNAFTLLEK YRANNLVFND DIQGTAAVVL AGLIAGQKFV GGTLADHTFL FFGAGEAGTG IAELVALEIS NQSKIPVEEA RKKIWLLDSK GLIVSSRKDS IQPFKK**RYAH EHEPVK**NLLD AVKAIKPTAL IGSAGVGQSF TKEVIEAMSS INERPIILAL SNPTSQSECT AEQAYSWSKG RAIFGSGSPF DPVKYNNKLF VPAQANNAYI FPGFGLGVVI SGAIRVKDEM ILAAAEALAD QVTPDHIDKG LIYPPFSCIR  KISANIAARV AAKAYDLGLA SHLLRPKDLV NYAESCMYSP VYRSYR | CHL  MIT | 10.5  3.5 | CHL  MIT  SECR  OTHER | 0.340.65 0.01 0.01 | CHL  MIT  SECR  OTHER | 0.87  0.68  0  0 | MIT  PER  VAC | 0.93  0.05  0.01 | CHL/ MIT |
| 8202 | Putative uncharacterized protein  SELMODRAFT_107855  *Selaginella*  *moellendorffii* | 11% | MVKGFKVYTP GGPEVLKYEE LDVGEPGEGQ VKIRIK**AVGL NFIDVYFRK**G VYGAKMPYVP GFESAGEVIA IGPGVTDAKV GDKVGCMGAF GAYAEEQIVP ARALVPIPES VDFIKAGAIL LK**GMTAQMLL R**KTFKVGPKH TILVHAAAGG VGSLLCQWAR **SLGAQVIGCV SSEEK**ARQAT EDGCHHVIIY SKEDFGKRVM AITNNSGVHV VYDSVGRDTF QGSLDCLATR GYMISYGQSS GAPDPFTMSI LAPKSLFLTR PSLIQYTTNR DELLEIANDL FHNVEAGVLK VRVNQTYPLS DAAKAHDDIE NRRTTGSTVL IP | CYT  CYSK  PER | 6  5  2 | CHL  MIT  SECR  OTHER | 0.03 0.11 0.24  0.91 | CHL  MIT  SECR  OTHER | 0  0.01  0.04  0.95 | CYT  PER  MIT | 0.95  0.02  0.01 | CYT |
|  |  |  |  |  | |  | |  |  |  |  |  |

Localisation prediction algoritms:

Target P: Predicting subcellular localization of proteins based on their N-terminal amino acid sequence. Olof Emanuelsson, Henrik Nielsen, Søren Brunak and Gunnar von Heijne. J. Mol. Biol., 300: 1005-1016, 2000, Web page - <http://www.cbs.dtu.dk/services/TargetP/>

PredSL: Petsalaki EI, Bagos PG, Litou ZI, Hamodrakas SJ. PredSL: a tool for the N-terminal sequence-based prediction of protein subcellular localization. Genomics Proteomics Bioinformatics. 2006 Feb;4(1):48-55. web page - <http://aias.biol.uoa.gr/PredSL/input.html>

MultiLoc: A. Hoglund, P. Donnes, T.Blum, H.-W.Adolph, O.Kohlbacher. MultiLoc: prediction of protein subcellular localization using N-terminal targeting sequences, sequence motifs and amino acid composition. Bioinformatics. 22 no. 10 2006, 1158–1165, doi:10.1093/bioinformatics/btl002 <http://abi.inf.uni-tuebingen.de/Services/MultiLoc/>

WolfPSORT: <http://www.genscript.com/psort/wolf_psort.html> Horton, P., Park, K.J., Obayashi, T., Fujita, N., Harada, H., Adams-Collier, C.J., and Nakai, K. (2007). WoLF PSORT: Protein localization predictor. *Nucleic Acids Res.* 35, 585-587.
