# Supplementary material for: 2-DE proteomics analysis of drought treated seedlings of Quercus ilex supports a root active strategy for metabolic adaptation in response to water shortage
Source: Front Plant Sci. 2015 Aug 14;6:627. doi: 10.3389/fpls.2015.00627 (PMC4536546; doi:10.3389/fpls.2015.00627)

**Supplementary material**

**Figure S1** - Experimental design.

→ time course

| C0 | C10 | C20 | C30 |
| --- | --- | --- | --- |
|  | D10 | R10 |  |
|  |  | D20 | R20 |

Light Blue – optimal water supply (perlite: H_2_0 1:3 w/w), Light red – stress by water limitation (perlite:H_2_0 1:1 w/w). C-control plants, C0, C10, C20, C30 – the respective age controls. D-plants subjected to water limitation treatment for 10 days (D10) or 20 days (D20). R – recovery by resuming optimal water supply for 10 days after 10 days (R10) or 20 days (R20) of water stress treatment.

**Figure S2** – Representative pictures of plants


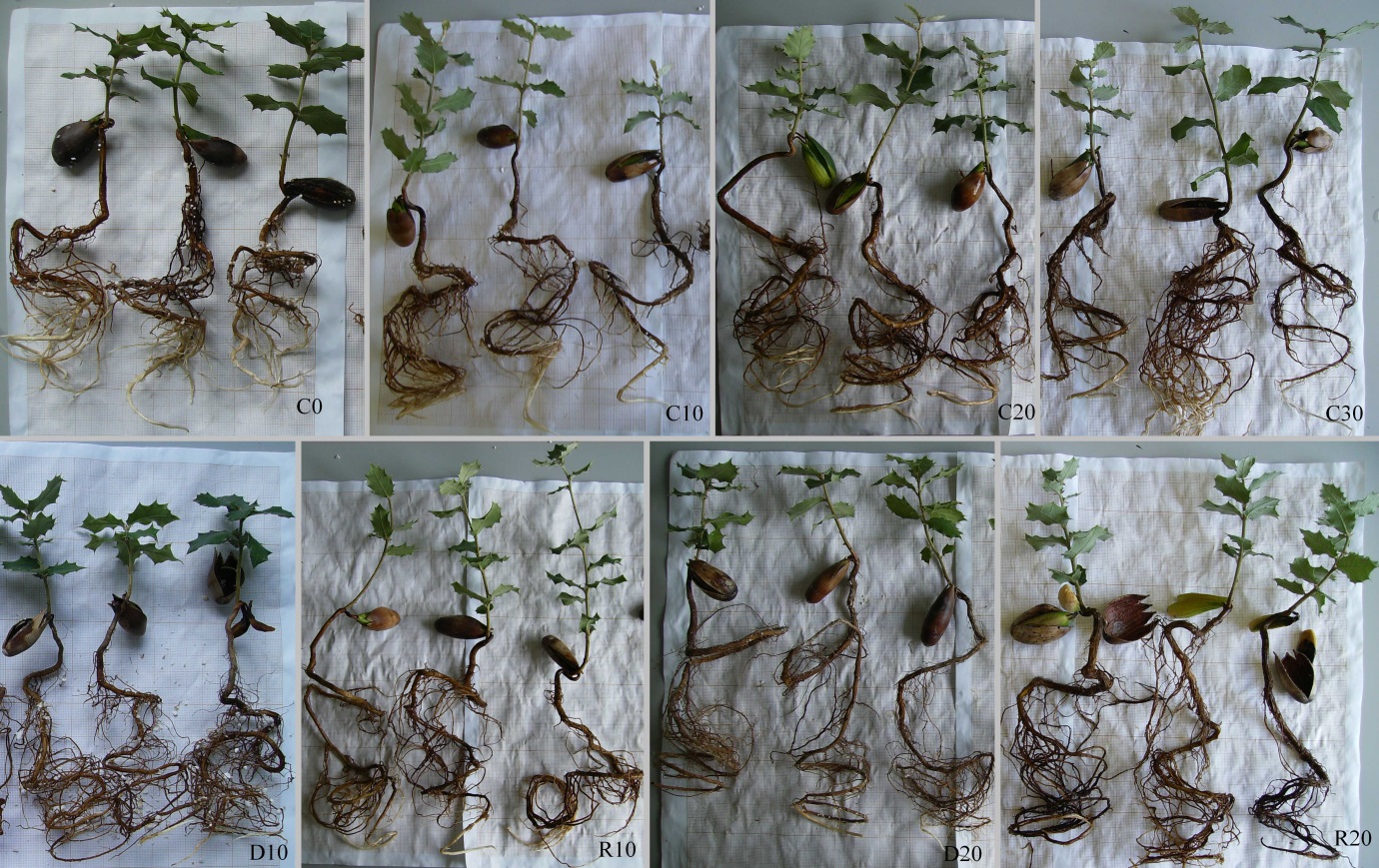


C-control plants, C0, C10, C20, C30 – the respective age controls. D-plants subjected to water limitation treatment for 10 days (D10) or 20 days (D20). R – recovery by resuming optimal water supply for 10 days after 10 days (R10) or 20 days (R20) of water stress treatment.

**Figure S3** –2-DE gel images of root protein extracts from control (C10, C20), drought treated (D10, D20) and recovered (R10) plants. First dimension IEF, second dimension 12% SDS-PAGE. On the left – positions of the molecular markers.


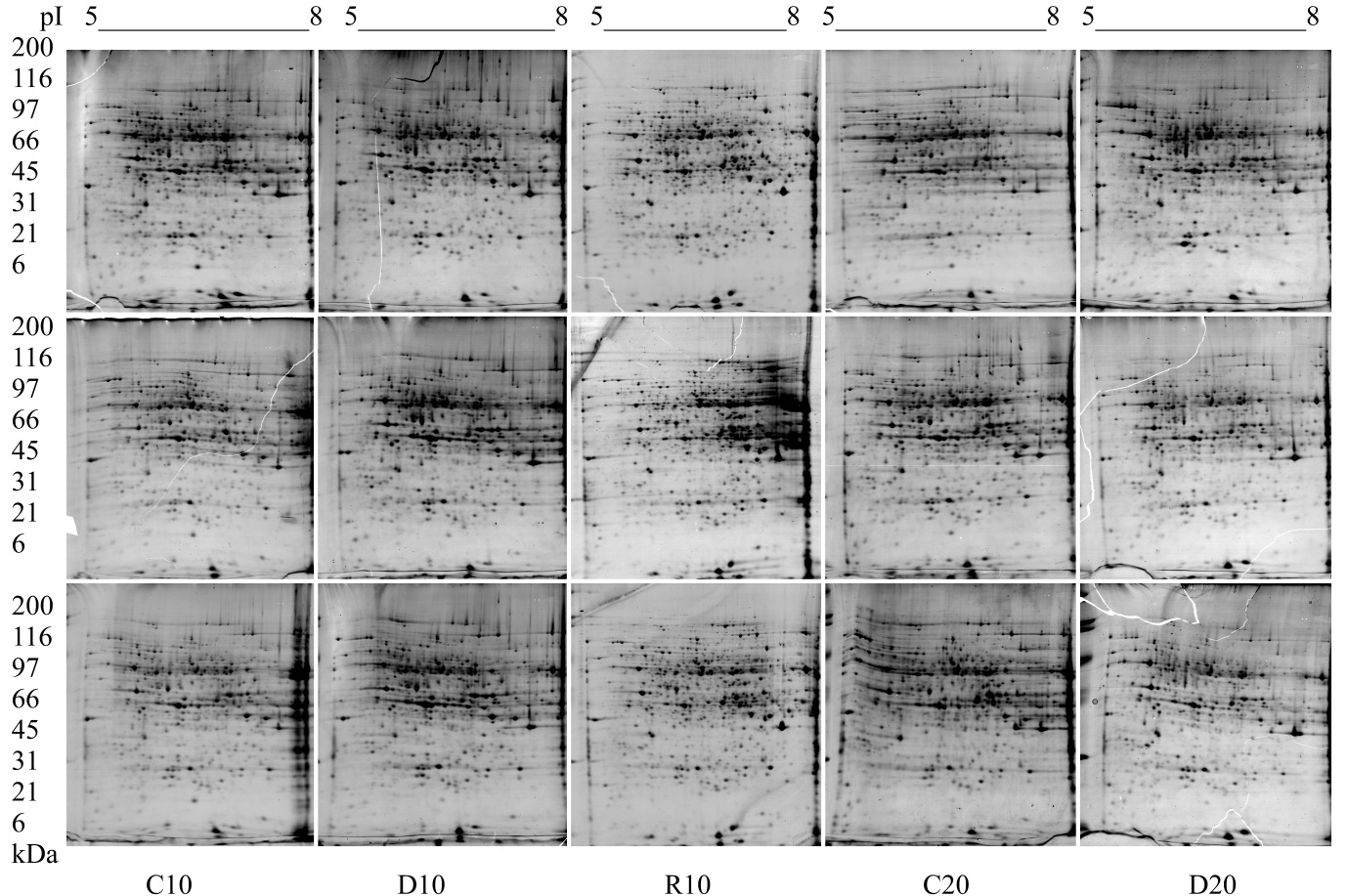

Supplement: Supplementary file 3 [file DataSheet1.DOCX]
